# SETD1A Regulates Glycolysis and Senescence of Nucleus Pulposus Cells via H3K4me3–HELZ2/PPARα‐HIF1α Axis to Drive Intervertebral Disc Degeneration

**DOI:** 10.1002/advs.75105

**Published:** 2026-03-31

**Authors:** Jiawei Fu, Xue Leng, Jiang Long, Zhengao Liao, Xiaoqin An, Xuezheng Ai, Dan Long, Changqing Li, Bo Huang, Yue Zhou, Shiwu Dong, Chencheng Feng

**Affiliations:** ^1^ Department of Orthopedics Xinqiao Hospital Army Medical University Chongqing P. R. China; ^2^ Chongqing Municipal Health Commission Key Laboratory of Precise Orthopedics Chongqing P. R. China; ^3^ Department of Biomedical Materials Science College of Biomedical Engineering Third Military Medical University Chongqing P. R. China

**Keywords:** cell senescence, glycolysis, helicase with zinc finger 2 (HELZ2), histone H3 lysine 4 trimethylation (H3K4me3), intervertebral disc degeneration (IDD), SET domain‐containing 1A (SETD1A)

## Abstract

Intervertebral disc degeneration (IDD) is a major cause of lower back pain, but its molecular mechanisms remain unclear. Epigenetic regulation is critical in IDD pathogenesis. This study explored the roles of SET domain‐containing 1A (SETD1A) and histone H3 lysine 4 trimethylation (H3K4me3) in IDD. Using human nucleus pulposus (NP) tissues, animal models, cultured nucleus pulposus cells (NPCs), and high‐throughput sequencing, we found that H3K4me3 was significantly decreased in degenerative NP tissues. H3K4me3 loss promoted NPC senescence, and SETD1A acted as a key upstream regulator. SETD1A knockdown accelerated NPCs senescence and aggravated IDD, whereas SETD1A overexpression exerted protective effects. Mechanistically, SETD1A downregulation reduced H3K4me3 enrichment at the helicase with zinc finger 2 (HELZ2) promoter, inhibiting HELZ2 transcription and HELZ2/peroxisome proliferator‐activated receptor alpha (PPARα) complex function. This cascade downregulated hypoxia‐inducible factor 1‐alpha (HIF1α), impaired glycolytic metabolism, and induced NPCs senescence. SETD1A serves as a key epigenetic regulator via the H3K4me3–HELZ2/PPARα–HIF1α axis, representing a promising therapeutic target for IDD.

## Graphical abstract

SETD1A is a key epigenetic regulator in NPCs during IDD. In normal NPCs, it sustains H3K4me3–HELZ2/PPARα–HIF1α signaling to maintain glycolytic energy metabolism and proliferation. In degenerated NPCs, reduced SETD1A disrupts this axis, impairing glycolysis and accelerating senescence, highlighting a promising therapeutic target for IDD.

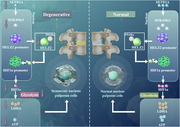

**Abbreviations**
µCT: micro‐computed tomography
2‐NBDG: 2‐deoxy‐2‐[(7‐nitro‐2,1,3‐benzoxadiazol‐4‐yl)amino]‐D‐glucose
AAV: Adeno‐associated virus
ACAN: aggrecan
ANOVA: analysis of variance
CCND2: cyclin D2
ChIP‐qPCR: chromatin immunoprecipitation quantitative polymerase chain reaction
ChIP‐seq: chromatin immunoprecipitation sequencing
cKO: conditional knockout
COL1A1: Collagen I
COL2A1: Collagen II
COMPASS: complex proteins associated with SET1
DEGs: differentially expressed genes
DMOG: dimethyloxalylglycine
ECM: extracellular matrix
GLUT1: glucose transporter 1
GO: Gene Ontology
GW6471: a selective PPARα antagonist
H&E: hematoxylin–eosin
H3K4me3: histone H3 lysine 4 trimethylation
HELZ2: helicase with zinc finger 2
HIF1α: hypoxia‐inducible factor 1‐alpha
IDD: intervertebral disc degeneration
IP: immunoprecipitation
IQR: interquartile range
IVD: intervertebral disc
KDM: lysine demethylase
KEGG: Kyoto Encyclopedia of Genes and Genomes
Ki67: MKI67
KMT: lysine methyltransferase
LDHA: lactate dehydrogenase A
MRI: magnetic resonance imaging
NP: nucleus pulposus
NPCs: nucleus pulposus cells
OEA: Oleoylethanolamide
PCA: Principal component analysis
PDK1: pyruvate dehydrogenase kinase 1
PPARα: peroxisome proliferator–activated receptor alpha
qPCR: quantitative polymerase chain reaction
RNPCs: rat nucleus pulposus cells
SA‐β‐Gal: Senescence‑associated β‑galactosidase
SD: standard deviation
SETD1A: SET domain–containing 1A
S‐FG: Safranin O‐fast green
TGF‐β1: transforming growth factor‐beta 1
TSS: transcription start site
WT: wild‐type
γH2AX: phosphorylated H2A histone family member X

## 1 Introduction

Intervertebral disc degeneration (IDD) is a common age‐related disorder primarily affecting middle‐aged and older [1, 2]. It is characterized by the progressive deterioration of intervertebral disc (IVD) structure and function and represents a leading cause of lower back pain, substantially impairing quality of life [3, 4]. With the global population aging, the incidence of IDD continues to rise, posing an increasingly significant public health challenge [5]. Nucleus pulposus cells (NPCs) are essential for maintaining IVD homeostasis. During aging, cellular senescence disrupts this balance, as senescent NPCs undergo irreversible cell cycle arrest and adopt a senescence‐associated secretory phenotype that exacerbates disc matrix degradation and promotes IDD progression [6, 7]. A further understanding of the molecular events driving NPCs senescence and IDD process may yield new therapeutic strategies to slow or prevent disease progression.

Epigenetic regulation, particularly histone modifications, has recently emerged as a critical contributor to degenerative diseases [1, 8, 9, 10]. Gaocai Li et al. reported that protein methylation represents a key epigenetic mechanism underlying IDD. It occurs primarily at lysine residues 4, 9, and 27 of histone H3 (H3K4, H3K9, H3K27). Catalyzed by specific methyltransferases and demethylases, this dynamically reversible modification modulates target gene expression and is directly associated with IDD [11]. Trimethylation of histone H3 at lysine 4 (H3K4me3) is a transcriptionally activating epigenetic mark that modulates gene expression by altering chromatin structure, recruiting transcriptional cofactors, and facilitating RNA polymerase II activity [12]. Dysregulation of H3K4me3 at promoter regions has been closely linked to cellular senescence [13, 14, 15]. SET domain‐containing 1A (SETD1A), the principal methyltransferase responsible for H3K4me3, regulates chromatin accessibility and gene transcription through this modification [16]. Previous studies have suggested that SETD1A exerts protective effects against cellular senescence [17], yet its role, as well as that of H3K4me3, in NPCs senescence and IDD remains unclear.

In this study, we investigated changes in H3K4me3 during IDD, examined its regulatory effects in NPCs, and identified SETD1A as a key upstream modulator of this process. We further validated the role of SETD1A in NPCs senescence and IDD progression and elucidated the underlying molecular mechanism. These findings provide new insights into the epigenetic regulation of NPCs function and suggest that targeting the SETD1A–H3K4me3 axis may offer a promising strategy to delay IDD.

## 2 Results

### 2.1 Reduced H3K4me3 Levels are Associated with NPCs Senescence

**FIGURE 1:**
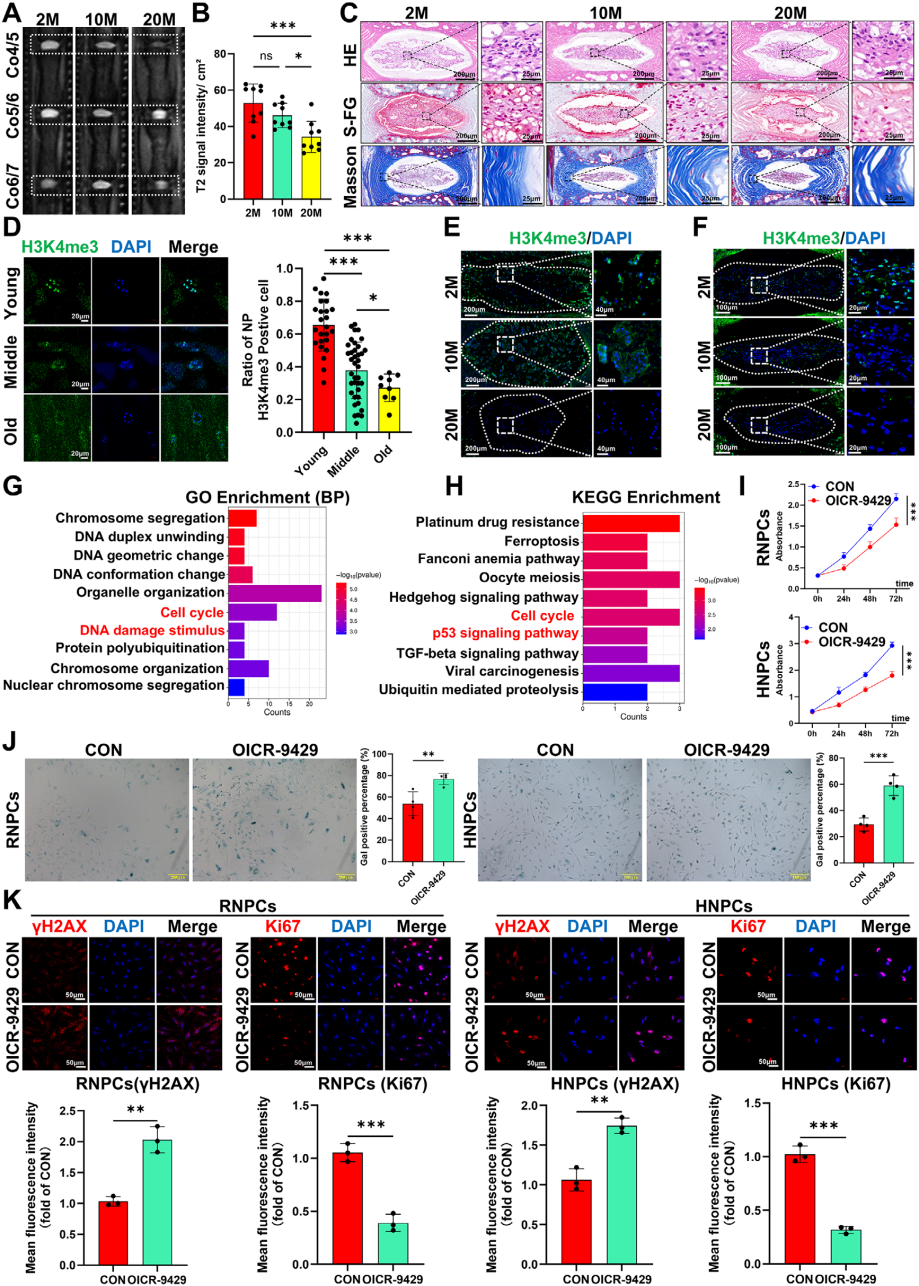
H3K4me3 expression of NP tissues decreases in degenerative IVDs, and its loss induces NPC senescence. (A,B) MRI of mouse caudal IVDs at different ages, showing representative T2‐weighted images and quantification of T2 signal intensity (*n* = 9; 3 IVDs analyzed per rat, 9 IVDs total). (C) H&E, S‐FG, and Masson's trichrome staining of mouse caudal IVDs at various ages. (D) Representative immunofluorescence staining of H3K4me3 in human lumbar NP tissues from different age groups. Quantification of H3K4me3‐positive NPCs is shown. Young (<36 years old, *n* = 25), Middle (36–64 years old, *n* = 38), and Old (>64 years old, *n* = 9). Immunofluorescence staining of H3K4me3 in rat (E) and mouse (F) caudal NP tissues at different ages. (G, H) GO and KEGG enrichment analyses of DEGs in RNPCs treated with OICR‐9429 or DMSO. (I) Cell viability measured by CCK‐8 assay (*n* = 3). (J) SA‐β‐Gal staining and quantification of SA‐β‐Gal–positive NPCs (*n* = 4). (K) Representative immunofluorescence staining of γH2AX and Ki67 in NPCs treated with indicated agents and quantitative analysis (*n* = 3). ^*^ *p* < 0.05, ^**^ *p *< 0.01, ^***^ *p *< 0.001, ns = not significant. (Unpaired *t*‐test; Welch ANOVA; one‐way ANOVA; two‐way ANOVA).

Aging‐associated IDD models in humans and rodents are well established [18]. In this study, we analyzed medical records and lumbar Magnetic resonance imaging (MRI) data from 121 patients. Based on age stratification and the latest classification criteria [19], MRI and Pfirrmann grading showed that the degeneration of human L1–S1 IVDs progressively increased with age (Figure S1A,B). Similarly, C57BL/6J mice were divided into three groups (2, 10, and 20 months) [20]. MRI and micro‐computed tomography(µCT) revealed an age‐dependent reduction in IVD height and T2 signal intensity (Figure 1A,B; Figure S1C). Histopathological analyses using hematoxylin–eosin (H&E), Safranin O‐fast green(S‐FG), and Masson's trichrome staining confirmed progressive structural deterioration of the caudal IVDs, consistent with increased histological degeneration scores (Figure 1C; Figure S1D). Collectively, these findings indicate that mouse IVDs undergo degenerative changes with aging.

To investigate the role of H3K4me3 in this process, we assessed its expression across human, rat, and mouse NP tissues. Immunofluorescence analyses showed a marked decrease in H3K4me3 levels in degenerative NP tissues (Figure 1D–F; Figure S1E,F). Chromatin immunoprecipitation sequencing (ChIP‐seq) of NP tissues from 2 and 20‐month‐old rats revealed distinct genomic H3K4me3 enrichment profiles. Differentially enriched peaks were associated with genes involved in cell cycle pathways, as shown by GO and KEGG analyses (Figure S1G–I). Overlap analysis of mRNA‐seq and ChIP‐seq of NP tissue from rats with different ages revealed marked downregulation of cyclin D2(CCND2), a core cell cycle regulator, in degenerative NP tissues (Figure S1J), suggesting that H3K4me3 depletion contributes to the transcriptional repression of genes related to cycle regulation of NPCs. Rat NP cells (RNPCs) were treated with OICR‐9429, and the H3K4me3 protein expression level was significantly decreased in RNPCs (Figure S1K). GO and KEGG enrichment analyses of mRNA‐seq data revealed significant alterations in cell cycle, DNA damage response, and p53 signaling pathways following H3K4me3 inhibition (Figure 1G,H). Human NP cells (HNPCs) (validated phenotypically, Figure S1L,M) were treated with OICR‐9429. CCK‐8 assays revealed a significant reduction in cell proliferation (Figure 1I), while Senescence‑associated β‑galactosidase (SA‐β‐Gal) staining demonstrated increased cellular senescence (Figure 1J). Furthermore, phosphorylated H2A histone family member X(γH2AX) and MKI67(Ki67) immunofluorescence staining confirmed enhanced DNA damage and reduced proliferative activity in H3K4me3‐depleted cells (Figure 1K). Collectively, these results demonstrate that decreased H3K4me3 levels lead to cell cycle arrest, impaired proliferation, and accelerated senescence in NPCs, implicating H3K4me3 loss as a critical epigenetic mechanism driving IDD progression.

### 2.2 Downregulation of SETD1A Promotes NPCs Senescence and IDD

**FIGURE 2:**
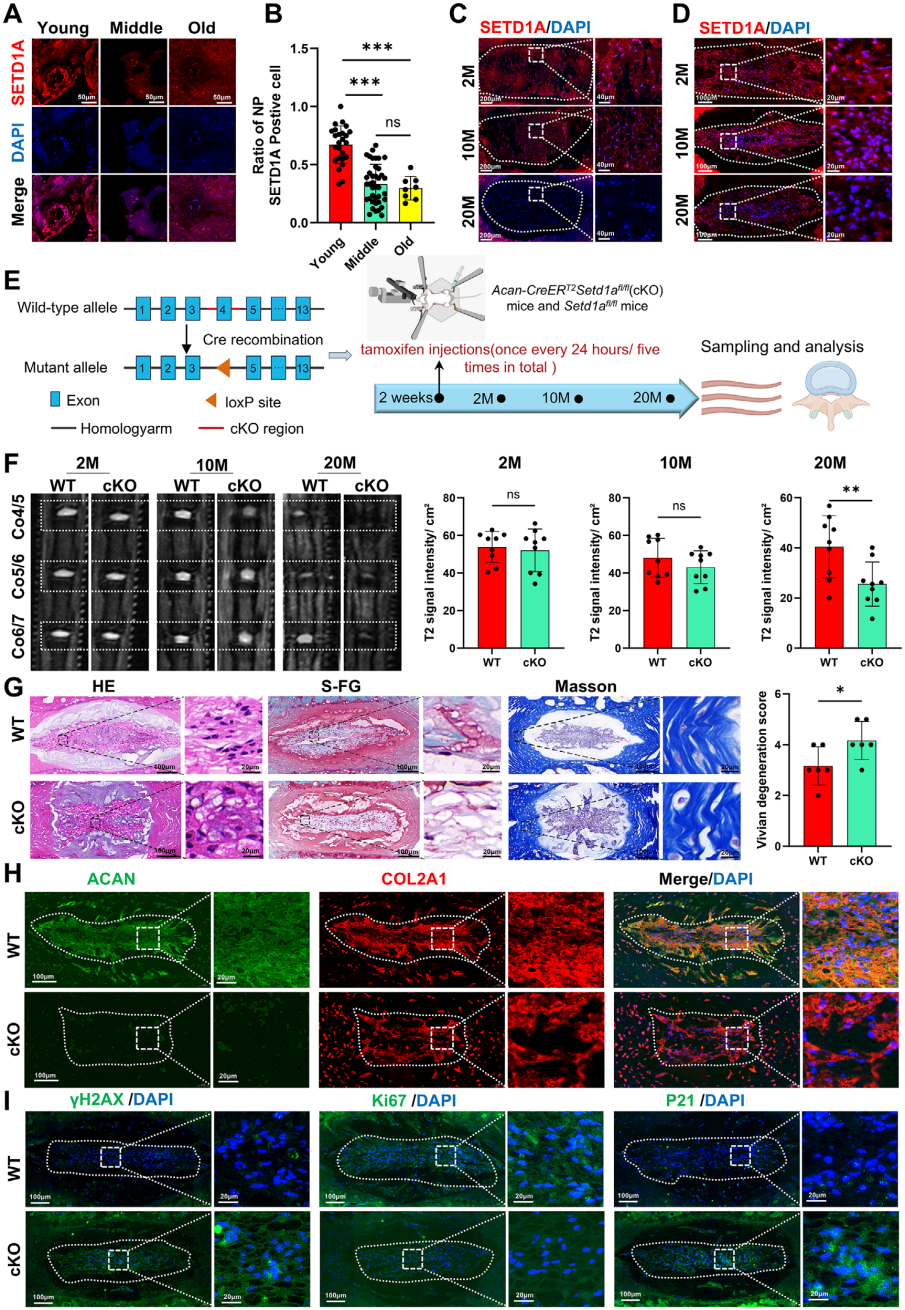
Downregulation of SETD1A accelerates IDD. (A, B) Representative immunofluorescence staining of SETD1A in human lumbar NP tissue across age groups: Youth (*n* = 25), Middle (*n* = 39), and Old (*n* = 8). Quantification of SETD1A‐positive NPCs is shown. Representative immunofluorescence staining of SETD1A in caudal NP tissue of rats (C) and mice (D) at different ages. (E) Schematic illustration of SETD1A‐cKO mouse generation. (F) MRI of caudal IVDs in WT and cKO mice of various ages with quantification of T2 signal intensity (*n* = 9; 3 IVDs per mouse, 3 mice total). (G) H&E, S‐FG, and Masson's trichrome staining of caudal IVDs from 20‐month‐old mice. Histological scoring of degeneration (*n* = 6; 2 IVDs per mouse, 3 mice total). (H, I) Representative immunofluorescence staining of indicated markers in caudal NP tissue from cKO and WT mice at different ages. ^*^ *p* < 0.05, ^**^ *p* < 0.01, ^***^ *p* < 0.001, ns = not significant. (Unpaired *t*‐test; one‐way ANOVA).

H3K4me3 is dynamically regulated by histone methyltransferases (Histone lysine N‐methyltransferase 2A‐D, SETD1A/B) and demethylases (lysine demethylase 1A/B, lysine demethylase 5A‐D) [21]. Analysis of mRNA‐seq data from rat NP tissues of 2‐ and 20‐month‐old rats (GEO accession: GSE234369) [22], revealed that SETD1A expression exhibited an age‐related decline in NP tissues (Figure 2A), consistent with the reduction of H3K4me3 levels shown in previous studies. qPCR further validated this trend (Figure S2B), suggesting a close association between SETD1A and H3K4me3 expression. Immunofluorescence analyses confirmed that SETD1A expression was markedly decreased in degenerative NP tissues from humans, rats, and mice (Figure 2A–D; Figure S2C, D), indicating that SETD1A downregulation is correlated with IDD. To explore this relationship in vivo, we generated Acan‐CreER^T2^; SETD1A^fl/fl^ conditional knockout (SETD1A‐cKO, hereinafter referred to as cKO) mice (Figure 2E), in which both SETD1A and H3K4me3 levels were significantly reduced (Figure S2E,F). MRI revealed that 20‐month‐old SETD1A‐cKO mice exhibited reduced T_2_ signal intensity and disc height in caudal NP tissues compared with age‐matched wild‐type (WT) mice (Figure 2F; Figure S2G). Histological evaluation showed that Vivian's degeneration scores of caudal IVDs were significantly higher in cKO mice (Figure 2G), consistent with reduced extracellular matrix (ECM) content and increased fibrosis. Immunostaining demonstrated reduced expression of ACAN, Collagen II (COL2A1), Ki67, and CCND2, alongside elevated expression of γH2AX, p21, transforming growth factor‐beta 1 (TGF‐β1), and Collagen I (COL1A1) in cKO mice NP tissues (Figure 2H,I; Figure S2H,I). These changes indicate that SETD1A deficiency compromises ECM synthesis, accelerates NPCs senescence, and promotes NP tissues' fibrotic remodeling, collectively aggravating IDD progression.

**FIGURE 3:**
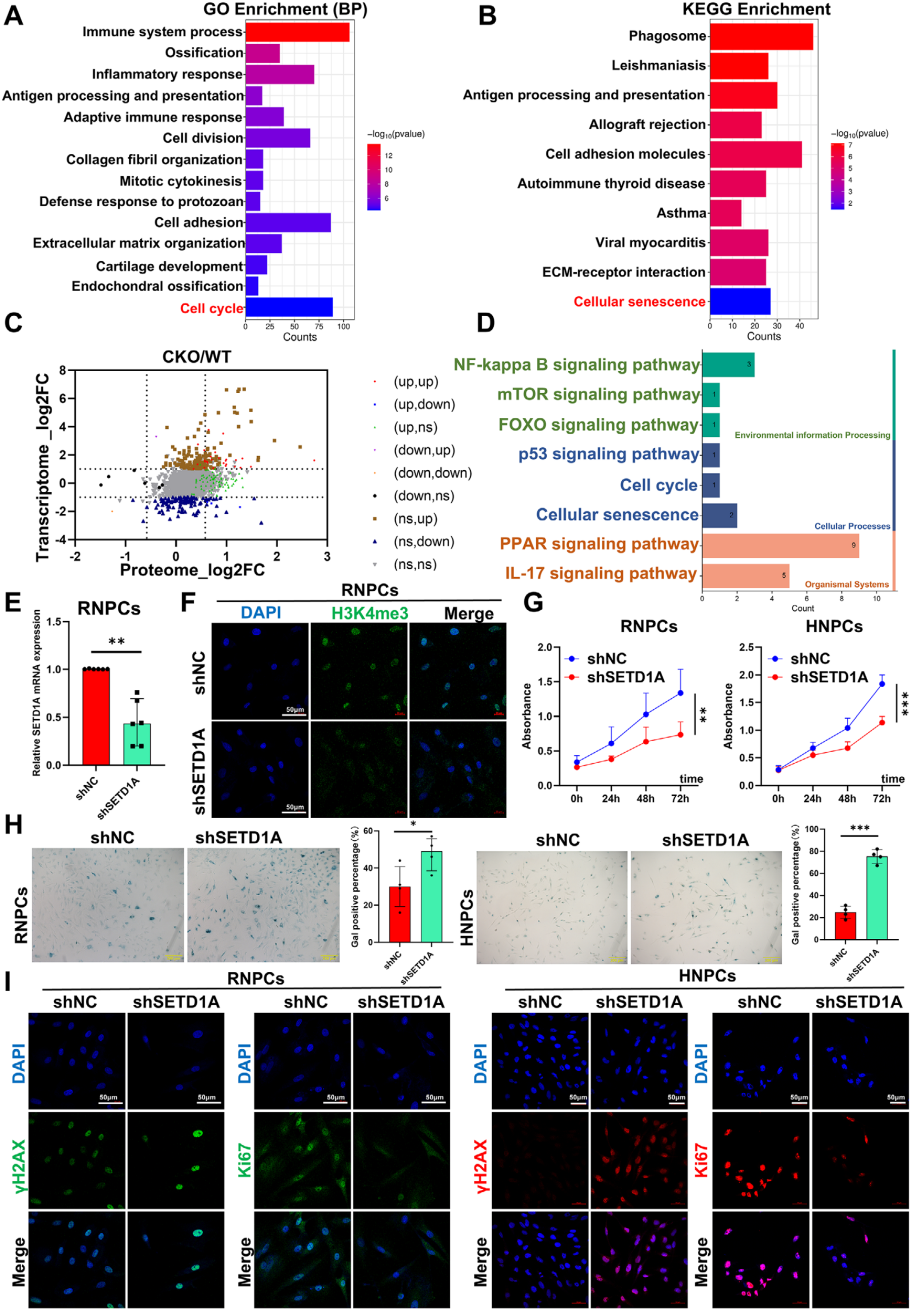
Downregulation of SETD1A induces senescence of NPCs. (A,B) GO and KEGG enrichment analyses of DEGs from WT and cKO mouse caudal IVDs at 20 months. (C) Nine‐quadrant scatter plot of differentially expressed proteins in WT versus cKO groups. (D) KEGG enrichment analyses of DEGs from integrated transcriptome and proteome analyses of WT and cKO mice. (E) mRNA expression of SETD1A measured by qPCR in RNPCs transfected with shNC or shSETD1A (*n =* 6). (F) Representative immunofluorescence staining of H3K4me3 in RNPCs treated with indicated agents. (G) Cell viability by CCK‐8 assay (*n =* 3). (H) SA‐β‐Gal staining and quantification of SA‐β‐Gal–positive NPCs (*n =* 4). (I) Representative immunofluorescence staining of γH2AX and Ki67 in NPCs treated with indicated agents. ^*^ *p* < 0.05, ^**^ *p* < 0.01, ^***^ *p* < 0.001, ns = not significant. (Unpaired *t*‐test; Mann–Whitney U test; two‐way ANOVA).

To further elucidate the mechanism by which SETD1A regulates IDD, we performed mRNA‐seq and proteomic profiling of caudal IVDs from WT and cKO mice. Principal component analysis (PCA) confirmed high sample reproducibility, while the volcano plot and heatmaps highlighted substantial changes in gene and protein expression following SETD1A knockout (Figure S3A–F). GO and KEGG enrichment analyses revealed that transcriptomic alterations were significantly associated with cell cycle and cellular senescence pathways (Figure 3A,B), proteomics analysis revealed significant enrichment of the inflammatory response and ECM‑receptor interaction pathways (Figure S3G,H). Overlap analysis of the two datasets identified overlapping differentially expressed genes (DEGs) enriched in the same pathways (Figure 3C,D). To validate these omics findings, SETD1A was knockdown in both RNPCs and HNPCs via lentiviral transfection, as confirmed by qPCR (Figure 3E; Figure S3I). Knockdown of SETD1A markedly reduced H3K4me3 expression (Figure 3F; Figure S3J), decreased cellular proliferation (Figure 3G), increased the proportion of SA‐β‐Gal‐positive cells (Figure 3H), and resulted in reduced Ki67 protein expression and elevated γH2AX protein levels in NPCs (Figure 3I; Figure S3K–M). Collectively, these results demonstrate that SETD1A deficiency leads to reduced H3K4me3 expression levels, induction of NPCs senescence, and exacerbation of IDD, underscoring the critical epigenetic role of SETD1A in maintaining disc homeostasis.

### 2.3 Overexpression of SETD1A Attenuates NPCs Senescence and IDD

**FIGURE 4:**
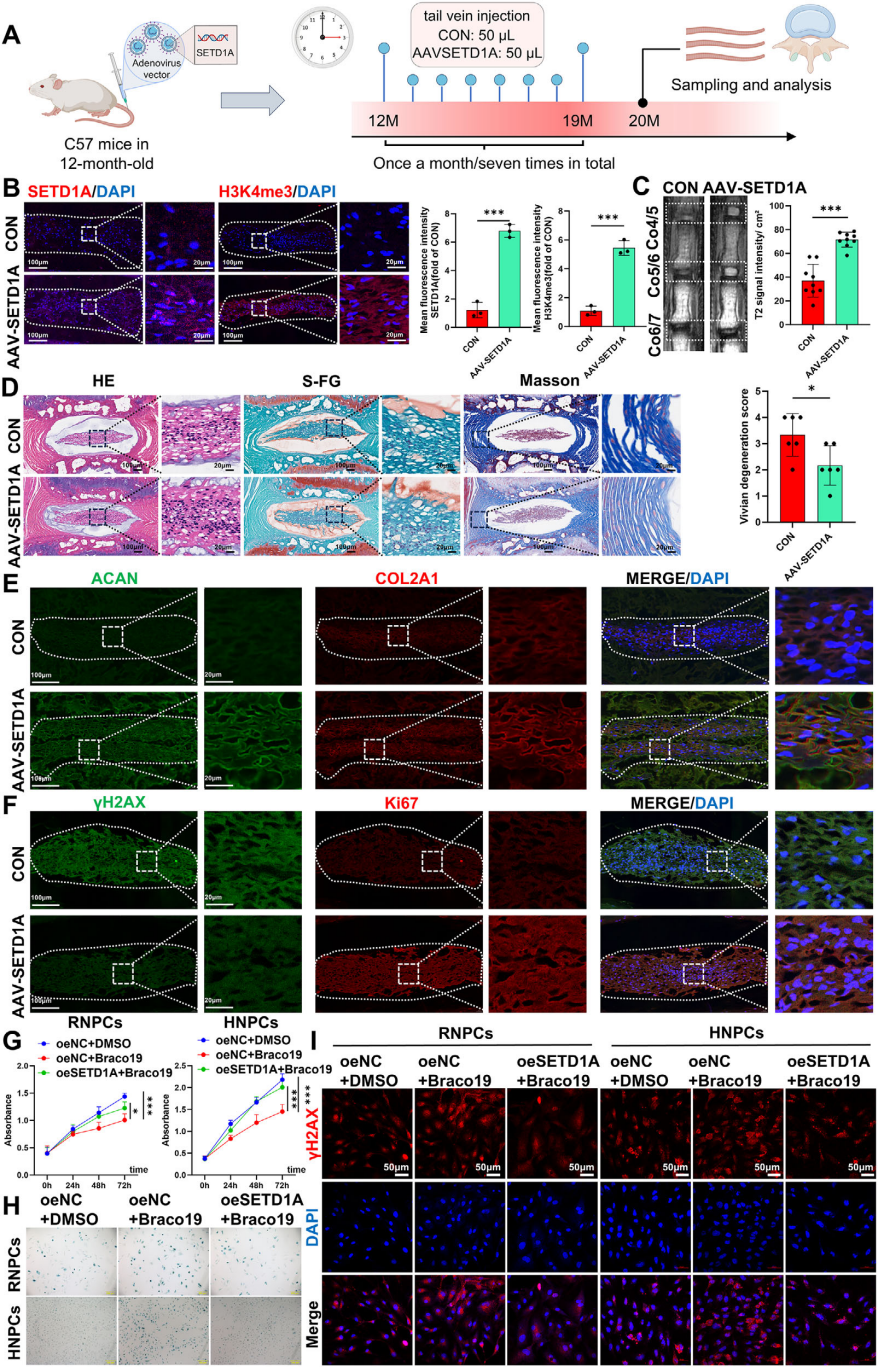
Overexpression of SETD1A delays IDD and NPC senescence. (A) Experimental protocol for SETD1A overexpression in mice. (B) Representative immunofluorescence staining of SETD1A and H3K4me3 in caudal IVD sections from different groups, with quantification of mean fluorescence intensity (*n* = 3). (C) MRI of caudal IVDs showing T2‐weighted signal intensity in each group (*n* = 9; 3 IVDs per mouse, 3 mice total). (D) H&E, S‐FG, and Masson's trichrome staining of caudal IVDs from 20‐month‐old mice, with Vivian histological scores (*n* = 6; 2 IVDs per mouse, 3 mice total). (E,F) Representative immunofluorescence staining of indicated markers in caudal IVDs from mice of different treatment conditions. (G) Cell proliferation analyzed by CCK‐8 assay (*n* = 3). (H) SA‐β‐Gal staining of NPCs. (I) Representative immunofluorescence staining of γH2AX in NPCs treated with indicated agents. ^*^ *p* < 0.05, ^**^ *p* < 0.01, ^***^ *p* < 0.001, ns = not significant. (Unpaired *t*‐test; Welch ANOVA; two‐way ANOVA).

To further evaluate the protective role of SETD1A on IDD, we overexpressed SETD1A in vivo via adenoviral tail vein injection (Figure 4A), Live bioluminescence imaging and immunofluorescence analyses of tail IVDs confirmed efficient transgene delivery and upregulation of SETD1A and H3K4me3 expression (Figure 4B; Figure S4A). MRI of caudal IVDs in 20‐month‐old mice demonstrated that SETD1A overexpression restored T2 signal intensity and improved disc morphology, while µCT showed increased disc height compared with controls (Figure 4C; Figure S4B). Histological staining with H&E, S‐FG, Masson's trichrome revealed a lower Vivian's degeneration score, enhanced ECM integrity, and reduced fibrosis in the Adeno‐associated virus (AAV) ‐SETD1A group (Figure 4D). At the molecular level, SETD1A overexpression significantly increased the expression of ACAN and COL2A1, while decreasing γH2AX and p21 levels in NP tissues (Figure 4E,F; Figure S4C–E). These findings indicate that SETD1A overexpression preserves ECM homeostasis and mitigates cellular senescence within NP tissues. To further confirm the anti‐senescent effect of SETD1A, we employed BRACO‐19, a telomerase inhibitor known to induce senescence. BRACO‐19 treatment reduced NPCs proliferation, whereas SETD1A overexpression partially rescued proliferative capacity (Figure 4G). Moreover, SETD1A overexpression significantly decreased SA‐β‐Gal positivity and γH2AX accumulation in BRACO‐19–treated NPCs (Figure 4H,I; Figure S4F,G). Collectively, these results demonstrate that SETD1A overexpression suppresses NPC senescence, enhances ECM synthesis, and alleviates IDD.

### 2.4 Hypoxia‐Inducible Factor 1‐Alpha (HIF1α) Mediates the Regulatory Effects of SETD1A on Glycolysis and Senescence in NPCs

**FIGURE 5:**
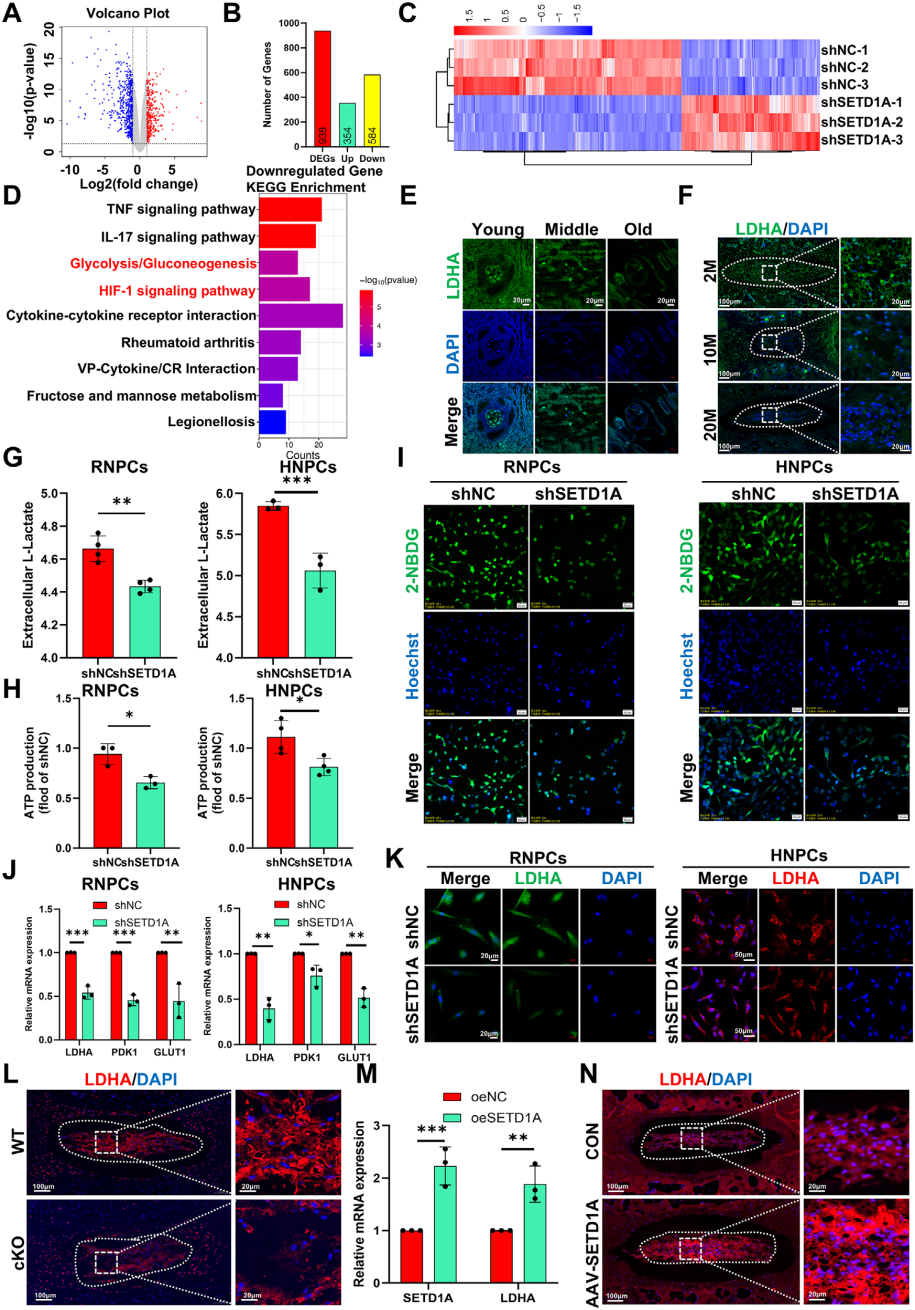
SETD1A regulates glycolysis as a key determinant of NPC senescence. (A) Volcano plot of DEGs between shNC and shSETD1A RNPCs based on mRNA‐seq (*n* = 3). Fold‐change (x‐axis) is plotted against statistical significance (y‐axis); upregulated genes (red) and downregulated genes (blue) were defined by fold change ≥ 2 and *p* < 0.05. (B) Number of DEGs between the shNC and shSETD1A RNPCs. (C) Heatmap of DEGs in shSETD1A versus shNC RNPCs. (D) KEGG analysis of downregulated DEGs in shSETD1A RNPCs. (E) Representative immunofluorescence staining of LDHA in human lumbar NP tissues of different ages. (F) Representative immunofluorescence staining of LDHA in caudal NP tissue from mouse at different ages. (G) Extracellular lactate levels in RNPCs (*n* = 4) and HNPCs (*n* = 3). (H) Intracellular ATP content in RNPCs (*n* = 4) and HNPCs (*n* = 3). (I) Glucose uptake capacity in NPCs from indicated groups. (J) mRNA expression of LDHA, PDK1, and GLUT1 detected by qPCR (*n* = 3). (K) Representative immunofluorescence staining of LDHA in RNPCs and HNPCs treated with indicated agents. (L) Representative immunofluorescence staining of LDHA in mouse caudal IVDs from different groups. (M) mRNA expression of LDHA, PDK1, and GLUT1 in RNPCs after SETD1A overexpression, determined by qPCR (*n* = 3). (N) Representative immunofluorescence staining of LDHA in mouse caudal IVDs after SETD1A overexpression. ^*^ *p* < 0.05, ^**^ *p* < 0.01, ^***^ *p* < 0.001, ns = not significant. (Unpaired *t*‐test).

To investigate how SETD1A influences NPC senescence, mRNA‐seq was performed following SETD1A knockdown (Figure 5A–C). KEGG pathway analysis revealed significant enrichment of DEGs in glycolysis and HIF‐1 signaling pathway (Figure 5D). Notably, the expression of lactate dehydrogenase A (LDHA), a key glycolytic enzyme, was markedly reduced in IDD tissues of both humans and mice (Figure 5E,F; Figure S5A,B). Given the unique avascular microenvironment of IVDs, glycolysis is the principal energy source for NPCs even under normoxic conditions [23, 24, 25]. Glycolytic dysfunction has been implicated in promoting NPC senescence [26, 27]. Accordingly, we hypothesized that SETD1A may regulate NPC senescence by modulating glycolytic activity. Consistent with this, SETD1A knockdown led to pronounced reduction in extracellular lactate, intracellular ATP, and glucose uptake in NPCs (Figure 5G–I; Figure S5C). The expression of LDHA was significantly downregulated at both mRNA and protein levels following SETD1A suppression (Figure 5J,K; Figure S5D), accompanied by transcription of other glycolytic regulators, including pyruvate dehydrogenase kinase 1 (PDK1) and glucose transporter 1 (GLUT1) (Figure 5J). Likewise, LDHA expression was reduced in the IVDs of SETD1A‐cKO mice (Figure 5L; Figure S5E). In contrast, SETD1A overexpression increased the expression of LDHA, PDK1, and GLUT1 in cultured NPCs and elevated LDHA expression in vivo (Figure 5M,N; Figure S5F). Together, these findings identify SETD1A as a crucial regulator of glycolytic metabolism in NPCs.

**FIGURE 6:**
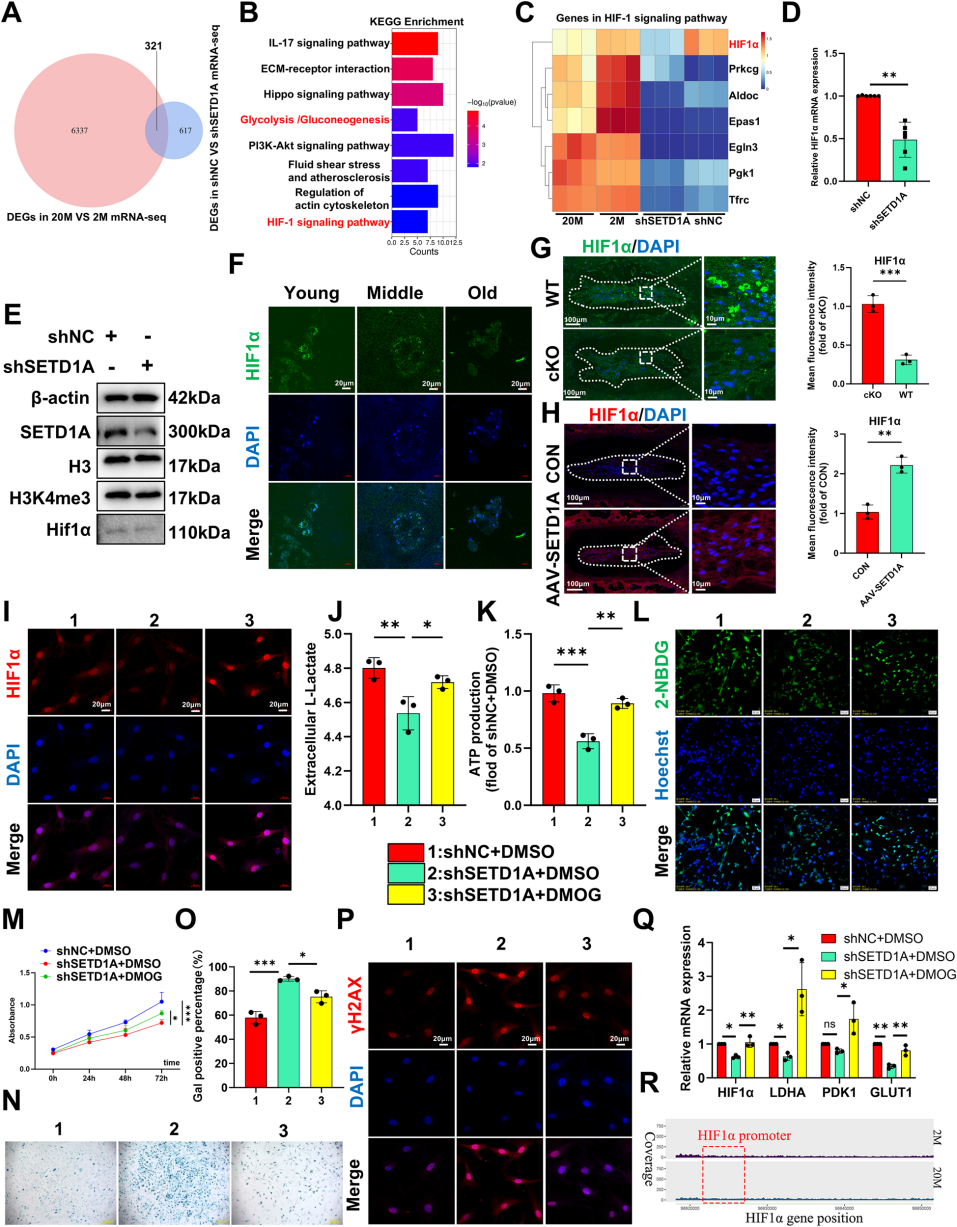
HIF1α mediates the regulatory effects of SETD1A on glycolysis and senescence of NPCs. (A) Overlap of DEGs between the NP tissues of rats at different ages and shSETD1A versus shNC RNPCs. (B) KEGG pathway enrichment analysis of overlapping DEGs in (A). (C) Heatmap of genes enriched in the HIF‐1 pathway. (D) mRNA expression of HIF1α detected by qPCR in RNPCs (*n* = 6). (E) Western blot analysis of RNPCs treated with the indicated agents. (F) Representative immunofluorescence staining of HIF1α in human NP tissue of different groups. (G, H) Representative immunofluorescence staining of HIF1α in caudal IVDs from mouse. (I) Representative immunofluorescence staining of HIF1α in RNPCs treated with different agents. (J) Extracellular lactate levels of RNPCs from indicated groups (*n* = 3). (K) Intracellular ATP content in RNPCs (*n* = 3). (L) Glucose uptake capacity of RNPCs from different groups. (M) Cell proliferation of RNPCs assessed by CCK‐8 assay (*n* = 3). (N, O) SA‐β‐Gal staining and quantification of SA‐β‐Gal–positive RNPCs (*n* = 3). (P) Representative immunofluorescence staining of γH2AX in RNPCs treated with different agents. (Q) mRNA expression of HIF1α, LDHA, PDK1, and GLUT1 in RNPCs measured by qPCR (*n* = 3). (R) H3K4me3 enrichment in HIF1α gene of NP tissue from 2‐month‐old versus 20‐month‐old rats. ^*^ *p* < 0.05, ^**^ *p* < 0.01, ^***^ *p* < 0.001, ns = not significant. (Mann–Whitney U test; one‐way ANOVA; two‐way ANOVA).

In mRNA‐seq of rat NP tissue, expression levels of 51 HIF‐1 signaling pathway‐related genes showed significant changes with aging (Figure S6A,B). HIF1α is both constitutively expressed in NPCs [28] and a master transcription factor controlling glycolytic metabolism [29, 30]. We next examined whether HIF1α mediates SETD1A‐driven glycolytic regulation. By integrating mRNA‐seq datasets from shSETD1A NPCs and age‐stratified rat NP tissues, we identified 321 overlapping genes (Figure 6A). KEGG enrichment analysis of these genes highlighted significant enrichment in the HIF‐1 signaling pathway (Figure 6B), with HIF1α showing the most substantial expression change (Figure 6C). SETD1A knockdown significantly reduced HIF1α expression in both RNPCs and HNPCs (Figure 6D,E; Figure S6C), and protein expression levels decreased were observed in aging human and mouse NP tissues (Figure 6F; Figure S6D, E). In mouse NP tissue, SETD1A positively regulates HIF1α expression levels. (Figure 6G,H). To confirm the functional role of HIF1α, we used dimethyloxalylglycine (DMOG) to stabilize HIF1α protein levels. DMOG treatment restored HIF1α expression suppressed by SETD1A knockdown in RNPCs (Figure 6I; Figure S6F), and alleviated the resulting senescent phenotype and glycolytic defects (Figure 6J–Q; Figure S6G–K). These findings establish HIF1α as a key mediator linking SETD1A to the regulation of glycolysis and NPC senescence.

Given that SETD1A functions as a histone H3K4me3 methyltransferase, we hypothesized that it might regulate HIF1α transcription by promoting H3K4me3 enrichment at the HIF1α promoter. Surprisingly, ChIP‐seq analysis of rat NP tissues revealed no detectable H3K4me3 peak at the HIF1α promoter (Figure 6R), suggesting that SETD1A does not directly target HIF1α and instead acts through an intermediate regulatory mechanism.

### 2.5 SETD1A Regulates HIF1α Expression via Epigenetic Activation of HELZ2 Transcription

**FIGURE 7:**
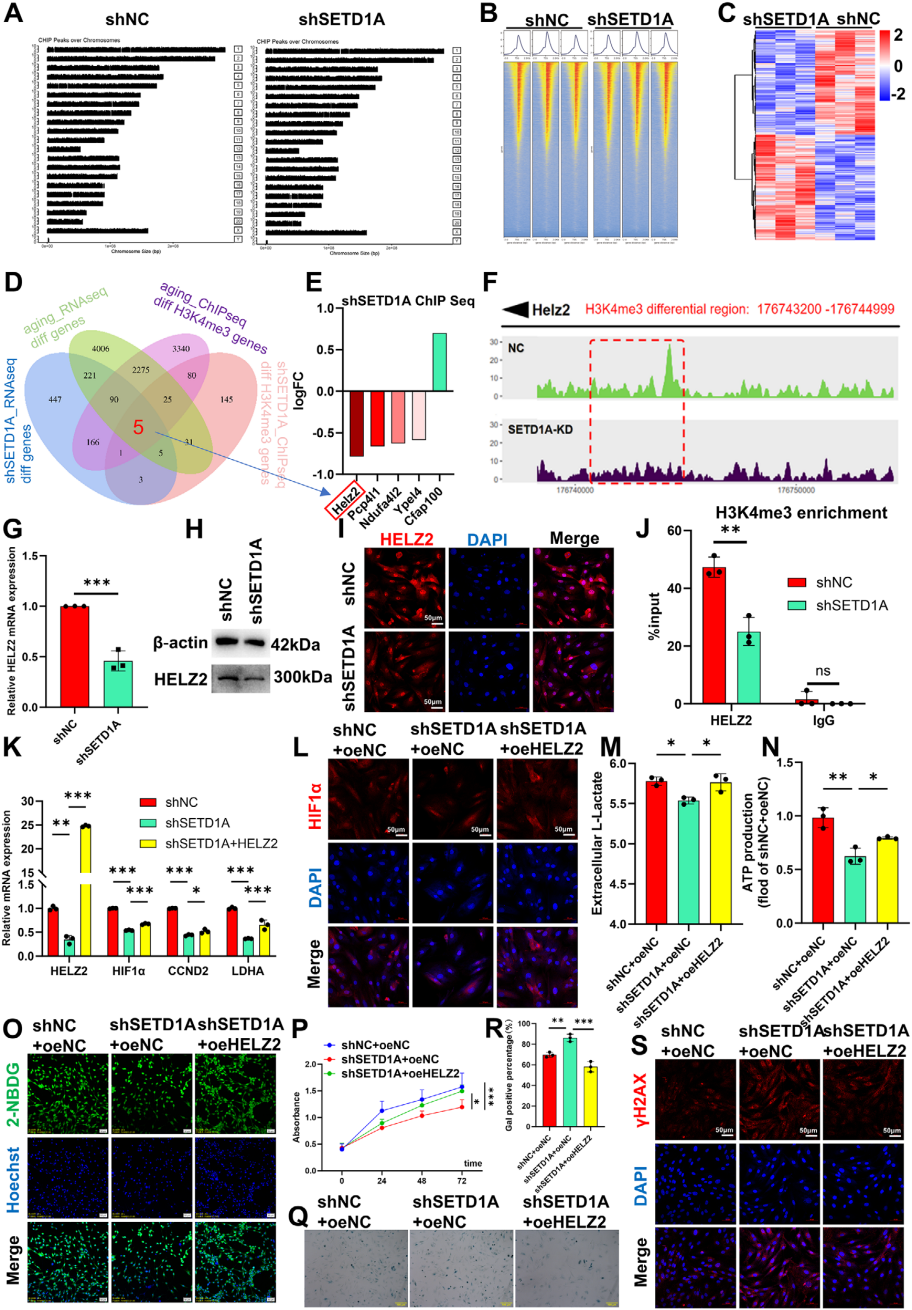
SETD1A regulates HIF1α expression and NPCs function through HELZ2. H3K4me3 ChIP‐seq was performed on RNPCs treated with shNC and shSETD1A. (A) Chromosomal distribution of H3K4me3 peaks. (B) Read distribution around transcription start sites (TSSs) in peak annotations. (C) Heatmap of differentially enriched peaks. (D) Overlap of ChIP‐seq and mRNA‐seq data (differential peaks screened from promoter regions). (E) Fold change in H3K4me3 levels within promoter regions. (F) H3K4me3 enrichment in HELZ2 gene of RNPCs. (G) mRNA expression of HELZ2 in RNPCs measured by qPCR (*n* = 3). (H) Western blot analysis of HELZ2 protein in RNPCs treated with indicated agents. (I) Representative immunofluorescence staining of HELZ2 in RNPCs treated with different agents. (J) ChIP‐qPCR analysis of H3K4me3 enrichment at the HELZ2 promoter in RNPCs (shNC vs. shSETD1A); rabbit IgG served as IP control (*n* = 3). (K) mRNA expression of HELZ2, HIF1α, CCND2, and LDHA in RNPCs detected by qPCR (*n* = 3). (L) Representative immunofluorescence staining of HIF1α in RNPCs treated with indicated agents. (M) Extracellular lactate levels in RNPCs from different groups (*n* = 3). (N) Intracellular ATP content in RNPCs (*n* = 4). (O) Glucose uptake capacity in RNPCs from different groups. (P) RNPCs proliferation assessed by CCK‐8 assay (*n* = 3). (Q, R) SA‐β‐Gal staining and quantification of SA‐β‐Gal–positive RNPCs. (S) Representative immunofluorescence staining of γH2AX in RNPCs treated with different agents. ^*^ *p* < 0.05, ^**^ *p* < 0.01, ^***^ *p* < 0.001, ns = not significant. (Unpaired t‐test; Mann–Whitney U test; one‐way ANOVA; two‐way ANOVA).

To elucidate how SETD1A regulates HIF1α expression in NPCs, ChIP‐Seq for H3K4me3 was performed by in RNPCs. The shNC and shSETD1A groups exhibited 70 340 and 70 427 peaks, respectively (Figure S7A). Chromosomal and transcription start site (TSS) peak distribution are shown in Figure 7A,B. In total, 20 010 genes were annotated in the shNC group and 20 423 in the shSETD1A group (Figure S7B), with 4768 differential peaks identified (Figure S7C). Peak lengths clustered around 1600 bp (Figure S7D), and most differential peaks were in intergenic regions, with only 6.5% in promoter regions (Figure S7E). A heatmap of differential H3K4me3 peaks is presented in Figure 7C. GO enrichment of differential H3K4me3 peak‐associated genes indicated a strong association with cell cycle and glucose metabolic process (Figure S7F). Integrative analysis combining these ChIP‐seq results with mRNA‐seq data from both aged rat NP tissues and SETD1A knockdown RNPCs identified five overlapping genes meeting the selection criteria (Figure 7D). Among these, the HELZ2 promoter exhibited the most pronounced decrease in H3K4me3 enrichment following SETD1A knockdown (Figure 7E,F). Since HELZ2 is known to function as a transcriptional coactivator [31, 32], it was selected for further investigation. SETD1A knockdown markedly reduced HELZ2 mRNA and protein levels in RNPCs (Figure 7G–I; Figure S7G), consistent with a loss of H3K4me3 at the HELZ2 promoter (Figure 7J). These findings confirm that SETD1A directly promotes HELZ2 transcription via H3K4me3 deposition at its promoter region. Functionally, HELZ2 knockdown significantly decreased HIF1α expression in RNPCs (Figure S7H,I), suppressed glycolysis (Figure S7J–L), and induced cellular senescence (Figure S7M–O). Conversely, HELZ2 overexpression in SETD1A‐deficient RNPCs restored HIF1α expression (Figure 7K,L; Figure S7P), rescued glycolytic activity (Figure 7M–O; Figure S7Q), and reduced senescence markers (Figure 7P–S; Figure S7R). Together, these results demonstrate that SETD1A epigenetically activates HELZ2 transcription through promoter H3K4me3 enrichment, and HELZ2 acts as a critical intermediary linking SETD1A to HIF1α expression, thereby sustaining glycolytic metabolism and preventing NPCs senescence.

### 2.6 PPARα is Involved in the Regulation of HIF1α Expression by the SETD1A–HELZ2 Pathway in NPCs

**FIGURE 8:**
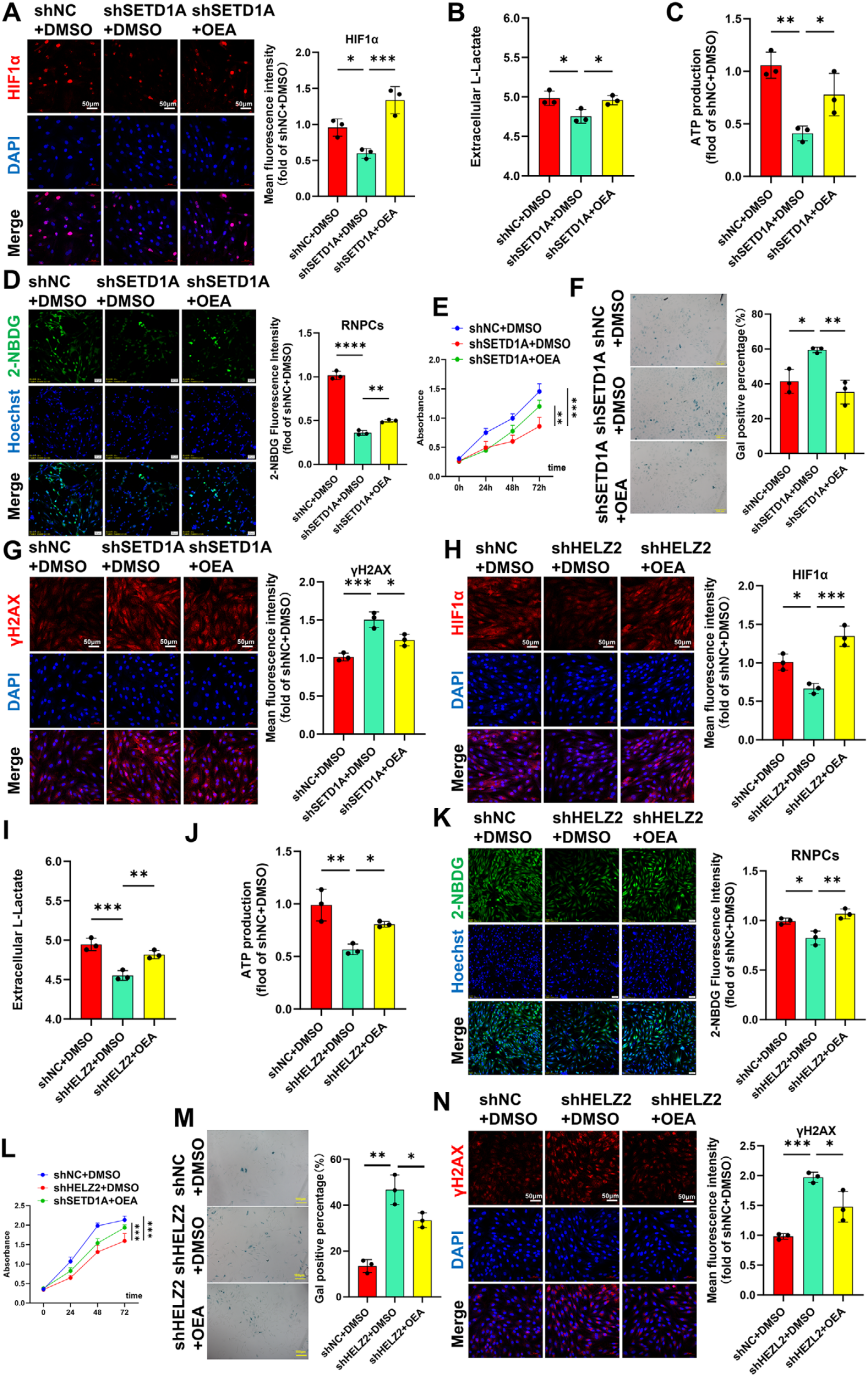
PPARα mediates the regulatory effects of SETD1A and HELZ2 on HIF1α and NPC function. (A) Representative immunofluorescence staining of HIF1α in RNPCs after SETD1A knockdown and PPARα agonist treatment. Quantification of fluorescence intensity is shown (*n* = 3). (B) Extracellular lactate levels in RNPCs from different groups (*n* = 3). (C) Intracellular ATP content in RNPCs (*n* = 3). (D) Glucose uptake capacity in RNPCs and quantification analysis(*n* = 3). (E) RNPC proliferation analyzed by CCK‐8 assay (*n* = 3). (F) SA‐β‐Gal staining and quantification of SA‐β‐Gal–positive RNPCs (*n* = 3). (G) Representative immunofluorescence staining of γH2AX in RNPCs treated with different agents and quantitative analysis (*n* = 3). (H) Representative immunofluorescence staining of HIF1α in RNPCs after HELZ2 knockdown and PPARα agonist treatment. Quantification of fluorescence intensity is shown (*n* = 3). (I) Extracellular lactate levels in RNPCs from different treatment groups (*n* = 3). (J) Intracellular ATP content in RNPCs (*n* = 3). (K) Glucose uptake capacity in RNPCs from indicated groups and quantification analysis(*n* = 3). (L) RNPC proliferation determined by CCK‐8 assay (*n* = 3). (M) SA‐β‐Gal staining and percentage of senescent RNPCs in each group (*n* = 3). (N) Representative immunofluorescence staining of γH2AX in RNPCs treated with indicated agents and quantitative analysis (*n* = 3). ^*^ *p* < 0.05, ^**^ *p *< 0.01, ^***^ *p *< 0.001, ns = not significant. (One‐way ANOVA; one‐way ANOVA; two‐way ANOVA).

Previous studies have reported that HELZ2 can form a transcriptional coactivator complex with PPARα [32, 33]. Notably, PPARα has also been identified as a transcription regulator of HIF1α [34]. To verify this interaction, we used JASPAR (https://jaspar.elixir.no/) to predict transcription factors binding to the human HIF1α promoter. PPARα exhibited strong binding affinity, and its predicted binding motif is shown in Figure S8A,B. These observations suggested that PPARα may participate in the SETD1A–HELZ2‐mediated regulation of HIF1α in NPCs. To test this hypothesis, we employed GW6471, a selective PPARα antagonist. GW6471 treatment significantly reduced HIF1α expression in RNPCs (Figure S8C), impaired glycolytic function (Figure S8D–F), and induced cellular senescence (Figure S8G–J). Conversely, treatment with OEA, a PPARα agonist, restored HIF1α expression in shSETD1A RNPCs (Figure 8A). OEA also rescued the associated glycolytic dysfunction (Figure 8B–D), and alleviated cellular senescence (Figure 8E–G; Figure S8K). Similar effects were observed in shHEZL2 RNPCs. OEA treatment reversed the decrease in HIF1α expression (Figure 8H), improved glycolytic activity (Figure 8I–K), and reduced senescence markers (Figure 8L–N; Figure S8L). Collectively, these findings demonstrate indicate that PPARα functions downstream of the SETD1A–HELZ2 axis and serves as a key transcriptional mediator linking SETD1A activity to HIF1α expression, thereby maintaining glycolytic metabolism and delaying senescence in NPCs.

## 3 Discussion

In this study, we demonstrated that H3K4me3 is markedly decreased in degenerative NP tissues and that this reduction promotes senescence of NPCs. SETD1A is a key methyltransferase responsible for maintaining H3K4me3 levels in NPCs. Conditional knockout of SETD1A in mice exacerbated IDD, while SETD1A overexpression protected against age‐related structural and cellular deterioration of IVDs. Mechanistically, SETD1A regulates HIF1α expression via the HELZ2–PPARα axis, and HIF1α mediates the effects of SETD1A on glycolytic metabolism and NPC senescence. Collectively, our findings establish a SETD1A–HELZ2–PPARα–HIF1α regulatory pathway that maintains glycolytic function and delays NPC senescence, providing a theoretical foundation for targeted epigenetic interventions in IDD.

Epigenetics regulation, including histone modifications, DNA methylation, and non‐coding RNA‐mediated control, dynamically modulates gene expression without altering the underlying DNA sequence. Aberrant epigenetic modifications can disrupt cellular homeostasis and contribute to degenerative and metabolic diseases [35, 36]. Among histone modifications, H3K4me3 serves as a critical marker of transcriptional activation and is dynamically balanced by histone methyltransferases and demethylases [21, 37], Dysregulation of this balance represents a core mechanism underlying several degenerative pathologies [38]. The H3K4me3 methylation landscape is governed by the methyltransferases of the MLL family (lysine methyltransferase [KMT]2A–D) and the SETD1 family (SETD1A, SETD1B), as well as the demethylases such as KDM5A–D and KDM1A/B [39]. For instance, in osteoarthritis, upregulation of the methyltransferase KMT2B increases H3K4me3 enrichment at promoters of WNT target genes, thereby activating matrix‐degrading enzymes and inflammatory mediators; pharmacological inhibition of H3K4me3 ameliorates cartilage damage [40]. Conversely, in neurodegenerative disorders such as Alzheimer's disease, decreased H3K4me3 levels lead to silencing of neuroprotective genes through reduced MLL1 activity and elevated KDM5A expression, while targeting these enzymes restores neuronal function [41]. Consistent with these reports, our data reveal that H3K4me3 levels are reduced in degenerative NP tissues, and suppression of H3K4me3 directly induces NPC senescence. These findings highlight the central role of H3K4me3 dysregulation in mediating degenerative processes within the IVD. However, our finding diverges from previous reports on the relationship between H3K4me3 and IDD [42, 43, 44], previous studies have demonstrated that the expression of H3K4me3 is significantly upregulated in senescent NPCs, and overexpression of KMT2A in the IVDs of rats can induce IDD. We speculate that the potential causes underlying such discrepancies mainly involve three aspects. First, previous studies mostly classified IDD samples according to the Pfirrmann grading system, whereas the present study focuses on the dynamic process of age‐related IDD, indicating an essential distinction in the research focus and grouping logic. Second, variations exist in in vitro studies. Previous studies used senescent primary HNPCs induced by tert‐Butyl hydroperoxide or Tumor Necrosis Factor‐α, while the present study adopted senescent primary RNPCs and HNPCs cell lines induced by DNA damage agents to explore the underlying mechanisms. Third, discrepancies lie in the intervention strategies of in vivo studies: previous studies implemented local intradiscal injection in rat IVDs, whereas the present study selected mouse models and performed intervention via tail vein injection with a significantly prolonged intervention duration, the differences in administration routes and experimental cycles may serve as crucial factors contributing to the divergent results. Combining our findings with those of previous studies, we propose that with advancing age, the global expression of H3K4me3 in NP tissues is downregulated. Collectively, H3K4me3 is closely associated with the process of IDD. As a transcription‐activating histone modification at gene promoter regions, H3K4me3 typically undergoes a rapid, dynamic increase during the acute phase of disease to drive the expression of pro‐inflammatory genes [45]. During the chronic degenerative phase, global epigenetic remodeling accompanied by localized abnormalities occurs, leading to the reduced expression of matrix synthesis‐related genes, as well as the downregulation of energy metabolism‐related genes and cell cycle‐related genes. In this study, we utilized rat and mouse models of naturally occurring IDD, a chronic degenerative model whose pathological state and associated gene expression alterations may differ substantially from those induced by acute stressors. These seemingly contrasting outcomes are not contradictory. As a transcription‐activating histone modification, the biological effects of H3K4me3 may not only depend on its global expression levels, but also on its locus‐specific modification patterns at target genes.

H3K4me3 is dynamically regulated by a diverse array of enzymes, and alterations in the expression or activity of distinct enzymes may exert markedly divergent effects on downstream target genes. Our findings demonstrate reduced SETD1A expression during IDD. As a member of the complex proteins associated with SET1 (COMPASS) complex, SETD1A regulates gene transcription via H3K4me3, influencing cell proliferation, the cell cycle, and senescence [16]. In fibroblasts, SETD1A promotes E2F and ID‐mediated transcription of cell‐cycle and DNA replication genes while repressing senescence [46]. Tajima et al. [17] further showed that SETD1A activates S‐phase kinase‐associated protein 2 via H3K4me3, suppressing p21 and p27 expression to prevent senescence of tumor cells. Consistently, our results reveal that SETD1A knockout accelerates IDD, underscoring its essential role in NPCs senescence regulation. Furthermore, overexpression of SETD1A alleviated senescence of NPCs and IDD progression.

The IVD is the largest avascular tissue in the body, relying primarily on glycolysis for energy metabolism even under normoxia. SETD1A has been implicated in glycolytic control and metabolic reprogramming across several disease contexts [47, 48], and our study confirms its regulatory role in NPC glycolysis. HIF1α is central to metabolic adaptation of NPCs within the IVD [30, 49], with HIF1α acting as a key transcriptional regulator of glycolysis [29] and a modulator of ECM synthesis, stress responses, and senescence [50]. Previous studies indicate that metabolic dysfunction contributes to IDD, whereas enhancing glycolysis, such as via HIF1α overexpression, can delay degeneration [27]. Meanwhile, HIF1α expression declines during IDD [51], When HIF1α expression is downregulated, oxidative stress intensifies, cellular senescence accelerates, and the progression of IDD is significantly advanced [52]. These studies consistent with our data showing the protective role of HIF1α in IVD homeostasis. However, recent research by Wang et al. indicates that the HIF1α signaling pathway is upregulated in degenerative human and mouse IVDs. Furthermore, the abnormal activation of HIF1α signaling pathway increases the risk of IDD, accompanied by enhanced glycolytic metabolism and impaired mitochondrial function [53]. Shi et al. [54] found that glycolytic function promotes ferroptosis of NPCs, thereby accelerating the process of IDD. In contrast, Dai and Zhang et al. [30, 55] suggests that glycolysis plays a crucial role in maintaining IVD homeostasis, and impaired glycolytic function accelerates IDD. Contradictory results about HIF1α and glycolysis roles in IDD stem mainly from heterogeneous research models, experimental strategies, and mechanistic focuses. In summary, glycolysis exerts a double‐edged sword effect in IDD. Physiological glycolysis is beneficial to disc homeostasis, whereas HIF1α‐induced excessive glycolysis promotes ferroptosis and IDD. Thus, standardized IDD staging and defined glycolytic thresholds are critical to reconciling these discrepancies and developing targeted therapies. Therefore, the specific effects of glycolysis on IDD warrant further investigation.

Undeniably, under normal conditions, HIF1α remains stable in NPCs and is closely associated with glycolytic function and senescence [25]. HIF1α is a key factor in maintaining the survival of NPCs and the synthesis of the extracellular matrix [56]. It is an essential factor for the expression of glycolytic genes (including LDHA), metabolic reprogramming, and animal survival under hypoxic conditions [57]. Importantly, HIF1α directly binds to and activates LDHA transcription, acting as a positive regulator of LDHA. Senescent cells exhibit reduced ATP production, and loss of glycolytic regulators such as LDHA disrupts ATP generation, exacerbating degeneration [26]. These findings reinforce the link between impaired glycolysis and NPC senescence. As HIF1α governs many functional aspects of NPC biology, it serves as a critical indicator in IDD pathogenesis and potential therapeutic targeting [58]. Our study identifies SETD1A as an upstream regulator of HIF1α; its downregulation reduces HIF1α expression, impairs glycolytic metabolism, and promotes cellular senescence. Moreover, HIF1α overexpression mitigates the deleterious effects of SETD1A loss, confirming its central role in the SETD1A‐mediated regulation of NPC metabolism and senescence.

Our omics analysis and experimental validation revealed that HELZ2 mediates the regulation of HIF1α by SETD1A. HELZ2, a zinc finger‐containing helicase and transcriptional coactivator, was initially identified as an interacting partner of the PPARα and PPARγ DNA‐binding domains [32, 59]. It regulates gene transcription by interacting with transcription factors and associated proteins [33]. In this study, we found that SETD1A directly promotes HELZ2 transcription through H3K4me3 enrichment at its promoter. Knockdown of HELZ2 reduced HIF1α expression, impaired glycolytic function, and induced senescence in NPCs. These findings, reported here for the first time, establish HELZ2 as a key mediator linking SETD1A to HIF1α regulation and as a novel regulator of NPC glycolysis and senescence, expanding the current understanding of HELZ2's biological functions.

HELZ2 typically functions as a nuclear co‐transcriptional activator that synergizes with PPARα. Upon heterodimerization, HELZ2 and PPARα recognize and bind to peroxisome proliferator‐activated receptor elements within gene promoters, modulating downstream transcription [33]. PPARα, a nuclear receptor central to lipid metabolism and energy balance, participates in diverse metabolic processes through transcriptional and protein–protein interactions [60]. Previous studies have demonstrated that PPARα binds extensively to promoters of glycolysis‐related genes, thereby regulating energy metabolism [61, 62]. Notably, PPARα also functions as a transcription factor for HIF1α [34]. promoting lipid utilization and delaying senescence in NPCs. Its expression decreases significantly with progressive IDD [63]. Our findings confirm that PPARα regulates NPC function both directly and through the SETD1A–HELZ2 axis. As a transcriptional activator of HIF1α, PPARα mediates the downstream effects of SETD1A and HELZ2 on glycolytic metabolism and cellular senescence, thereby integrating epigenetic and metabolic regulation in the pathogenesis of IDD.

This study has some limitations. First, rats and humans exhibit distinct spinal biomechanical properties, The primary cell model used in this study consists of primary RNPCs and HNPCs lines, which exhibit differences from human NPCs, which may influence the translational relevance of our findings. Additionally, most in vivo experiments were performed in C57BL/6 mice, whereas the in vitro assays utilized SD rat caudal NPCs. This discrepancy reflects the technical and cost challenges associated with isolating and maintaining mouse NPCs in culture, but it also introduces potential species‐specific regulatory differences. Moreover, adenoviral delivery by the tail vein may affect other organs, which warrants further investigations. At last, only male animals were used to ensure experimental reproducibility, leaving potential sex‐specific effects unaddressed.

## 4 Conclusions

In summary, this study identifies SETD1A as a critical epigenetic regulator of IDD that modulates glycolysis and senescence in NPCs through the HELZ2–PPARα–HIF1α signaling axis. SETD1A enhances H3K4me3 enrichment at the HELZ2 promoter, promoting HELZ2 transcription. Reduced SETD1A expression decreases HELZ2/PPARα co‐transcriptional activity, leading to diminished HIF1α expression, impaired glycolytic metabolism, accelerated NPC senescence, and aggravated IDD. Conversely, SETD1A overexpression restores HIF1α levels, enhances glycolytic capacity, delays cellular senescence, and mitigates IDD progression. These findings establish a comprehensive mechanistic framework linking epigenetic regulation to metabolic and degenerative processes within the IVD. By elucidating the SETD1A–HELZ2–PPARα–HIF1α pathway, this study not only expands the theoretical foundation of epigenetic control in IDD but also provides potential molecular targets for future therapeutic intervention.

## 5 Materials and Methods

### 5.1 Ethics

All procedures involving human participants were conducted in accordance with the ethical standards of the institutional and/or national research committee (Ethics Committee of the Second Affiliated Hospital, Army Medical University; approval no. 2025‐YD354‐01) and with the 1975 Helsinki Declaration, as revised in 2008. Informed consent was obtained from all individual participants included in the study.

All institutional and national guidelines for the care and use of laboratory animals were followed. Animal protocols were approved by the Animal Protection and Ethics Committee of the Army Medical University (approval no. AMUWEC20255372).

### 5.2 Reagents and Antibodies

The following reagents were used: OICR‐9429(10 µmol, 48 h), an inhibitor of the SETD1A–WDR5 complex that suppresses H3K4me3 deposition (cat. No. HY‐16993; MedChemExpress, Shanghai, China), BRACO‐19(50 µmol, 48 h), it is an effective telomerase/telomere inhibitor that can induce accelerated aging or selective cell death (cat. No. HY‐15523; MedChemExpress), dimethyloxalylglycine (DMOG(50 µmol, 48 h)); cat. No. HY‐15893; MedChemExpress), is a cell‐permeable, competitive HIF‐PH inhibitor that leads to the accumulation and stabilization of the hypoxia‐inducible factor 1‐alpha (HIF1α) protein. GW6471(25 µmol, 48 h) (cat. No. HY‐15372; MedChemExpress), is an effective peroxisome proliferator‐activated receptor alpha (PPARα) antagonist, and Oleoylethanolamide(OEA(50 µmol, 48 h)); cat. No. HY‐107542; MedChemExpress), a high‐affinity endogenous PPARα agonist. All reagents were incubated or processed for 48 h, unless otherwise specified. The list of antibodies, including dilution ratios and suppliers, is provided in Table S1.

### 5.3 Human Nucleus Pulposus (NP) Specimens

A total of 121 patients with varying ages were retrospectively analyzed, including medical records and lumbar spine magnetic resonance imaging (MRI) scans. Patient demographics were distributed as follows: 45 females, 76 males; 31 young (<36 years old), 75 middle (36–64 years old), and 15 old (>64 years old) individuals. Lumbar disc degeneration was graded using the Pfirrmann classification, as summarized in Table S2. Human NP tissues were collected from patients who underwent percutaneous endoscopic lumbar discectomy or microdiscectomy for lumbar disc herniation or spinal stenosis. Written informed consent was obtained from each patient prior to surgery. NP samples were processed according to immunofluorescence assay requirements. Among these, H3K4me3 was detected in 72 cases (23 females, 49 males), cyclin D2 (CCND2) in 19 cases (6 females, 13 males), SETD1A in 72 cases (23 females, 49 males), HIF1α in 26 cases (8 females, 18 males), and lactate dehydrogenase A (LDHA) in 9 cases (2 females, 7 males) (Table S3). Patients were stratified into three age groups: Young group (<36 years old), Middle group (36–64 years old), and Old group (>64 years old) [18].

### 5.4 Age‐Related IDD Models and Tail Vein Injection in Mice

#### 5.4.1 Age‐Related IDD Models

Male Sprague–Dawley (SD) rats aged 2 months (200 ± 20 g), 10 months (450 ± 45 g), and 20 months (750 ± 75 g) were obtained from the institutional animal facility. All rats were housed under specific pathogen‐free conditions with ad libitum access to standard chow and sterile water. Male C57BL/6J mice aged 2 months (25 ± 1.4 g), 10 months (34 ± 1.3 g), and 20 months (34 ± 3 g) were purchased from Charles River | VitoLiva (Chengdu, Sichuan, China). All animal experiments were conducted in compliance with institutional and national ethical guidelines.

#### 5.4.2 Tail Vein Injection Model

Twelve 12‐month‐old male C57BL/6N mice were randomly assigned to two groups: a control (CON) group (*n* = 6) and an AAV‐SETD1A treatment group (*n* = 6). Adeno‐associated virus (AAV) vectors encoding SETD1A or empty vector controls were constructed by Hanheng Biological Company (Globalebio, catalog no. GEGD‐Q9G). Mice were immobilized to expose the tail vein fully, and viral vectors were administered via an insulin syringe (Beilang, model U40) at a dose of 50 µL per injection, once per month, for a total of seven injections. Animals were monitored daily postinjection for health status and were allowed free access to food and water. At 20 months of age, the caudal IVDs were harvested for subsequent analyses. Construction of Information (Table 1).

**TABLE 1:** Primers used for Construction of SETD1A Adenovirus Vector.

Target gene	Primer sequence (5'‐3')	
	Forward	Reverse
Mouse Setd1a‐K/X	gctgtgaccggcgcctactctggtaccgccaccatggaccaggaaggtg	ttgtcatcgtcatccttgtagtctcgaggttgagggagccacggcaac
Mouse Setd1a	gctcttcatctcttggacaagggtagccagcacacgaccca	ttgtccaagagatgaagagcattatgcagcgagaccttaat

### 5.5 SETD1A Conditional Knockout (cKO) Mouse Model

Aggrecan (Acan)‐CreER*^T2^* (stock no.019148, the Jackson Laboratory, Bar Harbor, ME, USA) was crossed with SETD1A*^fl/fl^* mice (C57BL/6 background; Cyagen Biosciences, Suzhou, China) to generate Acan‐CreER*^T2^*; SETD1A*^fl/fl^* conditional knockout (SETD1A‐cKO, hereinafter referred to as cKO) mice. To induce Cre recombinase activity in Acan‐expressing cells, tamoxifen (MedChemExpress, cat no. HY‐13757A) was administered intraperitoneally at 1 mg/10 g body weight daily for five consecutive days to 2‐week‐old male mice. Age‐matched wild‐type (WT) C57BL/6 males treated with tamoxifen served as controls. Construction of Information (Table 2).

**TABLE 2:** Primers used for cKO determined genotypes.

Target	Primer sequence (5'‐3')
Mouse Acan‐CreERT2: common	AAAAGCGACAAGAAGACACCA
Mouse Acan‐CreERT2: mutant forward	CTCCAGACTGCCTTGGGAAAA
Mouse Acan‐CreERT2: wild type forward	GTTATATTCCGGAGCCCACA
Mouse SETD1Afl/fl: forward	GCCTATAACTTGGTTTTGTGGGG
Mouse SETD1Afl/fl: reverse	ACACTGGATATCTCCCATTGTTGTA

MRI examinations were performed at 2, 10, and 20 months of age to monitor intervertebral disc morphology. At 20 months, micro‐computed tomography (µCT), histological staining, and immunofluorescence analyses were conducted to evaluate IDD.

### 5.6 Measurement of Glycolytic Activity

#### 5.6.1 Lactate and ATP Content

Cells were seeded in T25 flasks, and culture supernatants were collected at designated time points. Extracellular lactate concentrations were determined using an L‐Lactic Acid (L‐LA) Content Detection Kit (Solarbio, BC2230, Beijing, China), following the manufacturer's protocol. Intracellular ATP levels were measured using an ATP Content Detection Kit (Solarbio, BC0305). After removing the supernatant, cells were counted and normalized to a cell number. Detection unit µmol/mL.

#### 5.6.2 Glucose Uptake

Cellular glucose uptake was evaluated using a Glucose Uptake Fluorescence Assay Kit with 2‐NBDG (Beyotime, S0561M). 2‐NBDG is a fluorescently labeled deoxyglucose analog that enters cells via glucose transporters. After phosphorylation by hexokinase, it remains intracellularly and accumulates within cells. Consequently, the fluorescence produced by 2‐NBDG is directly proportional to cellular glucose uptake. Collect 800 000 NPCs for cell counting, then proceed according to the kit protocol. After incubation with fluorescent substrate, images were captured using a confocal laser scanning microscope (spinFV‐COME, Olympus, Tokyo, Japan). Quantitative fluorescence intensity was analyzed using ImageJ software.

### 5.7 Chromatin Immunoprecipitation Sequencing (ChIP‐seq) and ChIP‐qPCR

NP tissues were isolated from 2‐month‐old and 20‐month‐old SD rats, take 15 rats per group, and extract the NP from the 2nd to 10th vertebrae of each rat. Tissues were enzymatically digested to obtain single‐cell suspensions before proceeding with ChIP processing (PRJNA1416674). For RNPCs ChIP‐seq, samples were prepared from shNC and shSETD1A groups following lentiviral transfection, perform three biological replicates, each with 3 000 000 cells. Each condition included three independent biological replicates. After digestion with trypsin to obtain single‐cell suspensions, cells were washed with phosphate‐buffered saline (PBS) and processed according to the previously established ChIP and qPCR protocols [64].

ChIP–enriched DNA fragments underwent end repair, 3′‐end adenylation, and adapter ligation. DNA fragments of the target size were recovered using magnetic bead purification and PCR amplification to construct sequencing libraries. Library quality was verified by agarose gel electrophoresis, and DNA concentration was quantified using Qubit 2.0 fluorometer (Thermo Fisher Scientific). Qualified libraries were pooled according to effective concentrations and data yield requirements, and sequenced on an MGISEQ‐T7 platform (Shenzhen, China). Raw sequencing reads were filtered to remove low‐quality data, generating clean reads, which were aligned to the reference genome. Peak calling was performed with MACS2 to identify binding regions. Genome‐wide peak annotation and motif enrichment analyses were used to determine protein‐DNA binding preferences. ChIP‐qPCR validation was performed using specific primers targeting peak regions, with input DNA as a control, and analyzed via standard qPCR procedures. Primer information is shown in Table 3.

**TABLE 3:** Primers used for ChIP‐qPCR.

Primers used for ChIP‐qPCR		
Target gene	Primer sequence (5'‐3')	
	Forward	Reverse
Rat ChIP‐qPCR: HELZ2	GCTAGGGTCTGTCTGAAGCC	TCGCTGCTCTAAGTCGATGC

### 5.8 Statistical Analyses

All experiments were performed with at least three biological replicates. For continuous data, the Shapiro‐Wilk test and Levene's test were first applied to assess the normality of distribution and homogeneity of variances, respectively. If the data exhibited a non‐normal distribution, normalization was attempted via square‐root or logarithmic transformation, and the results were presented as mean ± standard deviation (SD). For continuous data that satisfied the assumptions of normality and homogeneity of variances, the unpaired two‐tailed Student's *t*‐test was used for comparisons between two groups, whereas one‐way analysis of variance (one‐way ANOVA) followed by Tukey's posthoc test was employed for multi‐group comparisons. For experiments with a two‐factor mixed design, two‐way ANOVA was performed. If normality could not be achieved even after data transformation, the results were expressed as median ± interquartile range (IQR), with the Mann–Whitney U test for two‐group comparisons and the Kruskal‐Wallis H test for multi‐group comparisons. For data with normal distribution but heterogeneous variances, Welch's ANOVA was used for multi‐group comparisons. Chi‐square test was adopted for the analysis of categorical variables. All statistical analyses were conducted using GraphPad Prism and SPSS software. A value of *p* < 0.05 was considered statistically significant.

## Author Contributions

Designing the study: Jiawei Fu, Chencheng Feng, Yue Zhou, and Shiwu Dong. Conducting experiments: Jiawei Fu, Xue Leng, Jiang Long, Xuezheng Ai, Zhengao Liao, Xiaoqin AN, and Dan Long. Acquiring data: Jiawei Fu, Chencheng Feng, Bo Huang, Shiwu Dong, and Changqing Li. Analyzing data: Jiawei Fu, Xue Leng, and Jiang Long. Collecting clinical samples: Chencheng Feng and Bo Huang. Writing and revising the manuscript: Jiawei Fu, Chencheng Feng, and Yue Zhou.

## Funding

This study was supported by National Natural Science Foundation of China (82372467, 81902255, 82072495), China Postdoctoral Science Foundation General Program (2020M673652), Natural Science Foundation of Chongqing in China (CSTB2024 NSCQ‐MSX1155), Special Project for Enhancing Scientific and Technological Innovation at the Army Medical University (2020XON15), Young Doctoral Talent Incubation Program The Second Affiliated Hospital of Army Medical University (2023XKRC013).

## Conflicts of Interest

The authors declare no conflicts of interest.

## Supporting information




**Supporting File 1**: advs75105‐sup‐0001‐SuppMat.docx.

**Supporting File 2**: advs75105‐sup‐0002‐TableS1.docx.

**Supporting File 3**: advs75105‐sup‐0003‐TableS2.docx.

**Supporting File 4**: advs75105‐sup‐0004‐TableS3.docx.

**Supporting File 5**: advs75105‐sup‐0005‐TableS4.docx.

**Supporting File 6**: advs75105‐sup‐0006‐TableS5.docx.

## Data Availability

All data are available from the corresponding authors upon reasonable request.
