# Supplementary material for: SETD1A Regulates Glycolysis and Senescence of Nucleus Pulposus Cells via H3K4me3–HELZ2/PPARα‐HIF1α Axis to Drive Intervertebral Disc Degeneration
Source: Adv Sci (Weinh). 2026 Mar 31;13(34):e75105. doi: 10.1002/advs.75105 (PMC13285123; doi:10.1002/advs.75105)
Supplement: Supplementary file 1 — Supporting File 1: advs75105‐sup‐0001‐SuppMat.docx. [file ADVS-13-e75105-s001.docx]

**Supplementary Materials**

This file includes:

Supplementary Materials and Methods

Supplementary Figure legends 1 to 8

Original uncropped WB

**Supplementary Materials and Methods**

*Cell culture and treatments*

***Human NP cells (HNPCs)***

Human immortalized primary nucleus pulposus cells (HNPCs; iCell, cat. no. iCell-0028a) were cultured in the corresponding complete medium (iCell-0028a-001b) at 37 °C in a humidified incubator containing 5 % CO₂. For recovery, frozen cells were rapidly thawed at 37 °C, centrifuged to remove dimethyl sulfoxide (DMSO), resuspended in complete medium, and the medium was replaced after 24 h. When cultures reached 80–90% confluence, cells were digested with 0.25% trypsin-EDTA and sub-cultured at a 1:2 ratio. Cells at 80–90% confluence were used for subsequent experiments. This section has undergone ethical review.

***Rat NP cells (RNPCs)***

Primary rat nucleus pulposus cells (RNPCs) were isolated following a previously described protocol[1]. Briefly, NP tissues were aseptically dissected, digested enzymatically, and cultured under standard conditions for use in downstream experiments.

***Lentiviral transfection***

For gene silencing, lentiviruses encoding short hairpin RNAs (shRNAs) targeting SETD1A or helicase with zinc finger 2 (HELZ2) were used to infect RNPCs and HNPCs, following previously reported methods [1]. The corresponding negative controls were designated shNC. Knockdown groups were referred to as shSETD1A and shHELZ2. For gene overexpression, lentiviral vectors carrying SETD1A or HELZ2 coding sequences were transduced into cells, with vector-only controls (oeNC) serving as references. The resulting stable lines were termed oeSETD1A and oeHELZ2. Species and Construction Information show in Supplementary Table S4. Lentivirus was transduced into ~2 × 10⁵ cells (30% confluence) at 15 MOI. 24-hour resistance screening, Passage or assay should be performed when cell confluence reaches 80%.

*Magnetic resonance imaging (MRI) and micro-computed tomography (μCT)*

MRI was performed to evaluate T2 signal intensity of intervertebral discs, using previously established acquisition parameters [1].

For µCT analysis, mice were euthanized, and lumbar spines were excised and fixed in 4% paraformaldehyde (PFA) for 24–48 h. Samples were scanned using a high-resolution μCT system (SkyScan 1272, Bruker, Berlin, Germany) at 65 kV and 153 μA, with a voxel resolution of 5.0 μm per pixel. Image reconstruction was performed with NRecon v1.7, quantitative analyses with CTAn v1.2, and 3-D visualization with CTvox v3.3. Quantitative parameters included disc height and volume fraction, tissue volume, percent bone volume, trabecular thickness, trabecular number.

*Histological staining*

Histological evaluation of IDD severity was performed using hematoxylin–eosin (H&E), Safranin O(S-FG), and Masson’s trichrome staining, as described previously [1]. H&E staining was used to assess general tissue morphology and degeneration; Safranin O staining visualized glycosaminoglycans (GAGs) and cartilage matrix components to determine the extent of degeneration; and Masson’s trichrome staining was used to evaluate fibrosis and collagen deposition.

*Immunofluorescence staining*

To assess protein localization and expression, paraffin-embedded or frozen tissue sections were prepared, and cultured cells were seeded on confocal dishes before fixation. Immunofluorescence procedures were performed as previously described, including antigen retrieval, blocking, incubation with primary and fluorescent secondary antibodies, and nuclear counterstaining with DAPI. [2]. Immunofluorescence imaging was performed using a 3DHistech instrument, model Pannoramic MIDI. Quantitative analysis using ImageJ, the obtained grayscale values were converted into multiples and subjected to statistical analysis[3].

*Quantitative real-time polymerase chain reaction (qPCR)*

Total RNA was extracted from NP cells using TRIzol™ reagent (Invitrogen, Shanghai, China). RNA purity and concentration were determined using a NanoDrop ND-1000 spectrophotometer (Thermo Fisher Scientific, Waltham, MA, USA). cDNA was synthesized using the PrimeScript RT-PCR Kit (Takara) qPCR was performed using a QuantStudio RT-qPCR system (Thermo Fisher Scientific) under the following thermal cycling conditions: 95 °C for 30 s, followed by 40 cycles of 95 °C for 5 s and 60 °C for 30 s. β-Actin was used as the endogenous reference gene. Relative mRNA expression levels were calculated using the 2⁻ΔΔCt method. Primer sequences are provided in (Supplementary Table S5)

*Western blot analysis*

To compare protein expression levels, NP cells were harvested at 80–90% confluence, proteins extracted, and subjected to protein expression analysis according to the previously established protocol [1]].

*Cell proliferation and senescence assays*

***Cell proliferation (CCK-8 Assay)***

NP cell proliferation was evaluated using a Cell Counting Kit-8 (CCK-8; Solarbio, CA1210). Cells were seeded in 96-well plates (1,000 cells/well) and incubated for 24, 48, and 72 h. After adding 10 µL of CCK-8 reagent to each well (final volume 100 µL) and incubating for 2 h, absorbance was measured at 450 nm using a microplate reader (SpectraMax M2).

***Senescence-associated β-galactosidase (SA-β-Gal) staining***

Cellular senescence was detected using an SA-β-Gal staining kit (Solarbio, cat no. G1580). Cells cultured on BioFlex™ plates were fixed with 2% formaldehyde for 25 min, incubated with X-gal (1 mg/mL) at 37 °C for 12 h, and observed under a light microscope (Olympus, Tokyo, Japan). The percentage of SA-β-Gal–positive cells was quantified in nine random fields at 200× magnification.

*mRNA sequencing*

Tissue samples were collected from caudal intervertebral discs of WT (n = 4) and cKO (n = 4) mice. Cell samples included those treated with OICR-9429 (control, n = 3; OICR-9429, n = 3) and SETD1A knockdown (shNC, n = 3; shSETD1A, n = 3). Total RNA was extracted from tissues and cells using TRIzol reagent (Thermo Fisher Scientific, cat. no. 15596026) following the manufacturer’s protocol. RNA samples were treated with DNase I (New England Biolabs, cat. no. M0303L) to remove genomic DNA contamination. RNA purity was assessed by NanoDrop OneC spectrophotometer (Thermo Fisher Scientific), integrity verified using LabChip GX Touch (Revvity), and concentration quantified by Qubit 3.0 fluorometer with the Qubit™ RNA Broad Range Assay Kit (Thermo Fisher Scientific, cat. no. Q10210). RNA sequencing (RNA-seq) libraries were prepared with the KCTM Digital mRNA Library Prep Kit (Seqhealth Tech. Co., Ltd., Wuhan, China), which incorporates 12-base unique molecular identifiers (UMIs) to minimize PCR and sequencing bias. cDNA fragments (200–500 bp) were PCR-amplified and purified for library enrichment. Library quality and yield were confirmed before sequencing on an Illumina NovaSeq X Plus platform using paired-end 150 bp (PE150) reads. Each biological condition was analyzed in triplicates.​

*Proteomic analysis*

Tail disc tissues from WT (n = 4) and SETD1A-cKO (n = 4) mice were lysed in 1.5 % SDS/100 mM Tris-HCl (pH 8.5; Sigma-Aldrich), homogenized, heated at 95 °C for 15 min, sonicated, and centrifuged. Pellets were resuspended in 8 M urea/Tris-HCl (pH 8.5; Sigma-Aldrich), and protein concentration was determined using a BCA Protein Assay Kit (Thermo Fisher Scientific).​ Proteins were reduced and alkylated with tris(2-carboxyethyl)phosphine (TCEP) and chloroacetamide (CAA) (Thermo Fisher Scientific) at 37 °C for 1 h, and urea was diluted to <2 M. Digestion was performed overnight at 37 °C using trypsin (Promega) at a 1:50 enzyme-to-protein ratio, followed by quenching with trifluoroacetic acid (TFA) (pH 6.0; Sigma-Aldrich). Peptides were desalted using SDB-RPS cartridges (Thermo Fisher Scientific), dried, and stored at −20 °C.​ For quantitative analysis, peptides were labeled with Tandem Mass Tag (TMTpro) reagents (channels 126, 127N/C, 128N/C, 129N/C, 130N; Thermo Fisher Scientific). Labeled peptides were mixed in equal proportions, desalted using Sep-Pak C18 cartridges (Waters), dried, and fractionated into 15 fractions via high-pH reversed-phase chromatography. Liquid chromatography–tandem mass spectrometry (​LC-MS/MS) was performed using an UltiMate 3000 UHPLC system coupled to a Q Exactive HF mass spectrometer (Thermo Fisher Scientific) in data-dependent acquisition (DDA, top-15) mode with higher-energy collisional dissociation (HCD) fragmentation. Raw spectra were analyzed using MaxQuant v1.6.17.0, searched against the UniProt Mouse database (UP000000589). Parameters included TMT quantification, cysteine carbamidomethylation as a fixed modification, and trypsin/P specificity, with a 1% false discovery rate (FDR). Differentially expressed proteins (DEPs) were identified by two-tailed *t*-test using R v4.3.0. Functional enrichment analyses for Gene Ontology (GO) and Kyoto Encyclopedia of Genes and Genomes (KEGG) pathways, as well as protein–protein interaction (PPI) network construction, were conducted using STRING v11.5.

**Supplementary Figures and figure legends**

**
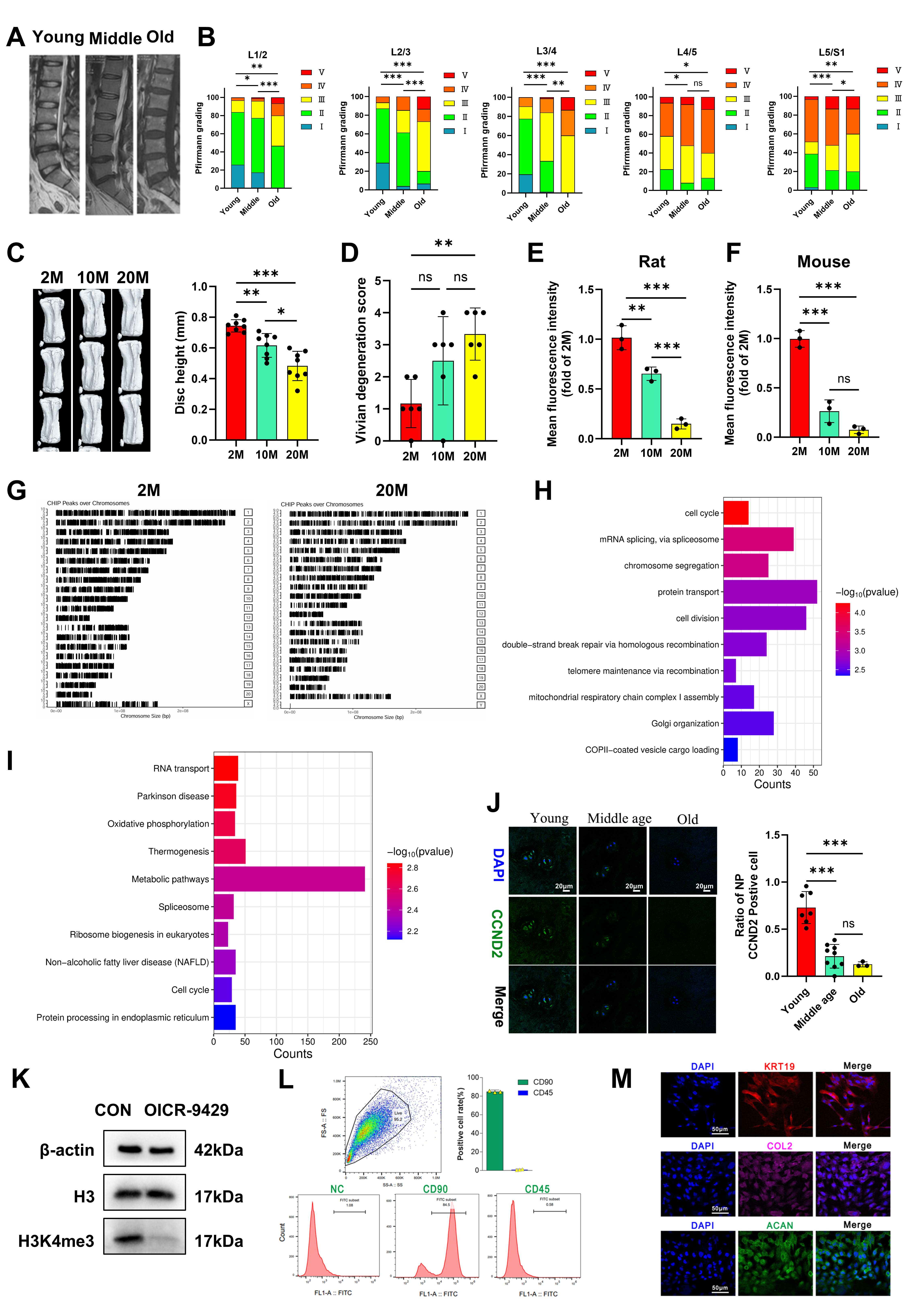
**

**Fig. S1. Age-related decrease of H3K4me3 expression and validation of IDD models.**

(A) Representative MRI images of human lumbar IVDs at different ages. (B) Distribution of Pfirrmann grades across lumbar disc levels presented as bar graphs (Young: n = 31; Middle: n = 75; Old: n = 15)*.* (C) Representative μCT imaging of mouse caudal IVDs at different ages and quantitative measurement of intervertebral disc height (n = 8; 2 IVDs per mouse, 4 mice total). (D) Vivian degeneration score of mice caudal IVD sections based on H&E staining (n = 6). (E, F) Quantification of average fluorescence intensity of H3K4me3 in rat (E) and mouse (F) NP tissue in caudal IVDs at different ages (n = 3). (G) chromosomal distribution of H3K4me3 peaks in rat NP tissues with different ages. (H) GO enrichment analysis of the genes with differential enriched H3K4me3 peaks, and (I) KEGG enrichment analysis of the genes with differential enriched H3K4me3 peaks. (J) Representative immunofluorescence staining of CCND2 in human lumbar NP tissues from different age groups. Quantification of CCND2-positive NPCs is shown (Young: n = 7; Middle age: n = 9; Old: n = 3). (K) Western blot analysis of protein in RNPCs treated with indicated agents. (L) Flow cytometric characterization of HNPCs. (M) Immunofluorescence validation of HNPC identity. **p* < 0.05, ***p* < 0.01, ****p* < 0.001, ns = not significant. (Chi-square test; one-way ANOVA)

**
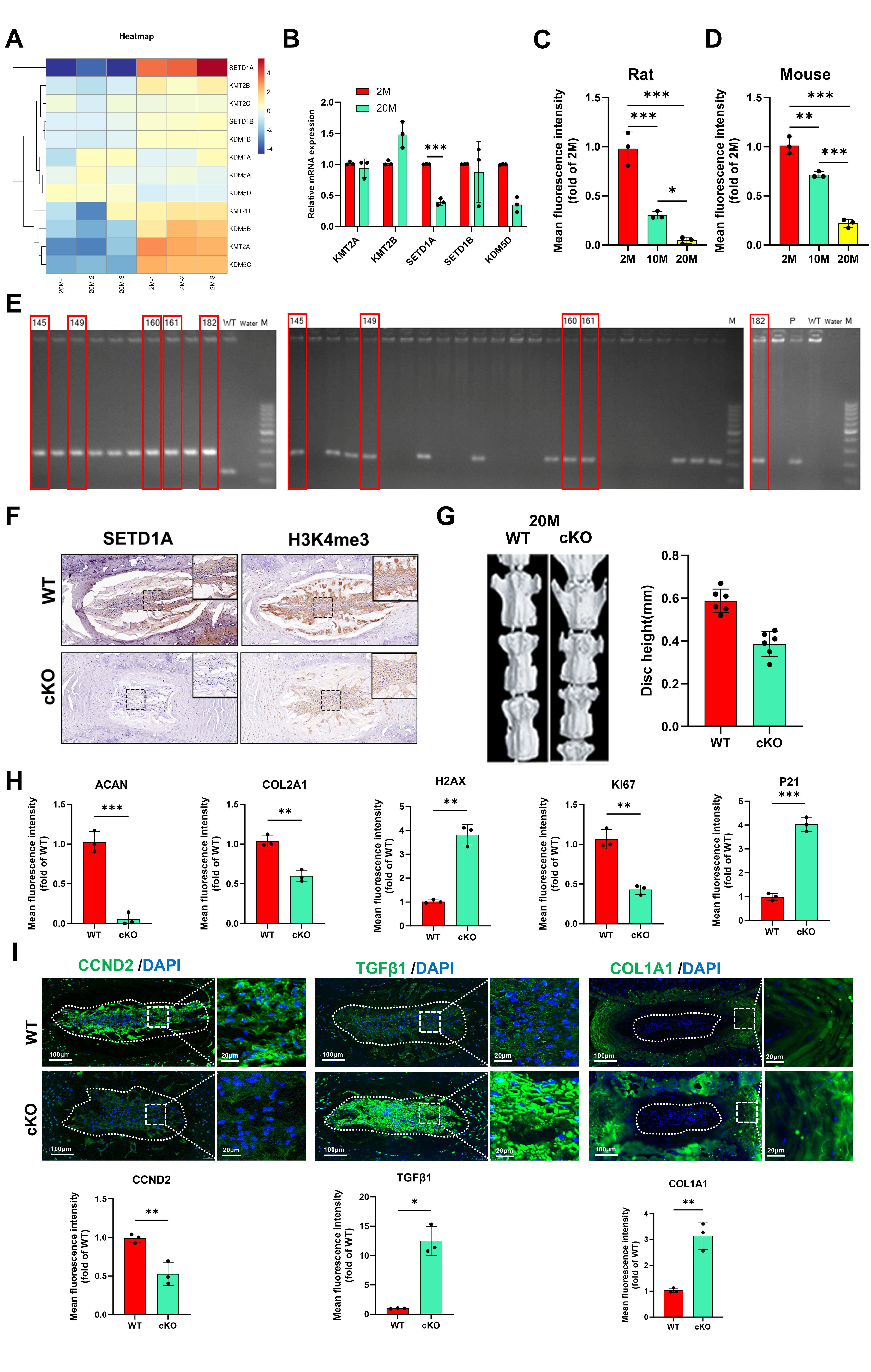
**

**Fig. S2. Downregulation of SETD1A and H3K4me3 in degenerative IVDs and validation of SETD1A knockout mouse model.**

(A) Heatmap shows expression changes of histone H3K4me3 methyltransferases and demethylases in caudal NP tissues from 2-month-old (2M) and 20-month-old rats (20M). (B) mRNA expression of H3K4me3 methyltransferases and demethylases in rat caudal NP tissues detected by qPCR (n = 3). (C, D) Quantification of average fluorescence intensity of SETD1A in rat (C) and mouse (D) caudal NP tissues with different ages (n = 3). (E) Gel electrophoresis verification of SETD1A-cKO mice. (F) Representative immunohistochemical staining of SETD1A and H3K4me3 in NP tissue of WT and cKO mice*.* (G) Representative μCT imaging of mouse caudal IVDs in different groups and quantification of IVD height (n = 6; 2 IVDs per mouse, 3 mice total). (H) Immunofluorescence staining quantitative analysis of Fig. 2H and I (n = 3). (I) Representative immunofluorescence staining of indicated markers in caudal IVDs from WT and cKO mice of different ages and quantification of average fluorescence intensity within NP tissues (n = 3). **p* < 0.05, ***p* < 0.01, ****p* < 0.001, ns = not significant. (Unpaired t-test; Mann-Whitney U test; one-way ANOVA)

**
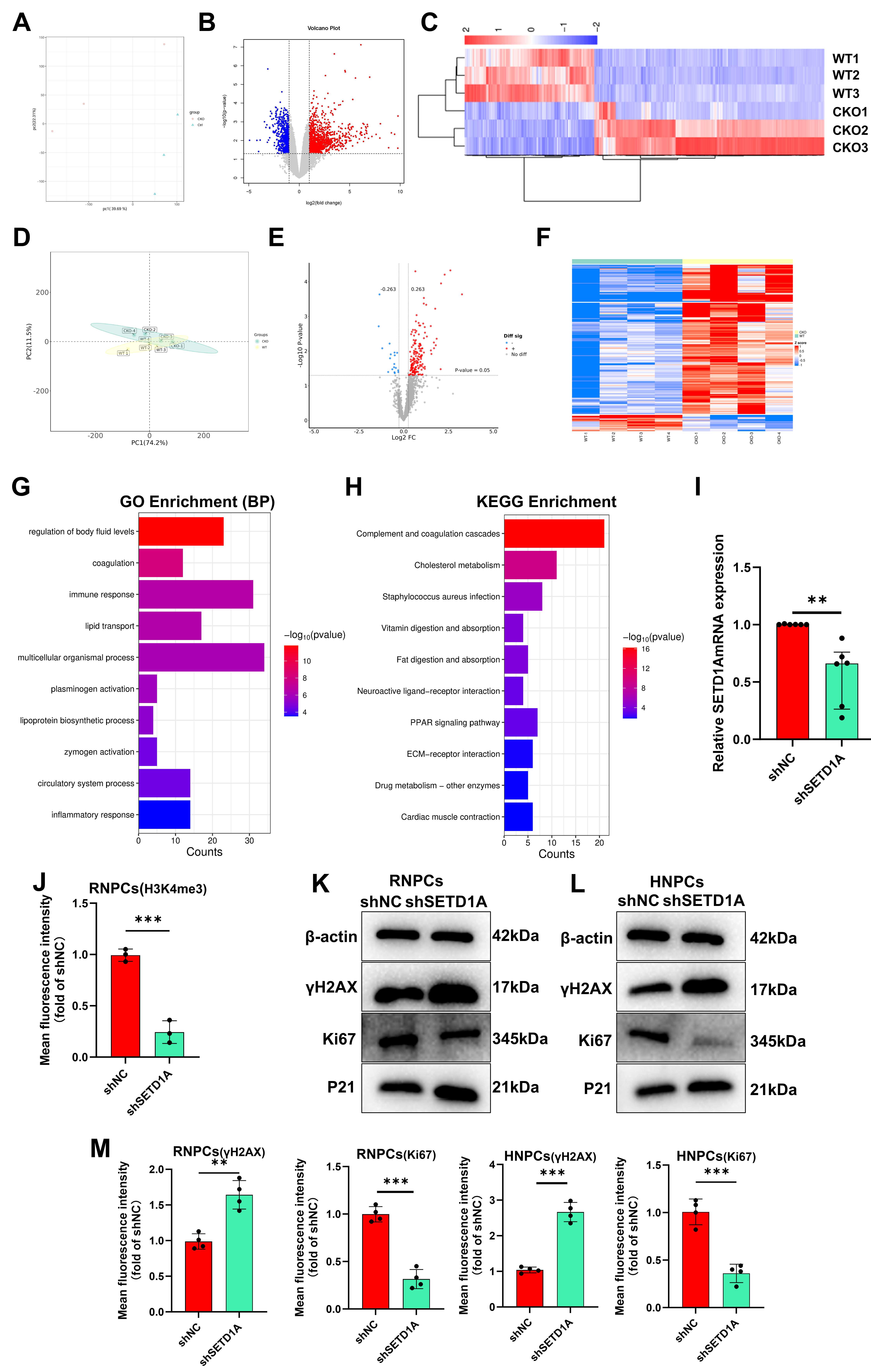
**

**Fig. S3. Transcriptomic and proteomic profiling of caudal discs from SETD1A conditional knockout mice.**

(A–C) Transcriptomic analysis of caudal IVD tissues from WT and SETD1A-cKO mice (n = 3). (A) PCA of mRNA-seq data. (B) Volcano plot showing DEGs of caudal discs between WT and SETD1A-cKO mice. Fold-change (x-axis) is plotted against statistical significance (y-axis). Red quadrants indicate upregulated genes, and blue quadrants indicate downregulated genes (fold change ≥ 2, *p* < 0.05). (C) Heatmap of DEGs between WT and SETD1A-cKO mice. (D–F) Proteomic analysis of caudal IVD tissues from WT and SETD1A-cKO mice (n = 4). (D) PCA plot of proteome data. (E) Volcano plot showing differentially expressed proteins between WT and SETD1A-cKO mice. (F) Heatmap of differentially expressed proteins. (G) GO analysis of differentially expressed proteins. (H) KEGG analysis of differentially expressed proteins. (I) mRNA expression of SETD1A in HNPCs detected by qPCR (n = 6). (J) Immunofluorescence quantification analysis of Fig. 3F (n = 3)*.* (K, L) Western blot of γH2AX, Ki67 and P21 in NPCs at different treatment conditions. (M) Immunofluorescence quantification analysis of Fig. 3I (n = 3)*.* **p* < 0.05, ***p* < 0.01, ***p* < 0.001, ns = not significant. (Unpaired t-test; Mann-Whitney U test)

**
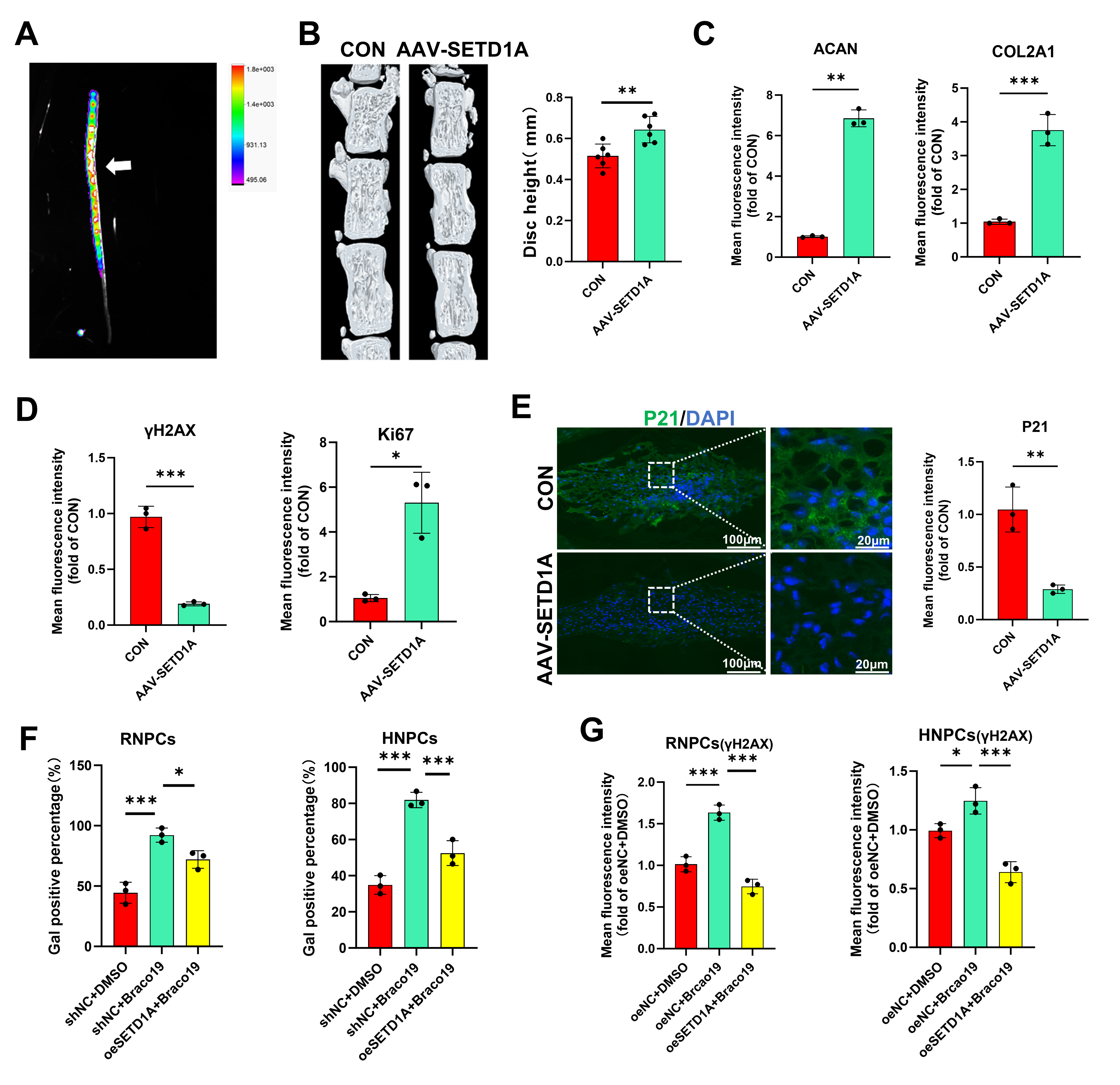
**

**Fig. S4. Validation of SETD1A overexpression and associated effects on IVDs and NP cells.**

(A) Distribution of fluorescent signals in sagittal sections of mouse caudal IVDs following tail vein injection of fluorescently labeled adenovirus. (B) Representative μCT images of mouse IVDs at different ages and quantification of intervertebral disc height (n = 6; 2 IVDs per mouse, 3 mice total). (C, D) Quantification of average fluorescence intensity of indicated markers within defined regions of interest of Fig. 4E and F (n = 3). (E) Representative immunofluorescence staining of P21 in caudal IVDs from mice of different treatment conditions and quantification analysis (n = 3). (F) Quantification of SA-β-Gal-positive HNPCs and RNPCs treated with different agents (n = 3). (G) Immunofluorescence quantification analysis of NPCs of Fig. 4I (n = 3). **p* < 0.05, ***p* < 0.01, ****p* < 0.001, ns = not significant. (Unpaired t-test; one-way ANOVA)

**
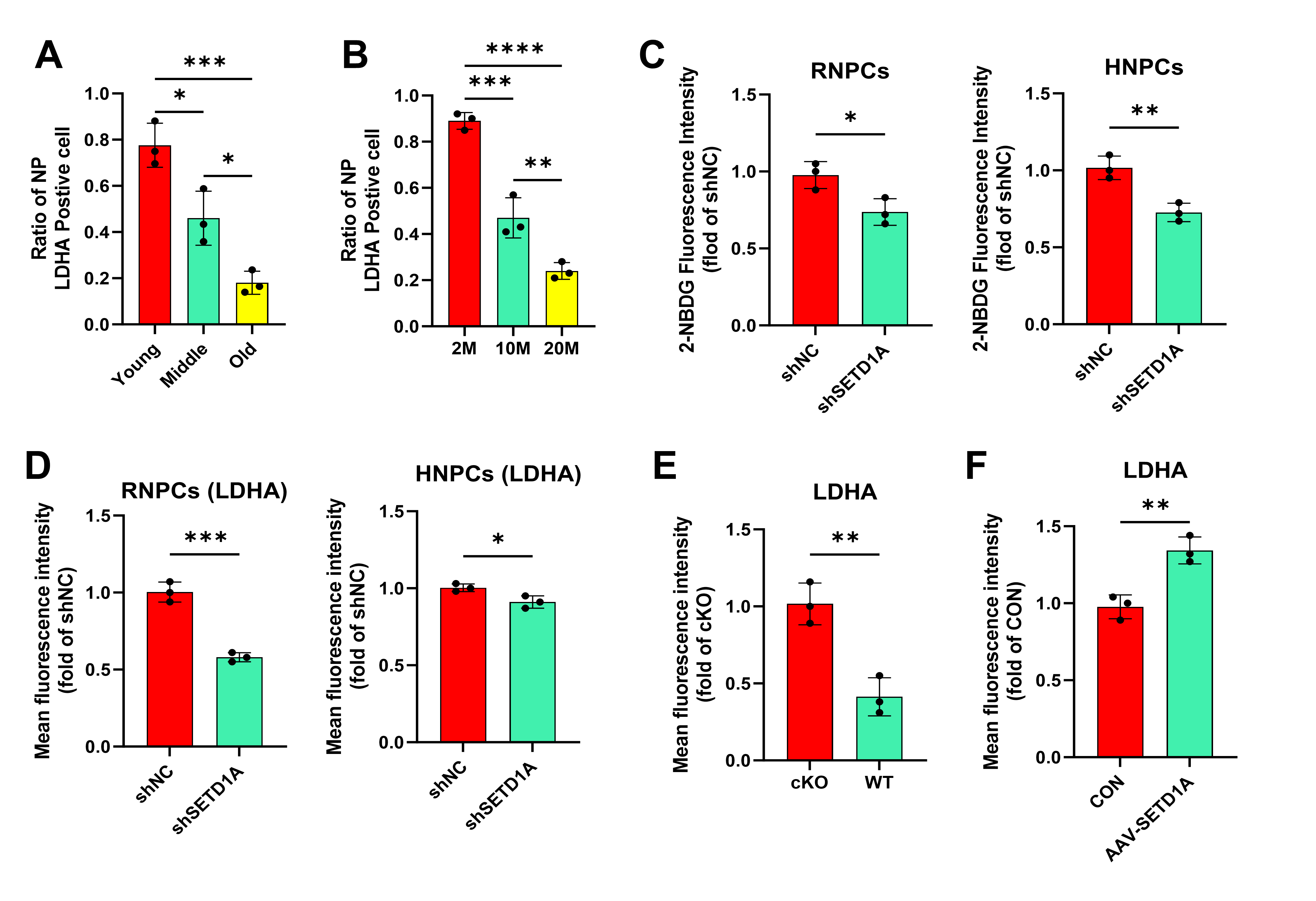
**

**Fig. S5. SETD1A regulates glycolysis as a key determinant of NPC senescence.**

(A)Immunofluorescence quantification analysis of Fig. 5E (n = 3). (B) Immunofluorescence quantification analysis of Fig. 5F (n = 3). (C) Immunofluorescence quantification analysis of Fig. 5I (n = 3). (D) Immunofluorescence quantification analysis of Fig. 5K (n = 3). (E) Immunofluorescence quantification analysis of Fig. 5L (n = 3). (F) Immunofluorescence quantification analysis of Fig. 5N (n = 3). *p < 0.05, **p < 0.01, ***p < 0.001. (Unpaired t-test; one-way ANOVA)

**
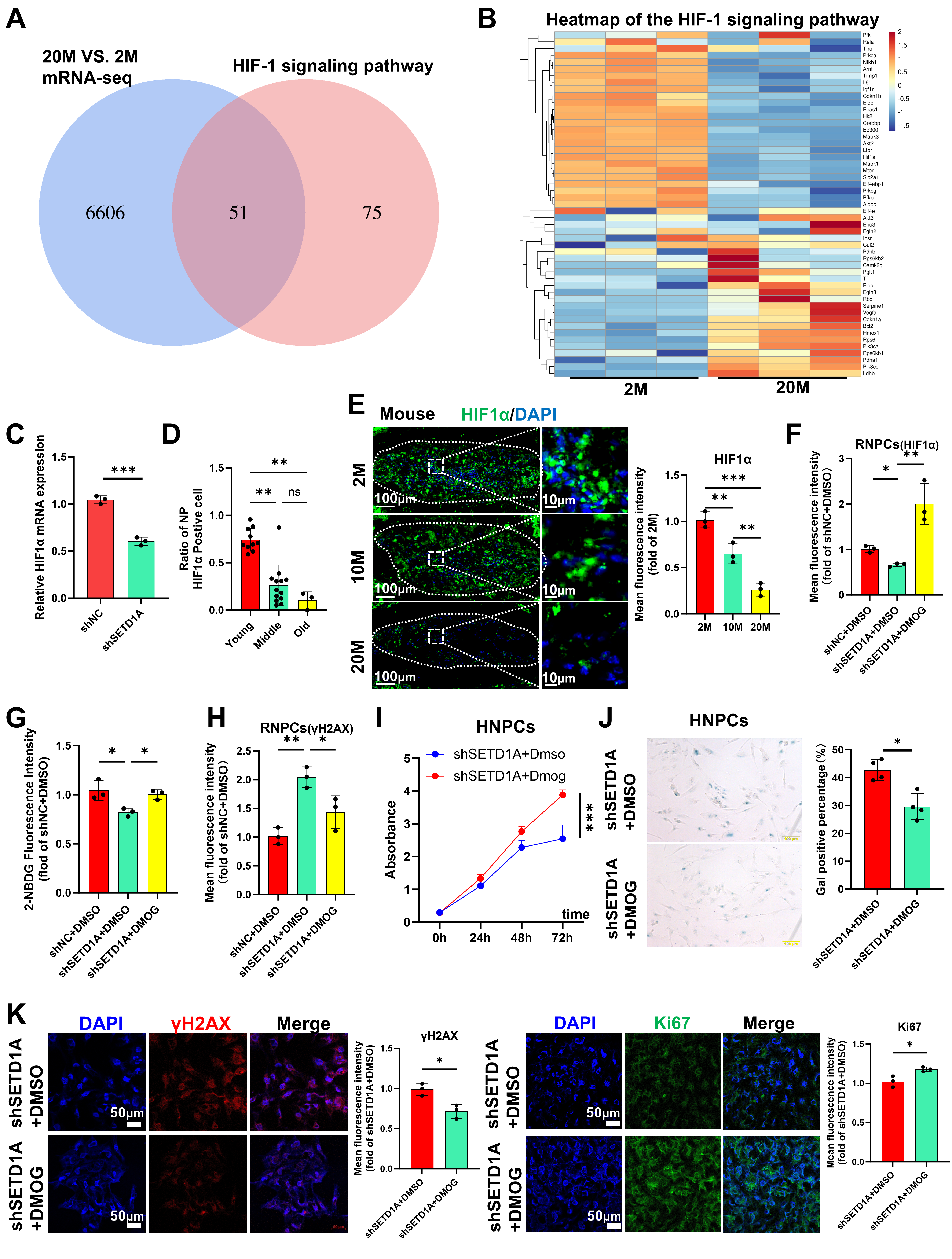
**

**Fig. S6. HIF1α expression patterns and functional validation in NPCs.**

(A) Overlap analysis of DEGs of rat NP tissues with aging and the genes of HIF-1 Signaling Pathway. (B) Heatmap of differentially expressed genes enriched in the HIF‑1 signaling pathway. (C) mRNA expression of HIF1α detected by qPCR in HNPCs (n = 3). (D) Quantification of H3K4me3-positive NPCs in human lumbar NP tissues with different ages (Young < 36 years, n = 10; Middle 35–65 years, n = 13; Old > 64 years, n = 3). (E) Representative immunofluorescence staining of H3K4me3 in caudal IVDs of mice at different ages and quantification analysis (n = 3). (F) Immunofluorescence quantification analysis of Fig. 6I (n = 3). (G) Immunofluorescence quantification analysis of Fig. 6L (n = 3). (H)Immunofluorescence quantification analysis of Fig. 6P (n = 3). (I) Proliferation of HNPCs assessed by CCK-8 assay (n = 3). (J) SA-β-Gal staining and quantification of SA-β-Gal-positive HNPCs treated with different agents (n = 3). (K) Representative immunofluorescence staining of γH2AX and Ki67 in HNPCs exposed to indicated treatments and quantification analysis (n = 3). **p* < 0.05, ***p* < 0.01, ****p* < 0.001, ns = not significant. (Unpaired t-test; one-way ANOVA; two-way ANOVA)

**
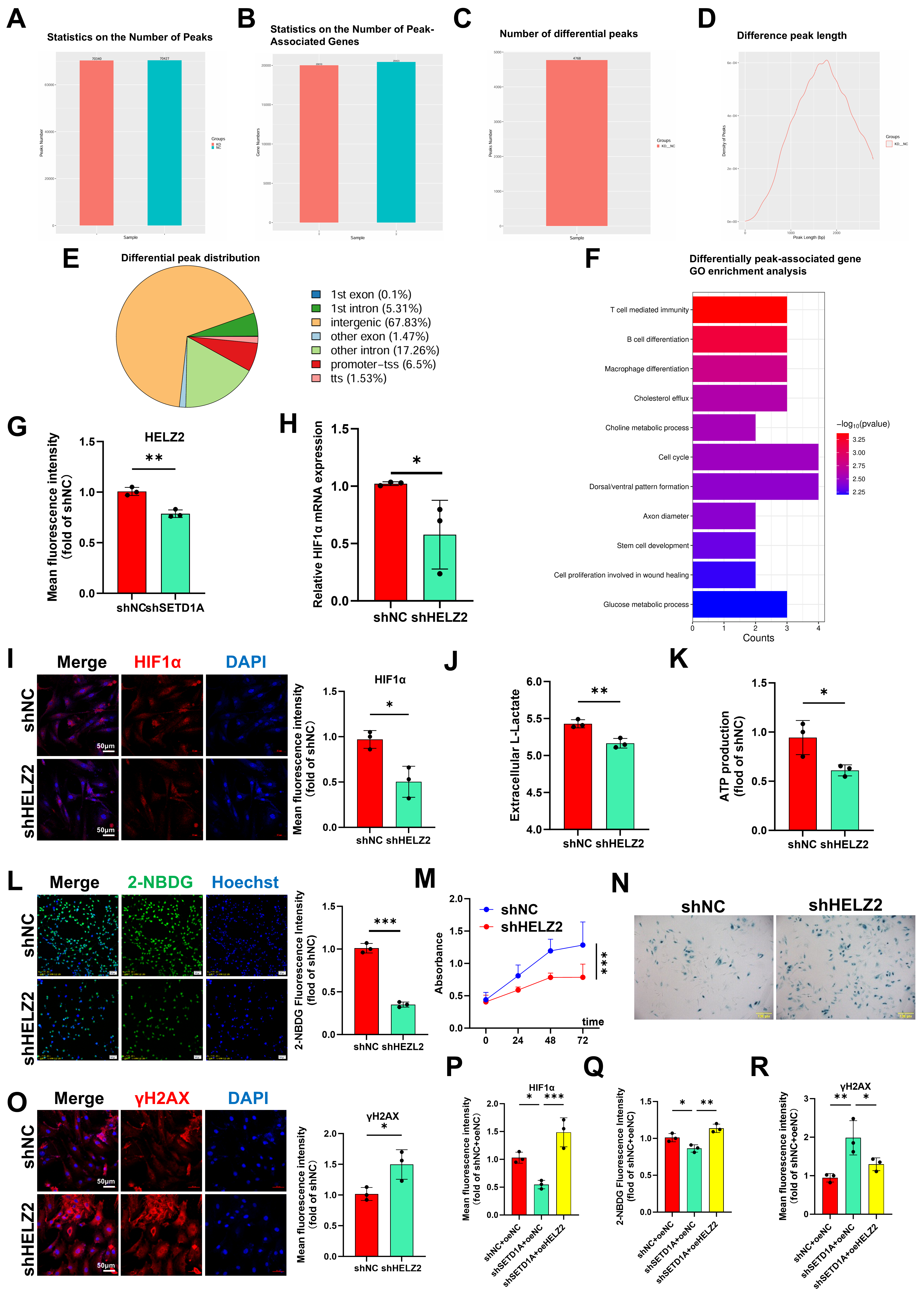
**

**Fig. S7. ChIP-seq analysis of SETD1A-regulated H3K4me3 peaks and validation of the effects of HELZ2 on NP cells.**

H3K4me3 ChIP-seq was performed on RNPCs transfected with shNC or shSETD1A. (A) Total number of identified H3K4me3 peaks. (B) Number of genes associated with H3K4me3 peaks. (C) Count of differentially H3K4me3 peaks. (D) Distribution of differential H3K4me3 peak lengths. (E) Genomic distribution of differential H3K4me3 peaks. (F) GO enrichment analysis of genes associated with differential H3K4me3 peaks. (G) Immunofluorescence quantification analysis of Fig. 7I (n = 3). (H) mRNA expression of HIF1α detected by qPCR in RNPCs (n = 3). (I) Representative immunofluorescence staining of HIF1α in RNPCs treated with different agents and quantification analysis(n=3). (J) Extracellular lactate levels in RNPCs from different groups (n = 3). (K) Intracellular ATP content of RNPCs from indicated groups (n = 3). (L) Glucose uptake capacity of RNPCs from indicated groups and quantification analysis(n=3). (M) RNPC proliferation analyzed by CCK-8 assay (n=3). (N) SA-β-Gal staining and quantification of SA-β-Gal-positive RNPCs. (O) Representative immunofluorescence staining of γH2AX in RNPCs treated with indicated agents and quantification analysis(n=3). (P) Immunofluorescence quantification analysis of Fig. 7L (n = 3). (Q) Immunofluorescence quantification analysis of Fig. 7O (n = 3). (R) Immunofluorescence quantification analysis of Fig. 7S (n = 3). **p* < 0.05, ***p* < 0.01, ****p* < 0.001, ns = not significant. (Unpaired t-test; one-way ANOVA; two-way ANOVA)

**
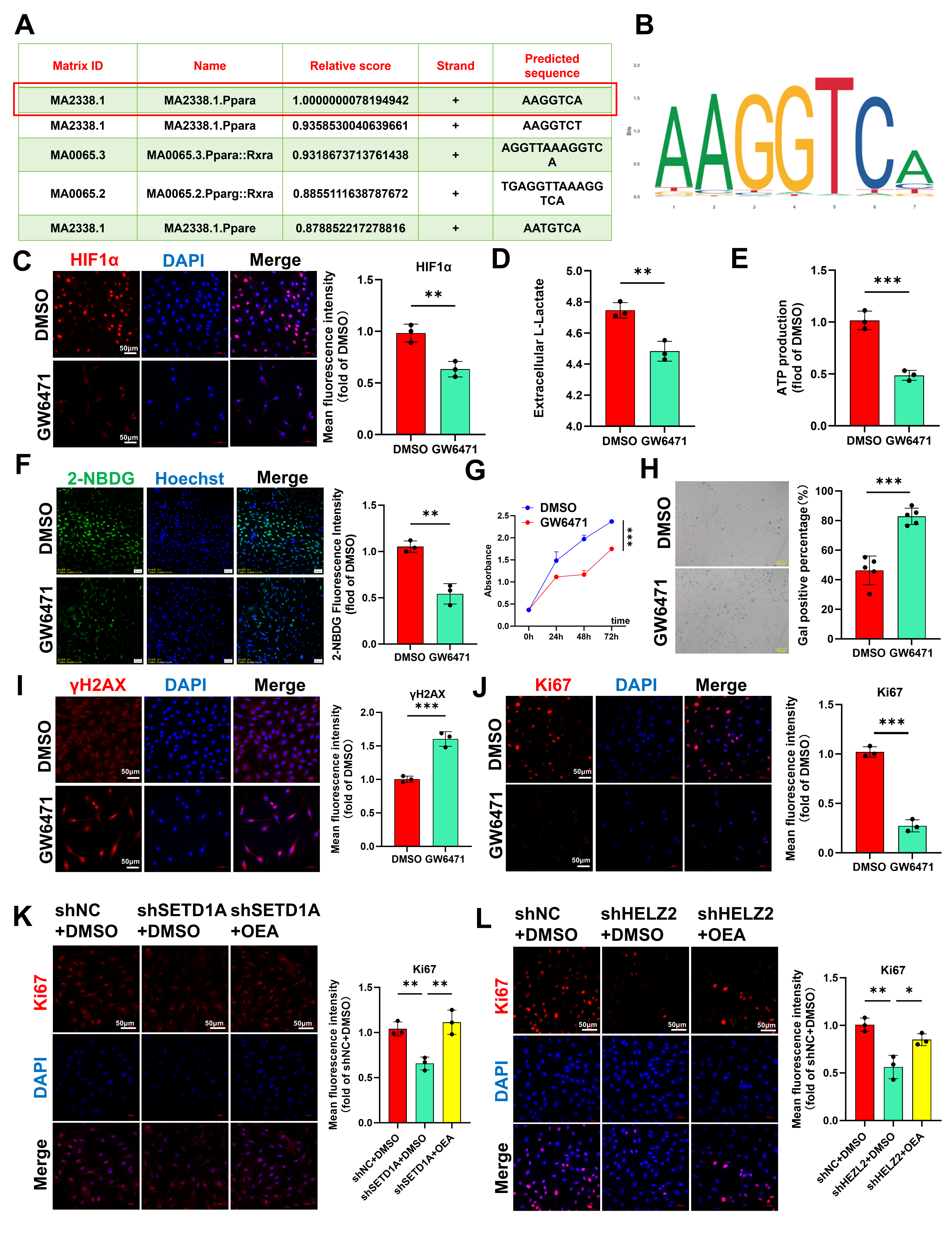
**

**Fig. S8. PPARα regulates HIF1α expression and glycolytic function of NP cells.**

(A) Prediction of the transcription factors of human HIF1α gene using the JASPAR database. (B) Binding motif of PPARα on the HIF1α promoter region. (C) Representative immunofluorescence staining of HIF1α in RNPCs treated with different agents and quantification analysis(n=3). (D) Extracellular lactate levels in RNPCs (n = 3). (E) Intracellular ATP content of RNPCs (n = 3). (F) Glucose uptake capacity of RNPCs. (G) RNPCs proliferation analyzed by CCK-8 assay (n = 3). (H) SA-β-Gal staining and quantification of SA-β-Gal–positive RNPCs. (I) Representative immunofluorescence staining of γH2AX in RNPCs treated with indicated agents and quantification analysis(n=3). (J–L) Representative immunofluorescence staining of Ki67 in RNPCs subjected to different treatments and quantification analysis(n=3).

**p* < 0.05, ***p* < 0.01, ****p* < 0.001, ns = not significant. (Unpaired t-test; one-way ANOVA; two-way ANOVA)

Original uncropped WB:

Fig.6 E-The first biological replication


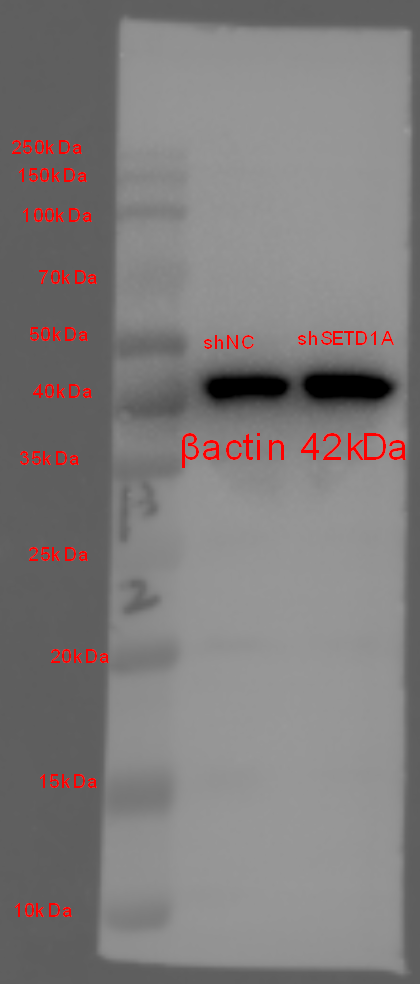

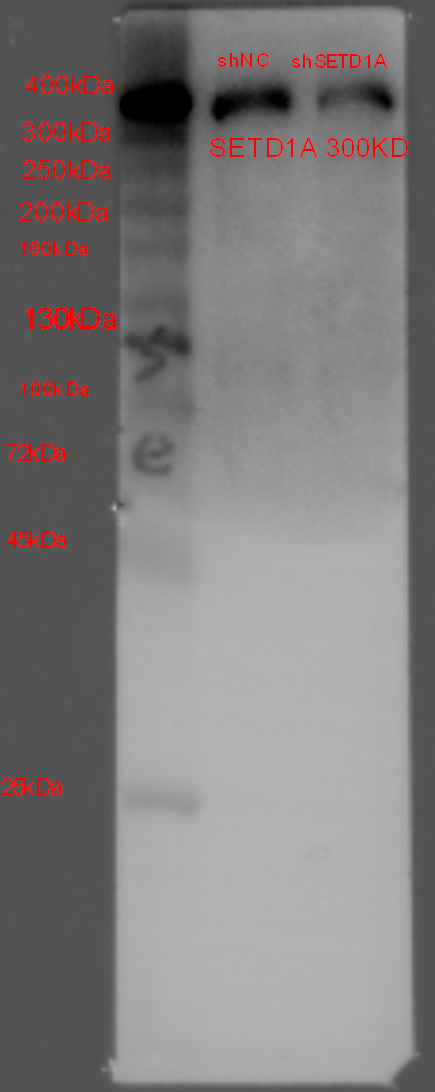

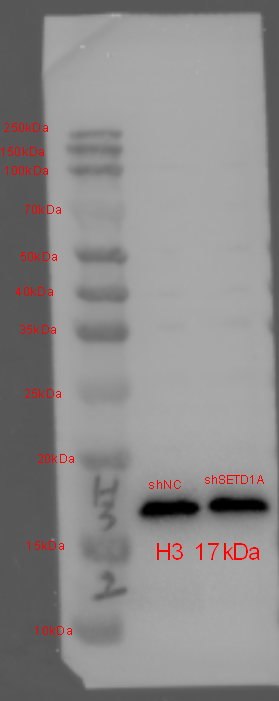

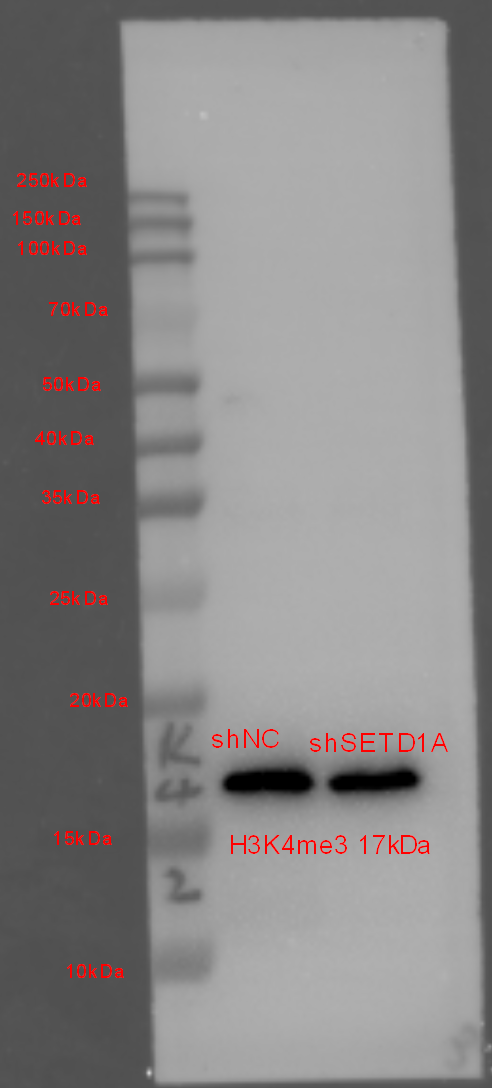

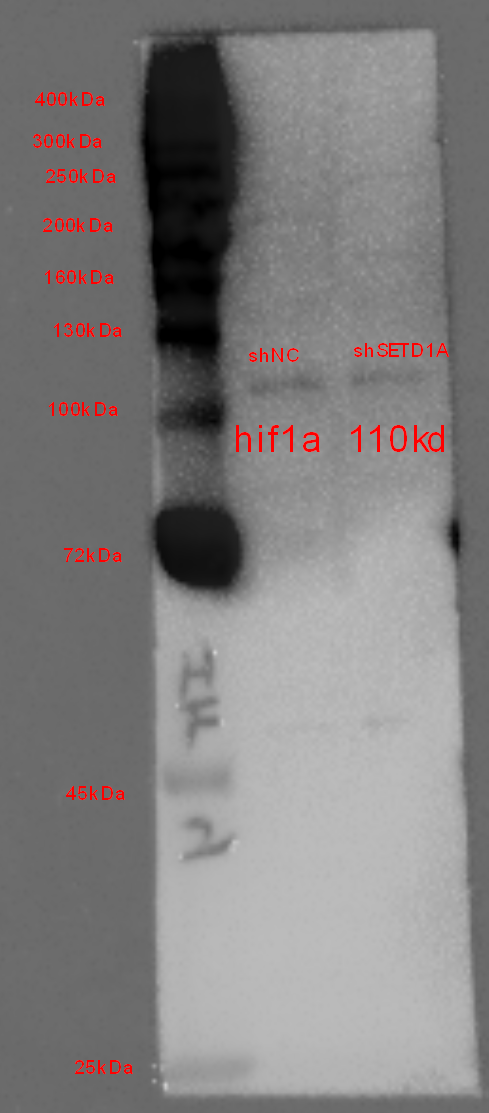


Fig.6 E-The second biological replicate


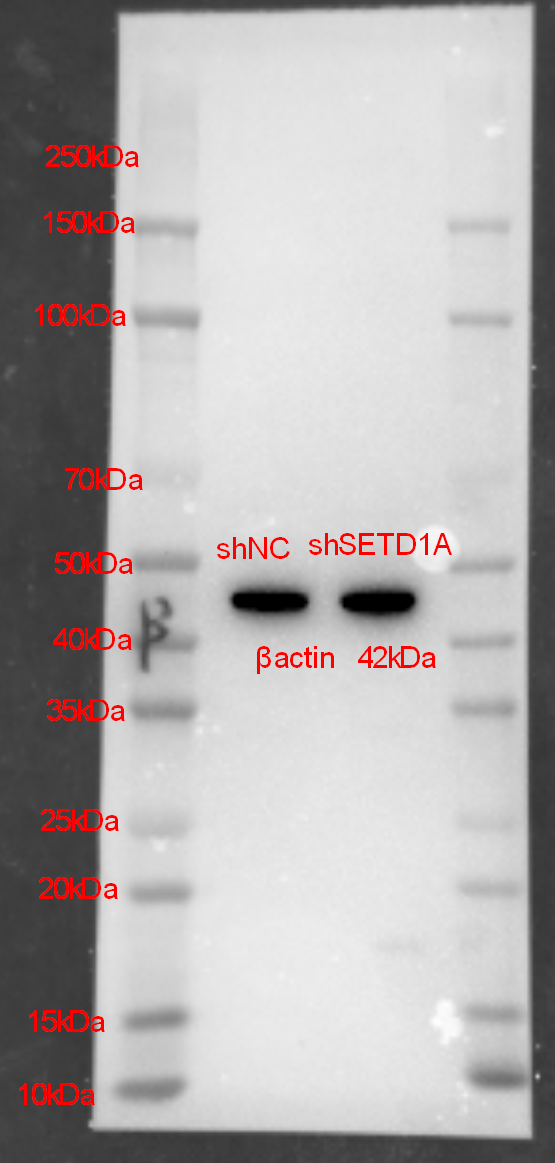

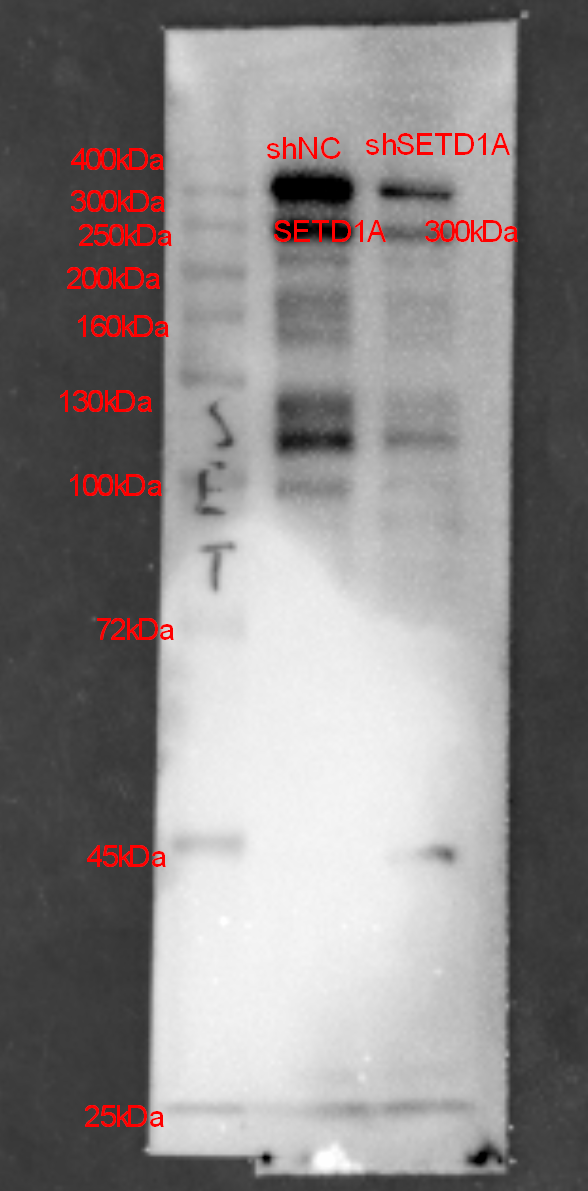

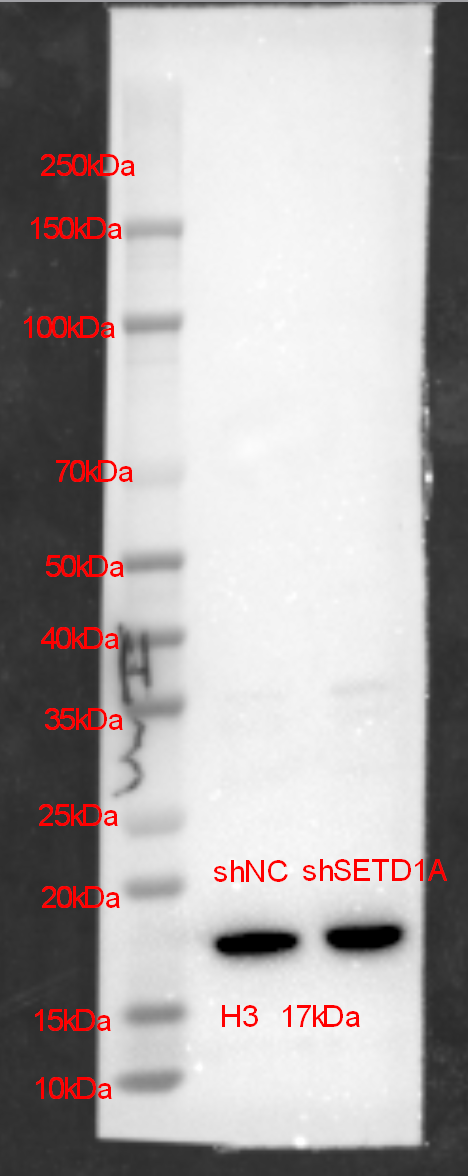

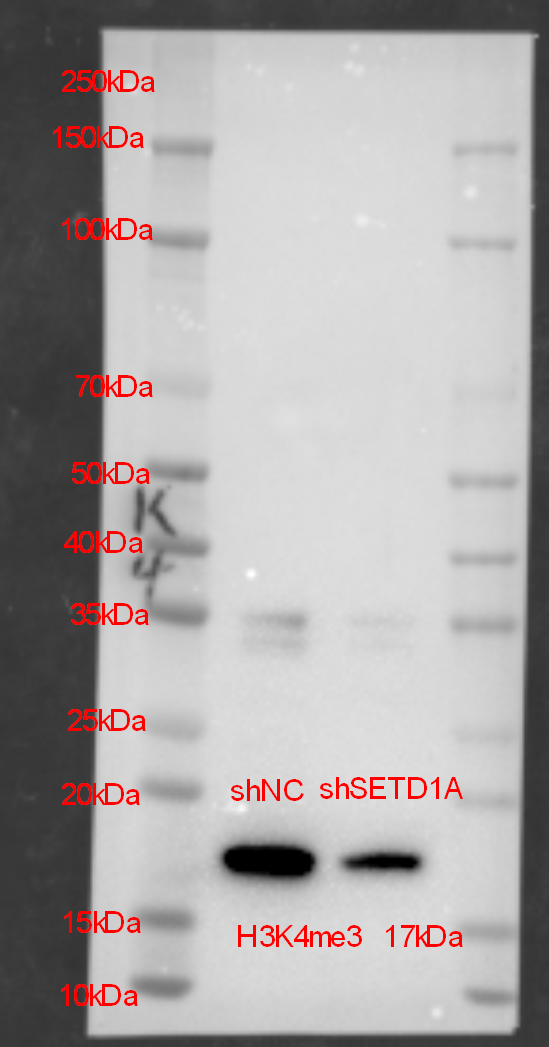

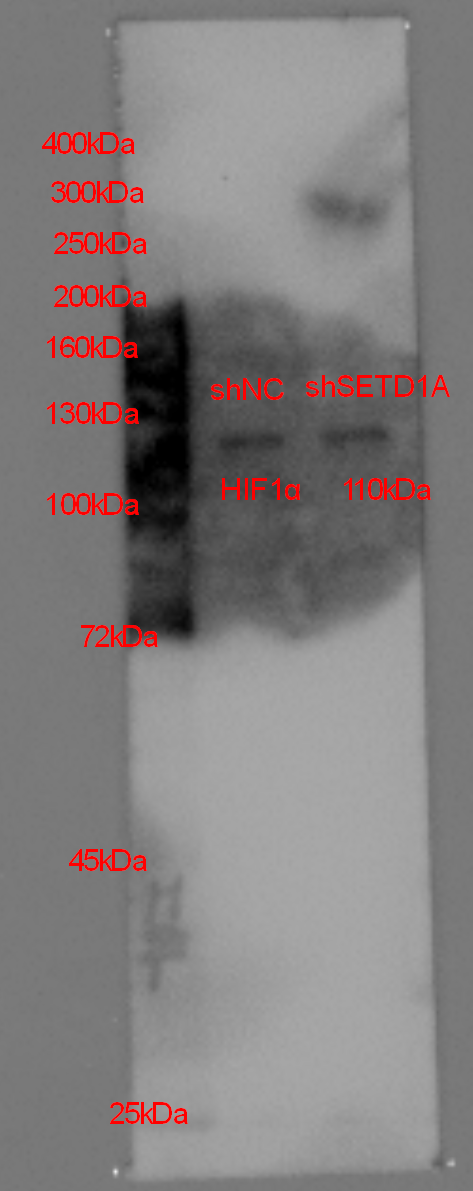


Fig.6 E-The third biological replicate


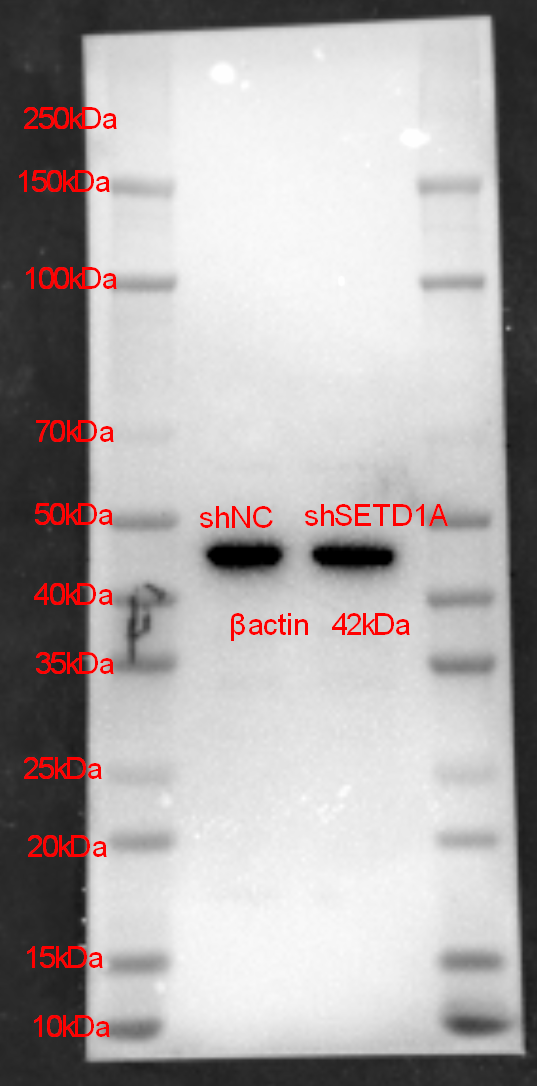

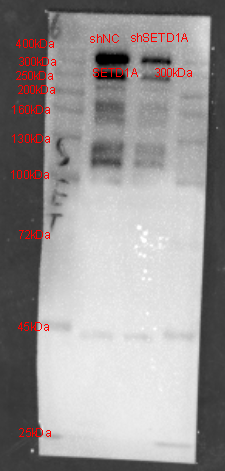

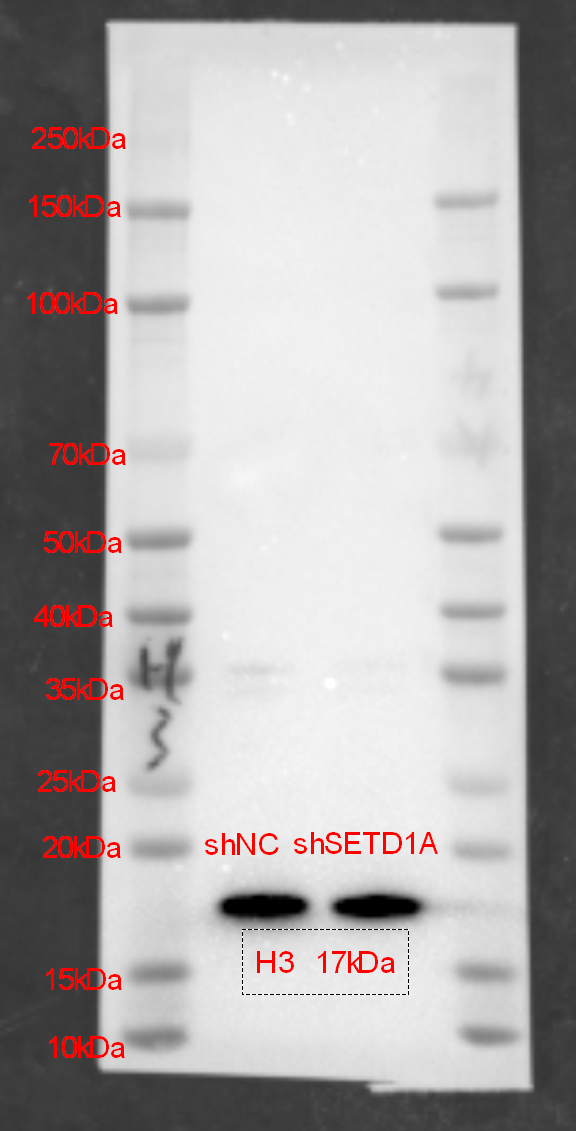

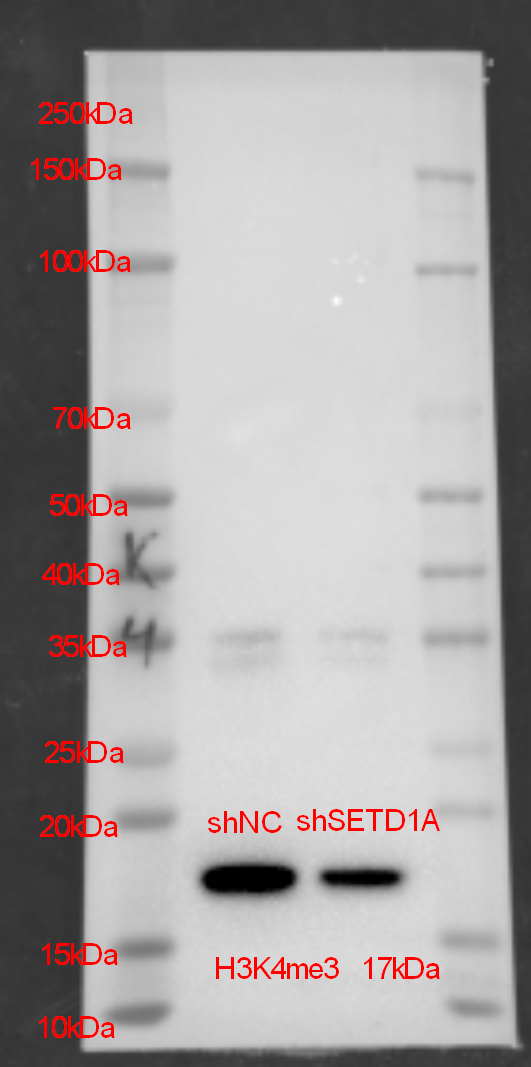

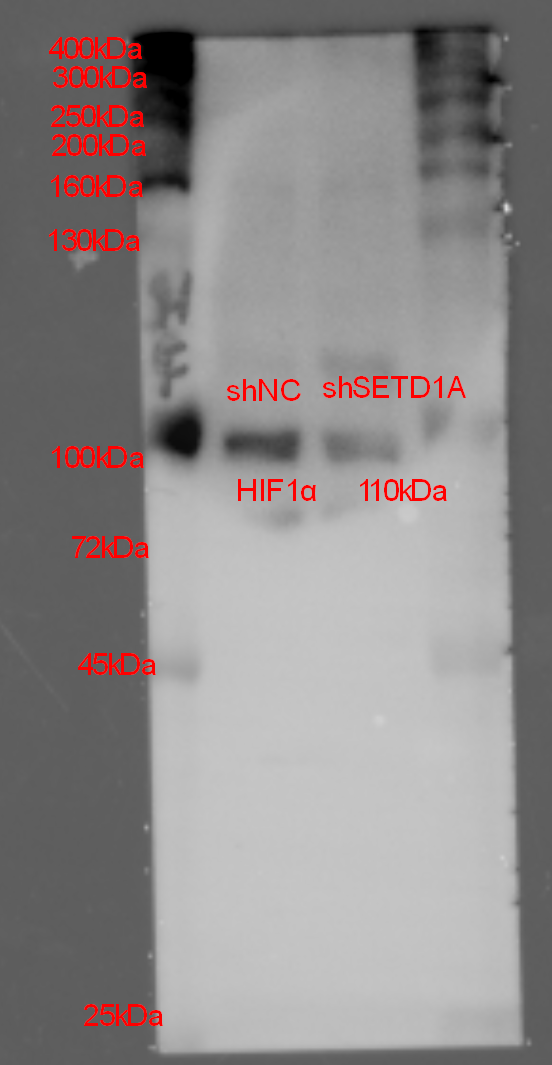


Fig.7 H-The first biological replication


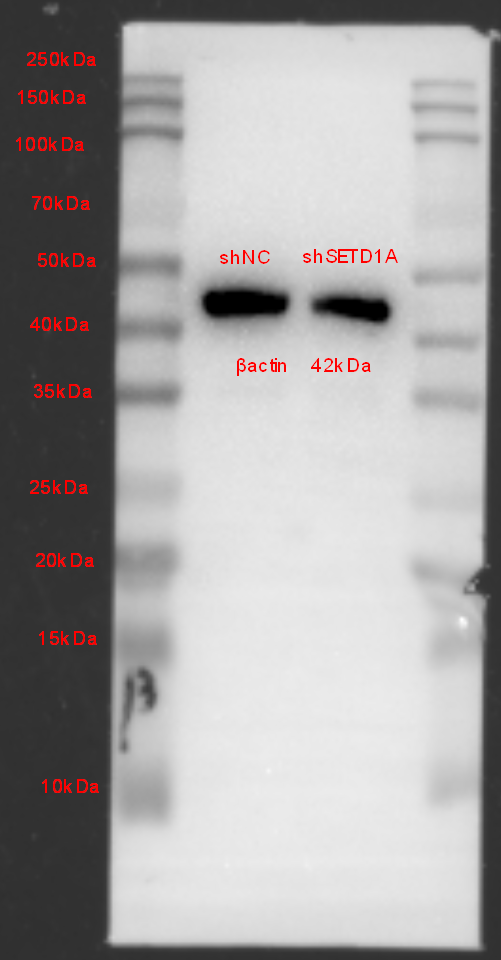

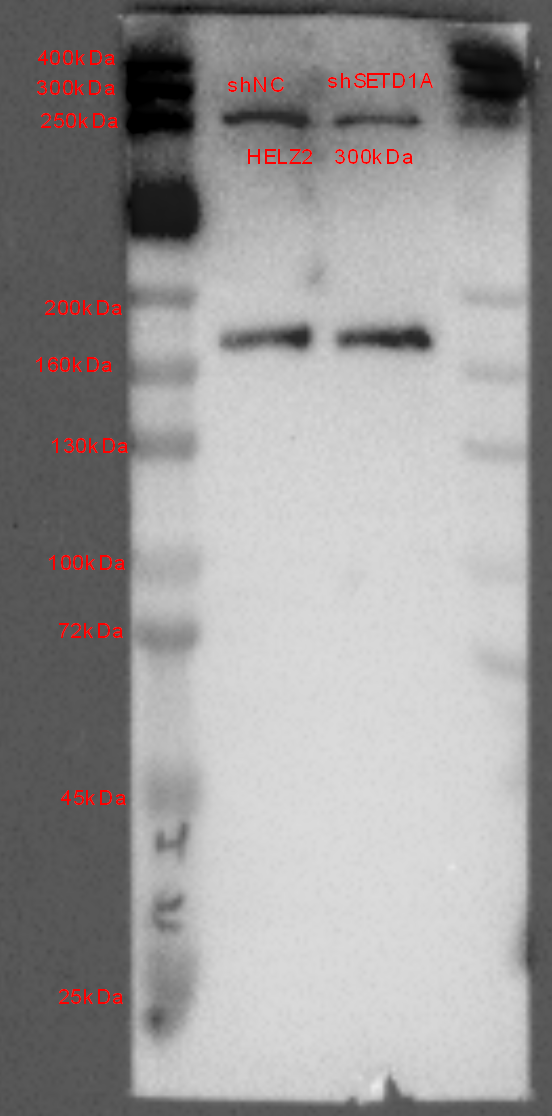


Fig.7 H-The second biological replicate


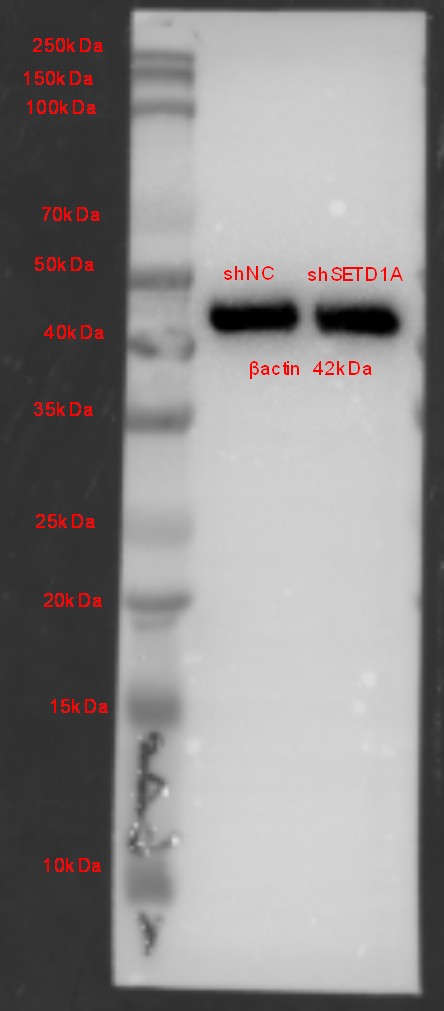

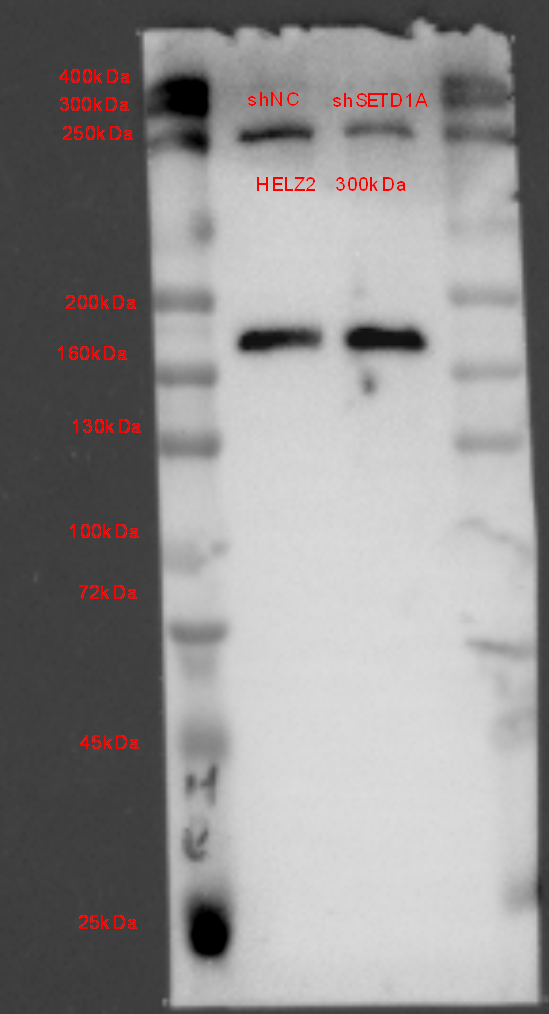


Fig.7 H-The third biological replicate


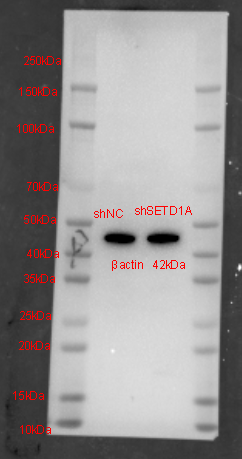

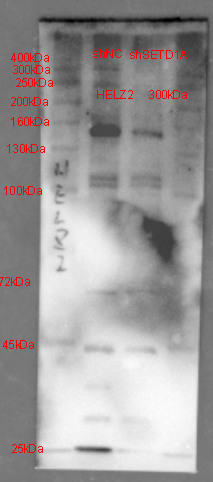


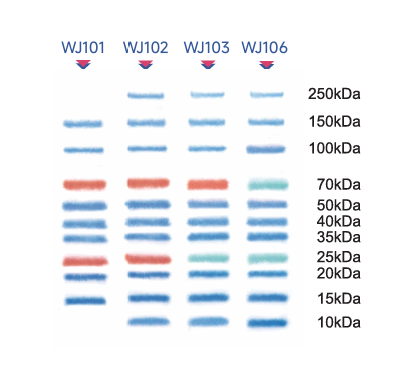


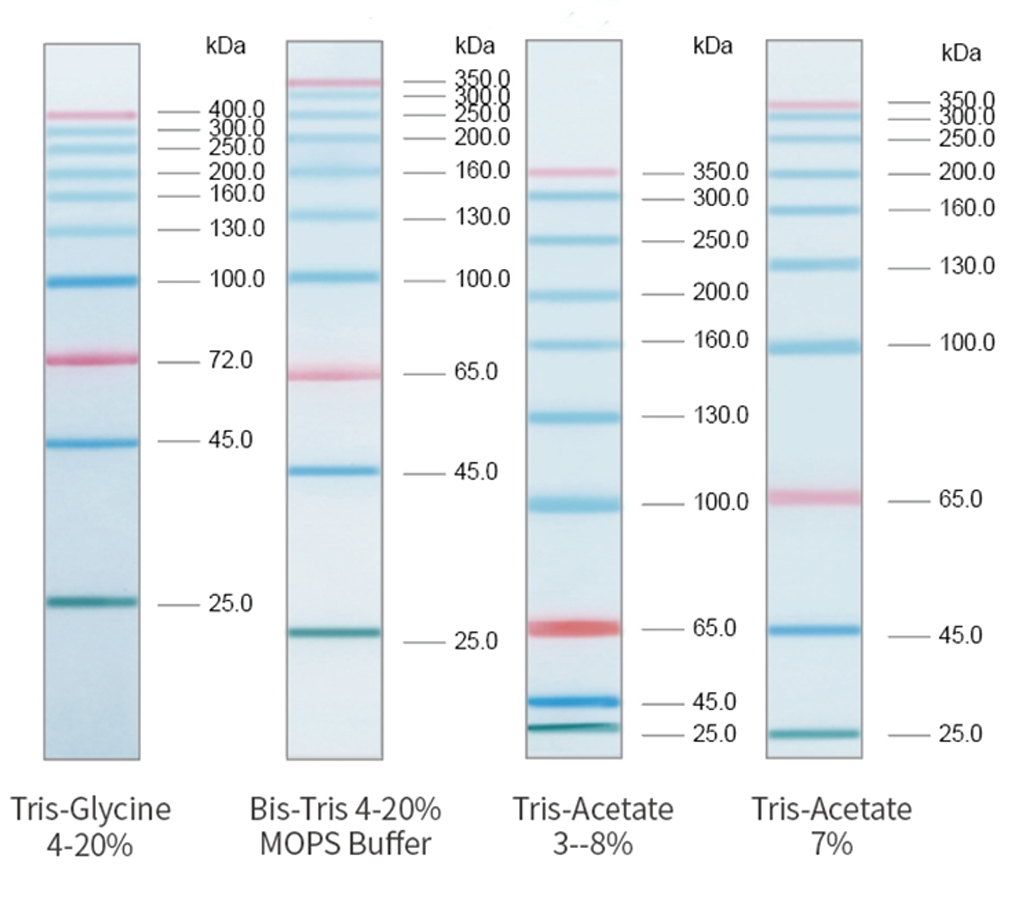


[1] L. Liu, Y. Zhang, J. Fu, X. Ai, D. Long, X. Leng, Y. Zhang, B. Huang, C. Li, Y. Zhou, C. Feng, Gli1 depletion induces oxidative stress and apoptosis of nucleus pulposus cells via Fos in intervertebral disc degeneration, J Orthop Translat. 40 (2023) 116-131. <https://doi.org/10.1016/j.jot.2023.05.008>

[2] S. Chen, J. Fu, J. Long, C. Liu, X. Ai, D. Long, X. Leng, Y. Zhang, Z. Liao, C. Li, Y. Zhou, S. Dong, B. Huang, C. Feng, Bulk RNA-seq conjoined with ScRNA-seq analysis reveals the molecular characteristics of nucleus pulposus cell ferroptosis in rat aging intervertebral discs, Arthritis Res. Ther. 27 (2025) 90. <https://doi.org/10.1186/s13075-025-03550-7>

[3] O. Wu, Y. Jin, Z. Zhang, H. Zhou, W. Xu, L. Chen, M. Jones, K.Y.H. Kwan, J. Gao, K. Zhang, X. Cheng, Q. Chen, X. Wang, Y.M. Li, Z. Guo, J. Sun, Z. Chen, B. Wang, X. Wang, S. Shen, A. Wu, KMT2A regulates the autophagy-GATA4 axis through METTL3-mediated m(6)A modification of ATG4a to promote NPCs senescence and IVDD progression, Bone Res. 12 (2024) 67. <https://doi.org/10.1038/s41413-024-00373-1>
