# Supplementary material for: SETD1A Regulates Glycolysis and Senescence of Nucleus Pulposus Cells via H3K4me3–HELZ2/PPARα‐HIF1α Axis to Drive Intervertebral Disc Degeneration
Source: Adv Sci (Weinh). 2026 Mar 31;13(34):e75105. doi: 10.1002/advs.75105 (PMC13285123; doi:10.1002/advs.75105)
Supplement: Supplementary file 2 — Supporting File 2: advs75105‐sup‐0002‐TableS1.docx. [file ADVS-13-e75105-s003.docx]

**Table S1. Primary antibodies used in this study.**

| **Antibody Name** | **Brand** | **Catalog Number** | **WB** | **immunofluorescence** |
| --- | --- | --- | --- | --- |
| H3K4me3 | Cell Signalling | C42D8 | 1:1000 | 1:200 |
| H3K4me3 | ABclonal | A2357 | 1:5000 | 1:200 |
| SETD1A | Abcam | AB70378 | 1:1000 | 1:200 |
| SETD1A | Cell Signalling | E3E2S | 1:1000 |  |
| γH2AX | Abcam | AB11175 |  | 1:200 |
| γH2AX | Abcam | AB20669 | 1:1000 |  |
| Ki67 | ABclonal | A20018 |  | 1:200 |
| Ki67 | Abcam | AB16667 | 1:500 |  |
| P21 | Santa | SC-6246 |  | 1:100 |
| P21 | Abcam | AB109199 | 1:1000 |  |
| CCND2 | ABclonal | A1773 |  | 1:200 |
| ACAN | Proteintech | 13880-1-AP |  | 1:200 |
| LDHA | ABclonal | A21893 |  | 1:200 |
| LDHA | Proteintech | 21799-1-AP |  | 1:200 |
| HIF1α | Abcam | AB1 | 1:1000 | 1:200 |
| HIF1α | ABclonal | A11945 | 1:1000 | 1:200 |
| HELZ2 | Invitrogen | PA5-101743 | 1:1000 | 1:200 |
| PPARα | Affinit | AF5301 |  | 1:200 |
| TGFβ1 | ABclonal | A2124 |  | 1:200 |
| Col1a1 | Santa | SC-52658 |  | 1:100 |
| Col2a1 | Proteintech | 66761-1-Ig |  | 1:100 |
| Col2a1 | arigobio | ARG20787 |  | 1:200 |
