# Supplementary material for: SETD1A Regulates Glycolysis and Senescence of Nucleus Pulposus Cells via H3K4me3–HELZ2/PPARα‐HIF1α Axis to Drive Intervertebral Disc Degeneration
Source: Adv Sci (Weinh). 2026 Mar 31;13(34):e75105. doi: 10.1002/advs.75105 (PMC13285123; doi:10.1002/advs.75105)
Supplement: Supplementary file 3 — Supporting File 3: advs75105‐sup‐0003‐TableS2.docx. [file ADVS-13-e75105-s004.docx]

**Table S2. Patient information.**

| Age | Gender | Diagnosis | Pfirrmann grades | | | | |
| --- | --- | --- | --- | --- | --- | --- | --- |
|  |  |  | L5/S1 | L4/5 | L3/4 | L2/3 | L1/2 |
| 15 | male | Lumbar Disc Herniation | 2 | 3 | 3 | 2 | 2 |
| 16 | male | Lumbar Disc Herniation | 3 | 2 | 2 | 2 | 1 |
| 19 | male | Lumbar Disc Herniation | 4 | 2 | 2 | 1 | 1 |
| 19 | male | Lumbar Disc Herniation | 4 | 3 | 2 | 2 | 3 |
| 21 | male | Lumbar Disc Herniation | 4 | 5 | 2 | 1 | 1 |
| 21 | female | Lumbar Disc Herniation | 4 | 2 | 1 | 2 | 2 |
| 22 | male | Lumbar Disc Herniation | 3 | 3 | 2 | 2 | 2 |
| 24 | male | Lumbar Disc Herniation | 2 | 3 | 2 | 1 | 1 |
| 24 | male | Lumbar spinal stenosis with disc herniation | 1 | 3 | 1 | 1 | 3 |
| 25 | male | Lumbar Disc Herniation | 2 | 2 | 1 | 1 | 2 |
| 25 | male | Lumbar spinal stenosis with disc herniation | 4 | 4 | 4 | 4 | 3 |
| 26 | male | Lumbar disc herniation with radiculopathy | 3 | 2 | 2 | 2 | 2 |
| 27 | male | Lumbar Disc Herniation | 2 | 5 | 2 | 2 | 1 |
| 27 | female | Lumbar spinal stenosis with disc herniation | 4 | 4 | 1 | 1 | 1 |
| 27 | male | Lumbar spinal stenosis with disc herniation | 4 | 4 | 2 | 1 | 2 |
| 27 | male | Lumbar spinal stenosis with disc herniation | 2 | 4 | 1 | 2 | 2 |
| 28 | male | Lumbar Disc Herniation | 2 | 4 | 2 | 3 | 2 |
| 28 | male | Lumbar spinal stenosis with disc herniation | 2 | 4 | 2 | 2 | 2 |
| 28 | male | Lumbar spinal stenosis with disc herniation | 2 | 3 | 3 | 1 | 1 |
| 29 | male | Lumbar Disc Herniation | 4 | 4 | 4 | 4 | 4 |
| 30 | male | Lumbar Disc Herniation | 4 | 2 | 2 | 2 | 2 |
| 31 | male | Lumbar Disc Herniation | 4 | 2 | 2 | 2 | 2 |
| 31 | male | Lumbar Disc Herniation | 4 | 3 | 2 | 2 | 2 |
| 32 | male | Lumbar Disc Herniation | 4 | 3 | 3 | 3 | 3 |
| 32 | male | Lumbar Disc Herniation | 2 | 4 | 4 | 2 | 2 |
| 33 | male | Lumbar disc herniation with radiculopathy | 2 | 3 | 1 | 2 | 2 |
| 33 | male | Lumbar disc herniation with radiculopathy | 4 | 3 | 2 | 2 | 2 |
| 34 | male | Lumbar disc herniation with radiculopathy | 4 | 4 | 2 | 2 | 2 |
| 34 | male | Lumbar Disc Herniation | 5 | 4 | 2 | 2 | 2 |
| 34 | male | Lumbar Disc Herniation | 2 | 4 | 2 | 2 | 2 |
| 34 | male | Lumbar spinal stenosis with disc herniation | 3 | 3 | 3 | 1 | 1 |
| 36 | male | Lumbar Disc Herniation | 4 | 4 | 3 | 2 | 2 |
| 36 | female | Lumbar Disc Herniation | 4 | 4 | 3 | 2 | 2 |
| 36 | female | Lumbar Disc Herniation | 4 | 4 | 2 | 2 | 2 |
| 36 | female | Lumbar spinal stenosis with disc herniation | 5 | 2 | 2 | 2 | 1 |
| 37 | male | Lumbar spinal stenosis with disc herniation | 2 | 3 | 3 | 4 | 2 |
| 37 | male | Lumbar spinal stenosis with disc herniation | 3 | 3 | 2 | 2 | 2 |
| 37 | male | Lumbar Disc Herniation | 4 | 2 | 2 | 2 | 2 |
| 37 | female | Lumbar Disc Herniation | 4 | 4 | 3 | 2 | 2 |
| 38 | male | Lumbar spinal stenosis with disc herniation | 4 | 4 | 3 | 2 | 2 |
| 39 | male | Lumbar Disc Herniation | 2 | 3 | 3 | 2 | 3 |
| 40 | male | Lumbar Disc Herniation | 4 | 4 | 3 | 2 | 2 |
| 40 | female | Lumbar Disc Herniation | 4 | 3 | 3 | 2 | 1 |
| 40 | female | Lumbar spinal stenosis with disc herniation | 4 | 5 | 4 | 3 | 1 |
| 41 | female | Lumbar Disc Herniation | 4 | 4 | 3 | 2 | 1 |
| 41 | male | Lumbar spinal stenosis with disc herniation | 3 | 3 | 2 | 3 | 3 |
| 42 | female | Lumbar disc herniation with radiculopathy | 5 | 4 | 5 | 2 | 2 |
| 42 | male | Lumbar spinal stenosis with disc herniation | 2 | 3 | 2 | 4 | 2 |
| 43 | female | Lumbar disc herniation with radiculopathy | 2 | 5 | 4 | 2 | 2 |
| 43 | male | Lumbar Disc Herniation | 5 | 4 | 3 | 3 | 2 |
| 43 | male | Lumbar spinal stenosis with disc herniation | 3 | 4 | 3 | 2 | 2 |
| 44 | male | Lumbar Disc Herniation | 4 | 4 | 2 | 3 | 2 |
| 44 | male | Lumbar disc herniation with radiculopathy | 5 | 2 | 2 | 2 | 2 |
| 44 | male | Lumbar Disc Herniation | 4 | 4 | 4 | 3 | 3 |
| 44 | female | Lumbar Disc Herniation | 4 | 4 | 4 | 3 | 3 |
| 45 | male | Lumbar Disc Herniation | 3 | 3 | 2 | 1 | 2 |
| 45 | female | Lumbar Disc Herniation | 4 | 4 | 3 | 4 | 5 |
| 46 | female | Lumbar spinal stenosis with disc herniation | 2 | 5 | 4 | 3 | 3 |
| 46 | female | Lumbar Disc Herniation | 2 | 3 | 3 | 2 | 1 |
| 46 | female | Lumbar disc herniation with radiculopathy | 2 | 4 | 2 | 1 | 1 |
| 46 | male | Lumbar Disc Herniation | 4 | 3 | 2 | 2 | 2 |
| 47 | male | Lumbar Disc Herniation | 4 | 4 | 3 | 2 | 2 |
| 47 | female | Lumbar Disc Herniation | 4 | 2 | 3 | 2 | 1 |
| 48 | male | Lumbar Disc Herniation | 3 | 3 | 2 | 2 | 2 |
| 49 | male | Lumbar Disc Herniation | 5 | 4 | 4 | 4 | 2 |
| 49 | male | Lumbar disc herniation with radiculopathy | 4 | 4 | 3 | 3 | 3 |
| 49 | female | Lumbar Disc Herniation | 3 | 5 | 4 | 3 | 1 |
| 49 | female | Lumbar spinal stenosis with disc herniation | 3 | 4 | 2 | 1 | 1 |
| 50 | female | Lumbar disc herniation with radiculopathy | 4 | 5 | 3 | 2 | 2 |
| 50 | male | Lumbar Disc Herniation | 4 | 3 | 4 | 3 | 2 |
| 51 | female | Lumbar disc herniation with radiculopathy | 3 | 4 | 2 | 2 | 2 |
| 51 | female | Lumbar disc herniation with radiculopathy | 2 | 4 | 4 | 2 | 2 |
| 51 | female | Lumbar Disc Herniation | 3 | 4 | 3 | 2 | 2 |
| 51 | female | Lumbar spinal stenosis with disc herniation | 2 | 3 | 3 | 2 | 2 |
| 51 | female | Lumbar spinal stenosis with disc herniation | 5 | 3 | 3 | 3 | 3 |
| 52 | female | Lumbar Disc Herniation | 2 | 3 | 3 | 2 | 1 |
| 52 | male | Lumbar disc herniation with radiculopathy | 2 | 3 | 3 | 2 | 2 |
| 53 | female | Lumbar spinal stenosis with disc herniation | 5 | 4 | 2 | 2 | 2 |
| 54 | male | Lumbar spinal stenosis with disc herniation | 4 | 4 | 4 | 4 | 3 |
| 54 | male | Lumbar Disc Herniation | 4 | 3 | 2 | 3 | 2 |
| 54 | female | Lumbar Disc Herniation | 5 | 2 | 2 | 3 | 3 |
| 55 | male | Lumbar Disc Herniation | 4 | 4 | 3 | 2 | 2 |
| 55 | male | Lumbar Disc Herniation | 4 | 3 | 2 | 2 | 2 |
| 55 | male | Lumbar Disc Herniation | 2 | 3 | 4 | 2 | 4 |
| 55 | female | Lumbar Disc Herniation | 2 | 4 | 3 | 3 | 2 |
| 56 | male | Lumbar spinal stenosis with disc herniation | 3 | 3 | 2 | 2 | 2 |
| 56 | male | Lumbar spinal stenosis with disc herniation | 5 | 4 | 3 | 2 | 1 |
| 56 | female | Lumbar disc herniation, lumbar spondylolisthesis | 5 | 4 | 3 | 4 | 4 |
| 56 | female | Lumbar spinal stenosis with disc herniation | 2 | 3 | 2 | 2 | 3 |
| 58 | female | Lumbar spinal stenosis | 3 | 3 | 3 | 2 | 1 |
| 58 | female | Lumbar disc herniation with radiculopathy | 2 | 4 | 3 | 3 | 3 |
| 58 | male | Lumbar Disc Herniation | 3 | 3 | 3 | 4 | 2 |
| 58 | male | Lumbar Disc Herniation | 4 | 3 | 3 | 2 | 2 |
| 58 | female | Lumbar Disc Herniation | 4 | 3 | 3 | 2 | 2 |
| 58 | female | Lumbar Disc Herniation | 4 | 3 | 3 | 4 | 2 |
| 58 | male | Lumbar Disc Herniation | 3 | 3 | 3 | 3 | 2 |
| 58 | male | Lumbar spinal stenosis with disc herniation | 4 | 4 | 2 | 2 | 2 |
| 59 | male | Lumbar spinal stenosis | 3 | 5 | 3 | 3 | 2 |
| 59 | female | Lumbar Disc Herniation | 3 | 2 | 3 | 4 | 3 |
| 60 | male | Lumbar disc herniation with radiculopathy | 3 | 4 | 2 | 2 | 3 |
| 60 | female | Lumbar Disc Herniation | 2 | 4 | 3 | 3 | 3 |
| 61 | male | Lumbar spinal stenosis with disc herniation | 3 | 3 | 2 | 4 | 2 |
| 61 | male | Lumbar spinal stenosis with disc herniation | 4 | 3 | 3 | 2 | 1 |
| 62 | male | Lumbar Disc Herniation | 3 | 3 | 2 | 2 | 2 |
| 63 | male | Lumbar spinal stenosis | 3 | 3 | 1 | 2 | 2 |
| 64 | female | Lumbar spinal stenosis with disc herniation | 3 | 4 | 3 | 4 | 2 |
| 65 | male | Lumbar disc herniation with radiculopathy | 3 | 3 | 5 | 5 | 5 |
| 65 | female | Lumbar Disc Herniation | 4 | 2 | 3 | 3 | 2 |
| 66 | male | Lumbar Disc Herniation | 4 | 5 | 4 | 3 | 4 |
| 69 | female | Lumbar disc herniation with radiculopathy | 3 | 5 | 3 | 1 | 2 |
| 70 | male | Lumbar Disc Herniation | 5 | 4 | 4 | 4 | 3 |
| 70 | female | Lumbar Disc Herniation | 3 | 2 | 3 | 3 | 2 |
| 71 | male | Lumbar spinal stenosis with disc herniation | 3 | 3 | 3 | 2 | 2 |
| 72 | female | Lumbar Disc Herniation | 2 | 4 | 4 | 2 | 3 |
| 74 | female | Lumbar Disc Herniation | 3 | 4 | 3 | 3 | 2 |
| 76 | male | Lumbar spinal stenosis with disc herniation | 4 | 4 | 3 | 3 | 3 |
| 77 | male | Lumbar Disc Herniation | 2 | 3 | 3 | 3 | 3 |
| 78 | male | Lumbar spinal stenosis with disc herniation | 2 | 4 | 3 | 4 | 2 |
| 81 | female | Lumbar spinal stenosis with disc herniation | 4 | 4 | 4 | 3 | 2 |
| 81 | female | Lumbar spinal stenosis | 5 | 3 | 3 | 3 | 4 |
| 82 | male | Lumbar disc herniation, lumbar spondylolisthesis | 3 | 4 | 5 | 5 | 3 |
