# Supplementary material for: SETD1A Regulates Glycolysis and Senescence of Nucleus Pulposus Cells via H3K4me3–HELZ2/PPARα‐HIF1α Axis to Drive Intervertebral Disc Degeneration
Source: Adv Sci (Weinh). 2026 Mar 31;13(34):e75105. doi: 10.1002/advs.75105 (PMC13285123; doi:10.1002/advs.75105)
Supplement: Supplementary file 4 — Supporting File 4: advs75105‐sup‐0004‐TableS3.docx. [file ADVS-13-e75105-s005.docx]

**Table S3. Patient information.**

| Age | Gender | Diagnosis | Surgical segment | H3K4me3(Positive rate) |
| --- | --- | --- | --- | --- |
| 15 | male | Lumbar Disc Herniation | L4/5 | 0.739130435 |
| 16 | male | Lumbar Disc Herniation | L3/4 | 0.75 |
| 19 | male | Lumbar Disc Herniation | L5/S1 | 0.545454545 |
| 19 | male | Lumbar Disc Herniation | L4/5 | 0.8 |
| 21 | male | Lumbar Disc Herniation | L4/5 | 0.9375 |
| 21 | female | Lumbar disc herniation with radiculopathy | L5/S1 | 0.642857143 |
| 24 | male | Lumbar spinal stenosis with disc herniation | L4/5 | 0.875 |
| 25 | male | Lumbar Disc Herniation | L5/S1 | 0.7 |
| 25 | male | Lumbar Disc Herniation | L5/S1 | 0.846153846 |
| 25 | male | Lumbar spinal stenosis with disc herniation | L3/4、L5/S1 | 0.625 |
| 27 | male | Lumbar Disc Herniation | L4/5 | 0.615384615 |
| 27 | female | Lumbar spinal stenosis with disc herniation | L4/5、L5/S1 | 0.303030303 |
| 27 | male | Lumbar spinal stenosis with disc herniation | L5/S1 | 0.6875 |
| 27 | male | Lumbar spinal stenosis with disc herniation | L4/5 | 0.736842105 |
| 28 | male | Lumbar spinal stenosis with disc herniation | L4/5 | 0.451612903 |
| 28 | male | Lumbar spinal stenosis with disc herniation | L3/4 | 0.6 |
| 31 | male | Lumbar Disc Herniation | L5/S1 | 0.8 |
| 32 | male | Lumbar disc herniation with radiculopathy | L4/5 | 0.75 |
| 32 | male | Lumbar Disc Herniation | L5/S1 | 0.5625 |
| 32 | male | Lumbar Disc Herniation | L5/S1 | 0.85 |
| 33 | male | Lumbar disc herniation with radiculopathy | L4/5 | 0.5 |
| 34 | male | Lumbar Disc Herniation | L5/S1 | 0.380952381 |
| 34 | male | Lumbar Disc Herniation | L4/5 | 0.6 |
| 34 | male | Lumbar spinal stenosis with disc herniation | L5/S1 | 0.52 |
| 35 | female | Lumbar disc herniation with radiculopathy | L4/5 | 0.555555556 |
| 36 | female | Lumbar spinal stenosis with disc herniation | L4/5 | 0.4375 |
| 37 | male | Lumbar spinal stenosis with disc herniation | L4/5 | 0.523809524 |
| 37 | male | Lumbar spinal stenosis with disc herniation | L3/4、4/5 | 0.647058824 |
| 38 | female | Lumbar spinal stenosis with disc herniation | L5/S1 | 0.461538462 |
| 38 | male | Lumbar Disc Herniation | L4/5 | 0.571428571 |
| 39 | male | Lumbar Disc Herniation | L4/5 | 0.3 |
| 40 | male | Lumbar Disc Herniation | L5/S1 | 0.3 |
| 40 | female | Lumbar Disc Herniation | L5/S1 | 0.444444444 |
| 40 | female | Lumbar Disc Herniation | L4/5 | 0.259259259 |
| 41 | female | Lumbar Disc Herniation | L5/S1 | 0.658536585 |
| 41 | male | Lumbar spinal stenosis with disc herniation | L4/5 | 0.533333333 |
| 43 | female | Lumbar disc herniation with radiculopathy | L5/S1 | 0.214285714 |
| 43 | male | Lumbar spinal stenosis with disc herniation | L4/5 | 0.625 |
| 44 | male | Lumbar Disc Herniation | L4/5 | 0.421052632 |
| 45 | female | Lumbar Disc Herniation | L4/5 | 0.24137931 |
| 47 | male | Lumbar Disc Herniation | L5/S1 | 0.625 |
| 49 | male | Lumbar Disc Herniation | L4/5 | 0.357142857 |
| 49 | female | Lumbar spinal stenosis with disc herniation | L4/5 | 0.545454545 |
| 50 | female | Lumbar disc herniation with radiculopathy | L4/5 | 0.48 |
| 50 | male | Lumbar Disc Herniation | L4/5 | 0.166666667 |
| 51 | female | Lumbar spinal stenosis with disc herniation | L4/5 | 0.4 |
| 53 | male | Lumbar disc herniation with radiculopathy | L5/S1 | 0.566666667 |
| 53 | female | Lumbar spinal stenosis with disc herniation | L4/5 | 0.1 |
| 54 | male | Lumbar spinal stenosis with disc herniation | L4/5 | 0.3 |
| 54 | male | Lumbar Disc Herniation | L5/S1 | 0.214285714 |
| 55 | male | Lumbar Disc Herniation | L5/S1 | 0.487179487 |
| 55 | female | Lumbar Disc Herniation | L4/5 | 0.055555556 |
| 56 | male | Lumbar spinal stenosis with disc herniation | L4/5 | 0.5 |
| 56 | female | Lumbar disc herniation, lumbar spondylolisthesis | L3/4 | 0.176923077 |
| 58 | female | Lumbar spinal stenosis | L4/5 | 0.5 |
| 58 | male | Lumbar Disc Herniation | L4/5 | 0.105263158 |
| 58 | female | Lumbar Disc Herniation | L4/5 | 0.133333333 |
| 58 | male | Lumbar spinal stenosis with disc herniation | L4/5 | 0.333333333 |
| 59 | male | Lumbar spinal stenosis with disc herniation | L4/5、L5/S1 | 0.481481481 |
| 60 | male | Lumbar disc herniation with radiculopathy | L4/5 | 0.1 |
| 61 | male | Lumbar spinal stenosis with disc herniation | L4/5 | 0.472222222 |
| 62 | male | Lumbar Disc Herniation | L4/5 | 0.333333333 |
| 64 | female | Lumbar spinal stenosis with disc herniation | L4/5 | 0.303030303 |
| 65 | female | Lumbar Disc Herniation | L5/S1 | 0.208333333 |
| 66 | male | Lumbar Disc Herniation | L5/S1 | 0.333333333 |
| 71 | male | Lumbar spinal stenosis with disc herniation | L3/4 | 0.285714286 |
| 74 | female | Lumbar Disc Herniation | L5/S1 | 0.214285714 |
| 76 | male | Lumbar spinal stenosis with disc herniation | L4/5 | 0.363636364 |
| 78 | male | Lumbar spinal stenosis with disc herniation | L4/5 | 0.105263158 |
| 81 | female | Lumbar spinal stenosis with disc herniation | L4/5 | 0.266666667 |
| 81 | female | Lumbar spinal stenosis | L4/5 | 0.349206349 |
| 82 | male | Lumbar disc herniation, lumbar spondylolisthesis | L5/S1 | 0.32 |
| Age | Gender | Diagnosis | Surgical segment | SETD1A |
| 15 | male | Lumbar Disc Herniation | L4/5 | 0.760869565 |
| 16 | male | Lumbar Disc Herniation | L3/4 | 1 |
| 19 | male | Lumbar Disc Herniation | L5/S1 | 0.565217391 |
| 19 | male | Lumbar Disc Herniation | L4/5 | 0.8 |
| 21 | male | Lumbar Disc Herniation | L4/5 | 0.717948718 |
| 21 | female | Lumbar disc herniation with radiculopathy | L5/S1 | 0.642857143 |
| 24 | male | Lumbar spinal stenosis with disc herniation | L4/5 | 0.782608696 |
| 25 | male | Lumbar Disc Herniation | L5/S1 | 0.769230769 |
| 25 | male | Lumbar Disc Herniation | L5/S1 | 0.833333333 |
| 25 | male | Lumbar spinal stenosis with disc herniation | L3/4、L5/S1 | 0.6875 |
| 27 | male | Lumbar Disc Herniation | L4/5 | 0.866666667 |
| 27 | male | Lumbar spinal stenosis with disc herniation | L5/S1 | 0.6875 |
| 27 | male | Lumbar spinal stenosis with disc herniation | L4/5 | 0.789473684 |
| 27 | female | Lumbar spinal stenosis with disc herniation | L4/5、L5/S1 | 0.333333333 |
| 28 | male | Lumbar spinal stenosis with disc herniation | L4/5 | 0.35483871 |
| 28 | male | Lumbar spinal stenosis with disc herniation | L3/4 | 0.55 |
| 31 | male | Lumbar Disc Herniation | L5/S1 | 0.65 |
| 32 | male | Lumbar disc herniation with radiculopathy | L4/5 | 0.75 |
| 32 | male | Lumbar Disc Herniation | L5/S1 | 0.75 |
| 32 | male | Lumbar Disc Herniation | L5/S1 | 0.85 |
| 33 | male | Lumbar disc herniation with radiculopathy | L4/5 | 0.535714286 |
| 34 | male | Lumbar Disc Herniation | L5/S1 | 0.458333333 |
| 34 | male | Lumbar Disc Herniation | L4/5 | 0.6 |
| 34 | male | Lumbar spinal stenosis with disc herniation | L5/S1 | 0.5 |
| 35 | female | Lumbar disc herniation with radiculopathy | L4/5 | 0.555555556 |
| 36 | female | Lumbar spinal stenosis with disc herniation | L4/5 | 0.3 |
| 37 | male | Lumbar spinal stenosis with disc herniation | L3/4、4/5 | 0.227272727 |
| 37 | male | Lumbar spinal stenosis with disc herniation | L4/5 | 0.428571429 |
| 38 | male | Lumbar Disc Herniation | L4/5 | 0.571428571 |
| 38 | female | Lumbar spinal stenosis with disc herniation | L5/S1 | 0.384615385 |
| 39 | male | Lumbar Disc Herniation | L4/5 | 0.4 |
| 40 | male | Lumbar Disc Herniation | L5/S1 | 0.0625 |
| 40 | female | Lumbar Disc Herniation | L5/S1 | 0.304347826 |
| 40 | female | Lumbar Disc Herniation | L4/5 | 0.208333333 |
| 41 | male | Lumbar spinal stenosis with disc herniation | L4/5 | 0.666666667 |
| 41 | female | Lumbar Disc Herniation | L5/S1 | 0.384615385 |
| 43 | male | Lumbar spinal stenosis with disc herniation | L4/5 | 0.625 |
| 43 | female | Lumbar disc herniation with radiculopathy | L5/S1 | 0.214285714 |
| 44 | male | Lumbar Disc Herniation | L4/5 | 0.235294118 |
| 45 | female | Lumbar Disc Herniation | L4/5 | 0.2 |
| 46 | female | Lumbar spinal stenosis with disc herniation | L3/4 | 0.25 |
| 47 | male | Lumbar Disc Herniation | L5/S1 | 0.5625 |
| 49 | male | Lumbar Disc Herniation | L4/5 | 0.555555556 |
| 49 | female | Lumbar spinal stenosis with disc herniation | L4/5 | 0.586206897 |
| 50 | male | Lumbar Disc Herniation | L4/5 | 0.166666667 |
| 50 | female | Lumbar disc herniation with radiculopathy | L4/5 | 0.12 |
| 51 | female | Lumbar spinal stenosis with disc herniation | L4/5 | 0.409090909 |
| 53 | male | Lumbar disc herniation with radiculopathy | L5/S1 | 0.466666667 |
| 53 | female | Lumbar spinal stenosis with disc herniation | L4/5 | 0.1 |
| 54 | male | Lumbar spinal stenosis with disc herniation | L4/5 | 0.4 |
| 54 | male | Lumbar Disc Herniation | L5/S1 | 0.071428571 |
| 55 | male | Lumbar Disc Herniation | L5/S1 | 0.130434783 |
| 55 | female | Lumbar Disc Herniation | L4/5 | 0.388888889 |
| 56 | male | Lumbar spinal stenosis with disc herniation | L4/5 | 0.555555556 |
| 56 | female | Lumbar disc herniation, lumbar spondylolisthesis | L3/4 | 0.176923077 |
| 58 | male | Lumbar Disc Herniation | L4/5 | 0.105263158 |
| 58 | male | Lumbar spinal stenosis with disc herniation | L4/5 | 0.333333333 |
| 58 | female | Lumbar spinal stenosis | L4/5 | 0.555555556 |
| 58 | female | Lumbar Disc Herniation | L4/5 | 0.2 |
| 59 | male | Lumbar spinal stenosis with disc herniation | L4/5、L5/S1 | 0.481481481 |
| 60 | male | Lumbar disc herniation with radiculopathy | L4/5 | 0.307692308 |
| 61 | male | Lumbar spinal stenosis with disc herniation | L4/5 | 0.181818182 |
| 62 | male | Lumbar Disc Herniation | L4/5 | 0.346153846 |
| 64 | female | Lumbar spinal stenosis with disc herniation | L4/5 | 0.333333333 |
| 65 | female | Lumbar Disc Herniation | L5/S1 | 0.166666667 |
| 66 | male | Lumbar Disc Herniation | L5/S1 | 0.476190476 |
| 70 | male | Lumbar Disc Herniation | L3/4 | 0.230769231 |
| 71 | male | Lumbar spinal stenosis with disc herniation | L3/4 | 0.357142857 |
| 76 | male | Lumbar spinal stenosis with disc herniation | L4/5 | 0.363636364 |
| 81 | female | Lumbar spinal stenosis with disc herniation | L4/5 | 0.2 |
| 81 | female | Lumbar spinal stenosis | L4/5 | 0.305882353 |
| 82 | male | Lumbar disc herniation, lumbar spondylolisthesis | L5/S1 | 0.285714286 |
| Age | Gender | Diagnosis | Surgical segment | CCND2 |
| 19 | male | Lumbar Disc Herniation | L4/5 | 0.956521739 |
| 25 | male | Lumbar Disc Herniation | L5/S1 | 0.857142857 |
| 28 | male | Lumbar spinal stenosis with disc herniation | L3/4 | 0.509090909 |
| 32 | male | Lumbar Disc Herniation | L5/S1 | 0.666666667 |
| 32 | male | Lumbar Disc Herniation | L5/S1 | 0.884615385 |
| 33 | male | Lumbar disc herniation with radiculopathy | L4/5 | 0.580952381 |
| 34 | male | Lumbar Disc Herniation | L4/5 | 0.647826087 |
| 36 | male | Lumbar Disc Herniation | L5/S1 | 0.333333333 |
| 41 | male | Lumbar spinal stenosis with disc herniation | L4/5 | 0.384615385 |
| 43 | female | Lumbar disc herniation with radiculopathy | L5/S1 | 0.3 |
| 50 | male | Lumbar Disc Herniation | L4/5 | 0.142857143 |
| 54 | male | Lumbar Disc Herniation | L5/S1 | 0.247058824 |
| 55 | female | Lumbar Disc Herniation | L4/5 | 0 |
| 56 | female | Lumbar disc herniation, lumbar spondylolisthesis | L3/4 | 0.130434783 |
| 58 | female | Lumbar Disc Herniation | L4/5 | 0.272727273 |
| 60 | male | Lumbar disc herniation with radiculopathy | L4/5 | 0.1 |
| 65 | female | Lumbar Disc Herniation | L5/S1 | 0.157142857 |
| 72 | female | Lumbar disc herniation, lumbar spondylolisthesis | L4/5 | 0.11125 |
| 76 | male | Lumbar spinal stenosis with disc herniation | L4/5 | 0.111111111 |
| Age | Gender | Diagnosis | Surgical segment | HIF1α |
| 15 | male | Lumbar Disc Herniation | L4/5 | 0.619047619 |
| 19 | male | Lumbar Disc Herniation | L4/5 | 0.956521739 |
| 25 | male | Lumbar Disc Herniation | L5/S1 | 0.857142857 |
| 27 | male | Lumbar spinal stenosis with disc herniation | L4/5 | 0.694444444 |
| 28 | male | Lumbar spinal stenosis with disc herniation | L3/4 | 0.685714286 |
| 28 | male | Lumbar spinal stenosis with disc herniation | L4/5 | 0.708333333 |
| 32 | male | Lumbar Disc Herniation | L5/S1 | 0.590909091 |
| 32 | male | Lumbar Disc Herniation | L5/S1 | 0.884615385 |
| 33 | male | Lumbar disc herniation with radiculopathy | L4/5 | 0.777777778 |
| 34 | male | Lumbar Disc Herniation | L4/5 | 0.666666667 |
| 36 | male | Lumbar Disc Herniation | L5/S1 | 0.307692308 |
| 37 | female | Lumbar Disc Herniation | L5/S1 | 0.871794872 |
| 41 | male | Lumbar spinal stenosis with disc herniation | L4/5 | 0.3875 |
| 43 | female | Lumbar disc herniation with radiculopathy | L5/S1 | 0.125 |
| 50 | male | Lumbar Disc Herniation | L4/5 | 0.0625 |
| 54 | male | Lumbar spinal stenosis with disc herniation | L4/5 | 0.32 |
| 54 | male | Lumbar Disc Herniation | L5/S1 | 0.15 |
| 55 | female | Lumbar Disc Herniation | L4/5 | 0.05 |
| 56 | female | Lumbar disc herniation, lumbar spondylolisthesis | L3/4 | 0.183333333 |
| 58 | female | Lumbar Disc Herniation | L4/5 | 0.214285714 |
| 58 | female | Lumbar Disc Herniation | L5/S1 | 0.304347826 |
| 59 | male | Lumbar spinal stenosis with disc herniation | L4/5、L5/S1 | 0.333333333 |
| 60 | male | Lumbar disc herniation with radiculopathy | L4/5 | 0.1 |
| 65 | female | Lumbar Disc Herniation | L5/S1 | 0.172727273 |
| 72 | female | Lumbar disc herniation, lumbar spondylolisthesis | L4/5 | 0.13485 |
| 76 | male | Lumbar spinal stenosis with disc herniation | L4/5 | 0 |
| Age | Gender | Diagnosis | Surgical segment | LDHA |
| 26 | male | Lumbar disc herniation with radiculopathy | L5/S1 | 0.88134 |
| 32 | male | Lumbar Disc Herniation | L5/S1 | 0.75 |
| 34 | male | Lumbar Disc Herniation | L4/5 | 0.69587 |
| 36 | male | Lumbar Disc Herniation | L5/S1 | 0.433518 |
| 41 | male | Lumbar spinal stenosis with disc herniation | L4/5 | 0.35884 |
| 54 | male | Lumbar Disc Herniation | L5/S1 | 0.588 |
| 65 | female | Lumbar Disc Herniation | L5/S1 | 0.1648 |
| 72 | female | Lumbar disc herniation, lumbar spondylolisthesis | L4/5 | 0.13958 |
| 76 | male | Lumbar spinal stenosis with disc herniation | L4/5 | 0.23628 |
