# Supplementary material for: SETD1A Regulates Glycolysis and Senescence of Nucleus Pulposus Cells via H3K4me3–HELZ2/PPARα‐HIF1α Axis to Drive Intervertebral Disc Degeneration
Source: Adv Sci (Weinh). 2026 Mar 31;13(34):e75105. doi: 10.1002/advs.75105 (PMC13285123; doi:10.1002/advs.75105)
Supplement: Supplementary file 5 — Supporting File 5: advs75105‐sup‐0005‐TableS4.docx. [file ADVS-13-e75105-s002.docx]

**Table S4. Lentivirus transfection**

| Target gene | Target Sequence |
| --- | --- |
| Rat shSETD1A | AGGCAAACACCGGAAATCCTT |
| Rat oeSETD1A | AGGTCGACTCTAGAGGATCCCGCCACCATGGACCAGGAAGGTGGGGGAGATGGG |
| Rat shHELZ2 | GCATAGAGCATCATAGCATCT |
| Rat oeHELZ2 | GGGAAATCTCACTAGGGCCA |
| Homo shSETD1A | CCGGTGTCAACGACTCAAAGTATATCTCGAGATATACTTTGAGTCGTTGACATTTTT |
| Homo oeSETD1A | The sequence is too long. Please contact the author for further information if needed. |
