# Supplementary material for: SETD1A Regulates Glycolysis and Senescence of Nucleus Pulposus Cells via H3K4me3–HELZ2/PPARα‐HIF1α Axis to Drive Intervertebral Disc Degeneration
Source: Adv Sci (Weinh). 2026 Mar 31;13(34):e75105. doi: 10.1002/advs.75105 (PMC13285123; doi:10.1002/advs.75105)
Supplement: Supplementary file 6 — Supporting File 6: advs75105‐sup‐0006‐TableS5.docx. [file ADVS-13-e75105-s006.docx]

**Table S5. Primers used for qPCR.**

| Target gene | Primer sequence (5'-3') | |
| --- | --- | --- |
|  | Forward | Reverse |
| Rat KMT2A | gcc aga gat tgt ctt ctt tgg tg | gat gag gca gag gtt ccc tat tt |
| Rat KMT2B | ttg ggc aga atg agt gga ca | gtt gct gag aca cga gga ca |
| Rat SETD1B | gac aca ccc aac tcc tac gg | ggg ctg acg act gga ata gc |
| Rat KDM5D | gcc ctt ttc tgc tta gag c | ggg atg ttt tct gcc tca cg |
| Rat PDK1 | gac ttc tat gcg cgc ttc tc | tca caa gca ttt act gac ccg a |
| Rat CCND2 | agt ccc gac tcc taa gac cc | cag cgg gat ggt ctc ttt ca |
| Rat HELZ2 | cgc ttc ctg acc ttc act gt | agc aat atg cac tgc cac ct |
| Rat SETD1A | gct gaa aat acg gct gag cg | ttg gtc agt gga tcg gtt gg |
| Rat LDHA | cct cag cgt ccc atg tat cc | tct gca ctc ttc ttc agg cg |
| Rat ACTB(ACTIN) | cct gta tgc ctc tgg tcg t | ctg tag cca cgc tcg gt |
| Rat HIF1α | acc cat cca tgt gac ca | ccc ggc ttg tta ggg ta |
| Rat GLUT1 | acc ctg cac cta ttg gtc t | gcc acg ata ctc aga tag gac at |
| Homo PDK1 | aag cag ttc ctg gac ttc gg | gca act ctt gcc gca gaa ac |
| Homo LDHA | acc gct tcc aat aac acg gt | tgg aca atg gag cca agt cg |
| Homo SETD1A | caa gca ccg caa gtc ct | tcc aca gct tcc tct cga t |
| Homo ACTB(ACTIN) | gga aat cgt gcg tga cat | gtg atg acc tgg ccg tt |
| Homo HIF1α | gca gca acg aca cag aaa | agc ggt ggg taa tgg ag |
| Homo GLUT1 | tgt ggg cat gtg ctt cca gta | cgg cct tta gtc tca gga act ttg |
